# Supplementary material for: Independent Membrane Binding Properties of the Caspase Generated Fragments of the Beaded Filament Structural Protein 1 (BFSP1) Involves an Amphipathic Helix
Source: Cells. 2023 Jun 7;12(12):1580. doi: 10.3390/cells12121580 (PMC10297038; doi:10.3390/cells12121580)
Supplement: Supplementary file 1 [file cells-12-01580-s001.zip › cells-2421451-supplementary.pdf]

Supplementary File for the submission "An amphipathic helix facilitates the membrane binding properties of BFSP1 and its caspase-generated C-terminal domain." by Jarrin et al.

```
Human   1    MYRRSYVFQTRKEQYEHADEASRAAEPERPAdEGWAGATSLAALQGLGERVAAHVQRARALEQRHAGLRRQLDAFQRLGE  80
Cow     1    MYRRSYVFQTRKEQYERAEEAPRAAEPDRLA-EARAAAPNLAALQGLGERVAAHVQRARALEQRHAVLRRQLDAFQRLDE  79
Rat     1    MYRRSYVFQARQERYERAQPAGPTAQPG-------GTAPGLAALQALGERVAAQVQRARALQQRHAGLRRQLYAFQRLGE  73
                                                                 Coil 1A conserved motif

Human   81   LAGPEDALARQVESNRQRVRDLEAERARLERQGTEAQRALDEFRSKYENECECQLLLKEMLERLNKEADEALLHNLRLQL  160
Cow     80   LAGPEDALARHVEGNRQRARDLAAERTRLERQGAEAQRALDEFRSKYENECECQLLLKEMLERLNKEADEALLRNLRLQI  159
Rat     74   QPGPEEALARHVEANLQRARDLAAEHARLERQEAEAQRALDEFRSKYENECECQLVLKEMLERLNKEADEALLRNLHLQL  153

Human   161  EAQFLQDDISAAKDRHKKNLLEVQTYISILQQIIHTTPPASIVTSGMREEKLLTEREVAALRSQLEEGREVLSHLQAQRV  240
Cow     160  EAQFLQDDISAAKDRYKKNLLEIQTYVTILQQIIQTTPQAAAITSGMREEKLLTEREAAALQCQLEDGREMICLLQAQRT  239
Rat     154  EAQFLQADISVAKDRYKKNLLEIQTYITILQQIIQTAPQVSLVTGGMREEKLLTEREVAALRNQLDEGREAVTHLQAQKA  233

Human   241  ELQAQTTTLEQAIKSAHECYDDEIQLYNEQIETLRKEIEETERVLEKSSYDCRQLAVAQQTLKNELDRYHRIIEIEGNRL  320
Cow     240  ELQAQTAALEQAIRDAHECYDDEIQLYNEQIDTLRKEIEEAERSLERSSYDCRQLVVVQQTLRNELDRYHRIIENEGNRL  319
Rat     234  ELQAQTTALEQAIKHAHECYDDEIQLYNGQIENLRKEIEEAERSLERSSYDCRQLAVAQQTLRNELDRYHRIIEIEGNRL  313
                                                                               Coil 2 conserved motif

Human   321  TSAFIETPIPLFTQSHGVSLSTGSGGKDLTRALQDITAAKPRQKALPKNVPRRKEIITKDKTNGALEDAPLKGLEDTKLV  400
Cow     320  SSAFIETPITLYTASHGASLSPRHGGKDLTRAVQDITAAKPRLKGLPKNLPRKKEMVAKDRADEILEETLLRGPEDMKPG  399
Rat     314  SSVFIETPISLITPSHGASLSLGSNVKDLTRAVQDITAAKPRQKALPKSLPKRKEIIAQDKVDETLEDAPLKTLQEPKAV  393

Human   401  QVVLKEESESKFESES-KEVSPLTQEGAPEDVPDGGQISKGFGKLYRKVKEKVRSPKE--PETPTELYTKERHVLVTGDA  477
Cow     400  RVVIKEEGESKLEPGD-EEASPPTQEGAPEDVPDGGKISKAFEKLGKMIKEKVKGPKE--PEPPADLYTKGRYVMVSGDG  476
Rat     394  QGELTGDGDSQLGAGGgHEVSP-TQEGGPEDVPDGSQISKAFGKLCKVVKERVSGHKEpvPEPPADLFTKGRHILVTGES  472

                                    Caspase site | Myristoylation Sequence
                       N-Terminal Domain  <----  |  ---->  C-Terminal Domain

Human   478  NYVDPRFYVSSITAKGGVAVSVAEDSVLYDGQVEPSPESPKPPLENGQVGLQEKEDGQPIDQQPIDKEIEP-----DGAE  552
Cow     477  SFVDPGFCVFSVPAKGGVVVSKGDDSVPPDSGVEPSPQQPEPPLEEGQGPPQEKEDGLKEEGGPPEGKGEPpegkgDSVK  556
Rat     473  SFVDPEFYSSSIPARGGVVVSIEEDSMHHDGHVEPSPGQPMPPVENGQGVPQGREGAHSNHQQVTDK---------NGIR  543

Human   553  LEGP---EEKREGEERDEES-RRPCAMVTPGAEEPSIPEPPKPAADQDGAEVLGTRSRSLPEKGP---------------  613
Cow     557  EEGGPP-EGKGDGVK--EEG-GPPEGKGDGVKEEGGPPEGKGDGVKKEGEPPEGKGEGLKEEEGPlqkkedgrpptphpa  632
Rat     544  AKEPKDlEEKDDDSRKDDEAgRRPCPVIIPGPDGPSTTHSQTSGSNQGGPEGPGSKSSSLLAKSP---------------  608

Human   614  ----------------------------------------------------------------------PKALAYKTVE  623
Cow     633  dkgdeknakelkglqgkqddqkeegargpcpmvapgpegpstprsqgpqvilggseghgarsgsrlarspPRKLAYEKVE  712
Rat     609  ----------------------------------------------------------------------SKALSFKKVE  618

Human   624  VVESIEKISTESIQTYEETAVIVETMIGKTKSDKKKSGEKSS-----  665
Cow     713  VMESIEKFSTESIQTYEETAVIVETMIEKTKANKKKLGEKGSSSA--  757
Rat     619  VVESIEKISTESIQTYEETSVIVETVIGKTKGNKKP-GEKSSSNAka  664
```

Figure S1: Alignment of BFSP1 protein sequences.

A

| Species | GraBCas PREDICTION (Responsible Caspase) | CasCleave 2.0 PREDICTION | CONSENSUS |
|---|---|---|---|
| Human | DVPD (2, 3, 7, 8)<br>IEPD (1, 2, 4, 5, 8)<br>EERD (2, 4, 6, 8, 9) | DVPD<br>EKED<br>IEPD<br>EERD<br>ADQD | **DVPD**<br>**IEPD**<br>**EERD** |
| Bovine | DVPD (2, 3, 7, 8)<br>SFVD (7) | DVPD<br>SFVD<br>EKED<br>DKGD | **DVPD**<br>**SFVD** |
| Rat | DVPD (1, 2, 4, 5, 8)<br>SFVD (7)<br>IEED (4, 5, 8, 9)<br>EEKD (8) | DVPD<br>IEED<br>MHHD | **DVPD** |

B

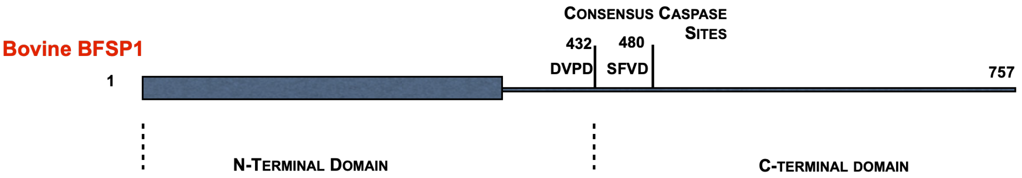


C

YVMVSGDG**SFVD**PGFCVFSVPAK

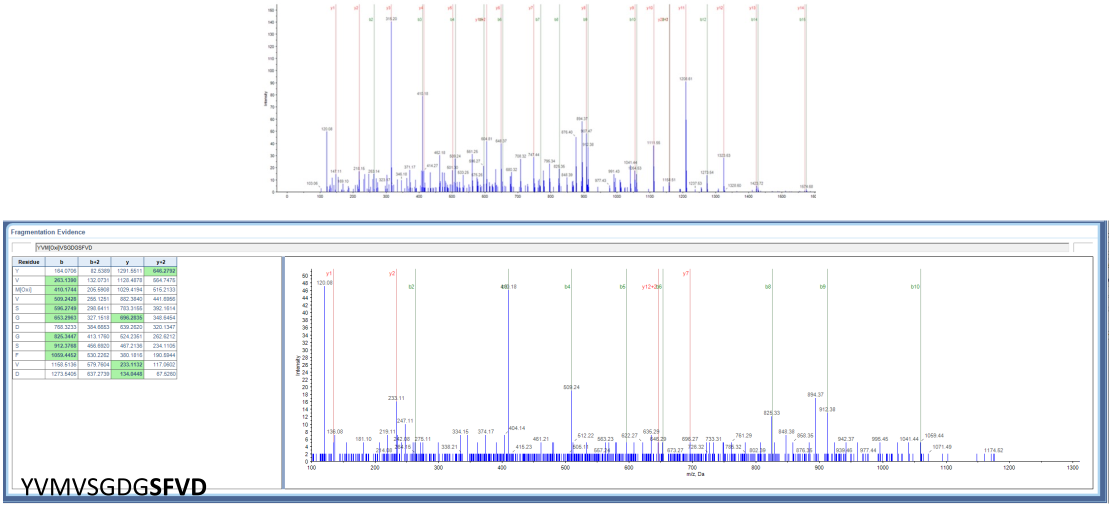


**Supplementary Figure S2.** Identification of a second Caspase Cleavage Site (SVFD) in bovine BFSP1

*Supplementary Figure S2: Identification of a second Caspase Cleavage Site (SVFD) in bovine BFSP1.*

A) Identification and conservation of potential caspase recognition sites in the C-terminal sequences distal to the myristoylation sequence in human and bovine BFSP1 using GrabCas [55] and CasCleave 2 [56].
B) Schematic comparing the predicted human and bovine caspase cleavage sites and the proteomic strategy to identify the bovine BFSP1 434 and 480 sites.
C) Identification of a second caspase cleavage site in the C-terminal sequences of bovine BFSP1 at residue 480. Bovine lens membranes from the lens cortex were subjected to trypsin cleavage, removal of SDS and then MS-MS identification of the peptides eluted in ReX-Buffer 4 [52]. The search focused on tryptic peptides lacking a C-terminal arginine or lysine. The analysis identified a new caspase site at residues 477-480 (SFVD) in bovine BFSP1.

**Helix type: alpha**

**Full sequence:**

**MYRRSYVFQTRKEQYEHADEASRAAEPERPADEGWAGATSLAALQGLGERVAAHVQRARALEQRHAGLRRQLDAFQRLGE**
**LAGPEDALARQVESNRQRVRDLEAERARLERQGTEAQRALDEFRSKYENECECQLLLKEMLERLNKEADEALLHNLRLQL**
**EAQFLQDDISAAKDRHKKNLLEVQTYISILQQIIHTTPPASIVTSGMREEKLLTEREVAALRSQLEEGREVLSHLQAQRV**
**ELQAQTTTLEQAIKSAHECYDDEIQLYNEQIETLRKEIEETERVLEKSSYDCRQLAVAQQTLKNELDRYHRIIEIEGNRL**
**TSAFIETPIPLFTQSHGVSLSTGSGGKDLTRALQDITAAKPRQKALPKNVPRRKEIITKDKTNGALEDAPLKGLEDTKLV**
**QVVLKEESESKFESESKEVSPLTQEGAPEDVPDGGQISKGFGKLYRKVKEKVRSPKEPETPTELYTKERHVLVTGDANYV**
**DPRFYVSSITAKGGVAVSVAEDSVLYDGQVEPSPESPKPPLENGQVGLQEKEDGQPIDQQPIDKEIEPDGAELEGPEEKR**
**EGEERDEESRRPCAMVTPGAEEPSIPEPPKPAADQDGAEVLGTRSRSLPEKGPPKALAYKTVEVVESIEKISTESIQTYE**
**ETAVIVETMIGKTKSDKKKSGEKSS**

**Sequence length: 665 a.a.**

**Analysis window: 18 a.a.**

---

$_{434}$**GGQISKGFGKLYRKVKEK**$_{451}$

Click to enlarge

| Physico-chemical properties | Polar residues + GLY | Nonpolar residues |
|---|---|---|
| **Hydrophobicity <H>** | **Polar residues + GLY (n / %)** | **Nonpolar residues (n / %)** |
| 0.034 | 13 / 72.22 | 5 / 27.78 |
| **Hydrophobic moment <μH>** | **Uncharged residues + GLY** | **Aromatic residues** |
| 0.539 | GLN 1, SER 1, GLY 4 | TYR 1, PHE 1, |
| **Net charge z** | **Charged residues** | **Special residues** |
| 5 | LYS 5, ARG 1, GLU 1, | CYS 0, PRO 0 |
| | **Hydrophobic face :** L I V F G Y | |

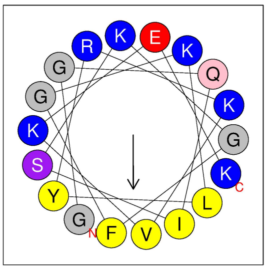


Go to screening | Manual mutation | GA mutation

---

$_{435}$**GQISKGFGKLYRKVKEKV**$_{452}$

Click to enlarge

| Physico-chemical properties | Polar residues + GLY | Nonpolar residues |
|---|---|---|
| **Hydrophobicity <H>** | **Polar residues + GLY (n / %)** | **Nonpolar residues (n / %)** |
| 0.102 | 12 / 66.67 | 6 / 33.33 |

*Supplementary Figure S3: Output from the HeliQuest analysis for the human BFSP1 sequence spanning residues 434-665.*

**Hydrophobic moment <μH>**
0.598
**Net charge z**
5

**Uncharged residues + GLY**
GLN 1, SER 1, GLY 3
**Charged residues**
LYS 5, ARG 1, GLU 1,
**Hydrophobic face :** L I V F V Y

**Aromatic residues**
TYR 1, PHE 1,
**Special residues**
CYS 0, PRO 0

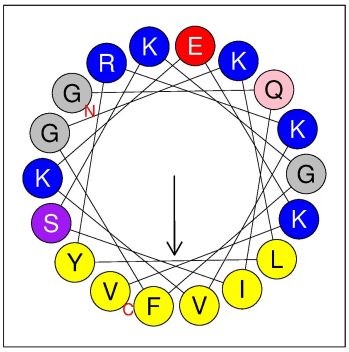


Go to screening

Manual mutation

GA mutation

---

$_{436}$**QISKGFGKLYRKVKEKVR**$_{453}$

Click to enlarge

**Physico-chemical properties**
**Hydrophobicity <H>**
0.046
**Hydrophobic moment <μH>**
0.634
**Net charge z**
6

**Polar residues + GLY**
**Polar residues + GLY (n / %)**
12 / 66.67
**Uncharged residues + GLY**
GLN 1, SER 1, GLY 2
**Charged residues**
LYS 5, ARG 2, GLU 1,
**Hydrophobic face :** L I V F V Y

**Nonpolar residues**
**Nonpolar residues (n / %)**
6 / 33.33
**Aromatic residues**
TYR 1, PHE 1,
**Special residues**
CYS 0, PRO 0

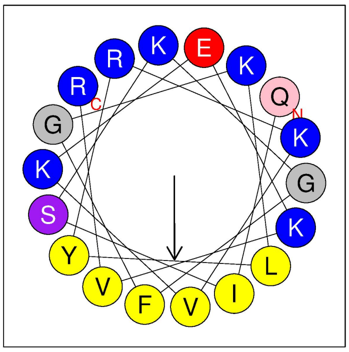


Go to screening

Manual mutation

GA mutation

---

$_{437}$**ISKGFGKLYRKVKEKVRS**$_{454}$

Click to enlarge

**Physico-chemical properties**
**Hydrophobicity <H>**
0.056
**Hydrophobic moment <μH>**
0.628
**Net charge z**
6

**Polar residues + GLY**
**Polar residues + GLY (n / %)**
12 / 66.67
**Uncharged residues + GLY**
SER 2, GLY 2
**Charged residues**
LYS 5, ARG 2, GLU 1,
**Hydrophobic face :** L I V F V Y

**Nonpolar residues**
**Nonpolar residues (n / %)**
6 / 33.33
**Aromatic residues**
TYR 1, PHE 1,
**Special residues**
CYS 0, PRO 0

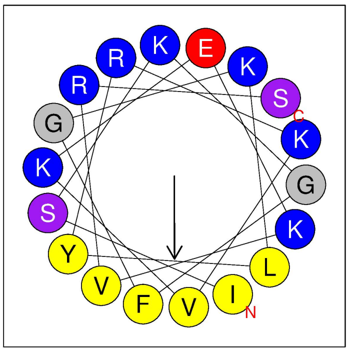


Go to screening

Manual mutation

GA mutation

---

$_{438}$**SKGFGKLYRKVKEKVRSP**$_{455}$

Click to enlarge

**Physico-chemical properties**
**Hydrophobicity <H>**
-0.004
**Hydrophobic moment <μH>**
0.575
**Net charge z**
6

**Polar residues + GLY**
**Polar residues + GLY (n / %)**
12 / 66.67
**Uncharged residues + GLY**
SER 2, GLY 2
**Charged residues**
LYS 5, ARG 2, GLU 1,
**Hydrophobic face :** L P V F V Y

**Nonpolar residues**
**Nonpolar residues (n / %)**
6 / 33.33
**Aromatic residues**
TYR 1, PHE 1,
**Special residues**
CYS 0, PRO 1

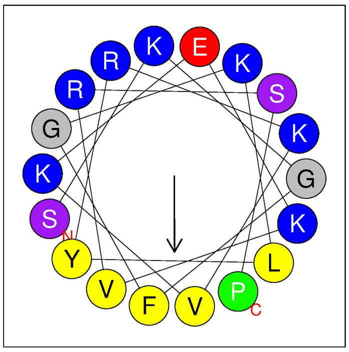


Go to screening

Manual mutation

GA mutation

---

$_{439}$**KGFGKLYRKVKEKVRSPK**$_{456}$

Click to enlarge

**Physico-chemical properties**
**Hydrophobicity <H>**
-0.057

**Polar residues + GLY**
**Polar residues + GLY (n / %)**
12 / 66.67

**Nonpolar residues**
**Nonpolar residues (n / %)**
6 / 33.33

**Hydrophobic moment <μH>**
0.560
**Net charge z**
7

**Uncharged residues + GLY**
SER 1, GLY 2
**Charged residues**
LYS 6, ARG 2, GLU 1,
**Hydrophobic face :** L P V F V Y

**Aromatic residues**
TYR 1, PHE 1,
**Special residues**
CYS 0, PRO 1

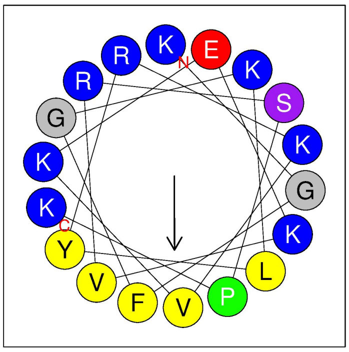

Go to screening | Manual mutation | GA mutation

---

$_{440}$**GFGKLYRKVKEKVRSPKE**$_{457}$

Click to enlarge

**Physico-chemical properties**
**Hydrophobicity <H>**
-0.038
**Hydrophobic moment <μH>**
0.541
**Net charge z**
5

**Polar residues + GLY**
**Polar residues + GLY (n / %)**
12 / 66.67
**Uncharged residues + GLY**
SER 1, GLY 2
**Charged residues**
LYS 5, ARG 2, GLU 2,
**Hydrophobic face :** L P V F V Y

**Nonpolar residues**
**Nonpolar residues (n / %)**
6 / 33.33
**Aromatic residues**
TYR 1, PHE 1,
**Special residues**
CYS 0, PRO 1

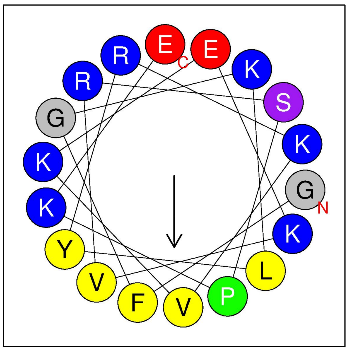

Go to screening | Manual mutation | GA mutation

---

$_{441}$**FGKLYRKVKEKVRSPKEP**$_{458}$

Click to enlarge

**Physico-chemical properties**
**Hydrophobicity <H>**
0.002
**Hydrophobic moment <μH>**
0.547
**Net charge z**
5

**Polar residues + GLY**
**Polar residues + GLY (n / %)**
11 / 61.11
**Uncharged residues + GLY**
SER 1, GLY 1
**Charged residues**
LYS 5, ARG 2, GLU 2,
**Hydrophobic face :** L P V F V Y

**Nonpolar residues**
**Nonpolar residues (n / %)**
7 / 38.89
**Aromatic residues**
TYR 1, PHE 1,
**Special residues**
CYS 0, PRO 2

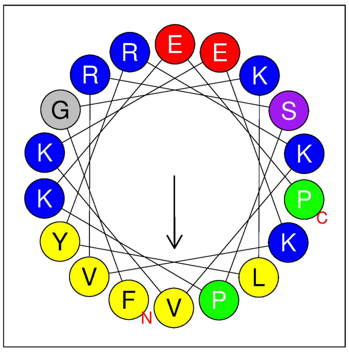

Go to screening | Manual mutation | GA mutation

---

$_{442}$**GKLYRKVKEKVRSPKEPE**$_{459}$

Click to enlarge

**Physico-chemical properties**
**Hydrophobicity <H>**
-0.133
**Hydrophobic moment <μH>**
0.423
**Net charge z**
4

**Polar residues + GLY**
**Polar residues + GLY (n / %)**
12 / 66.67
**Uncharged residues + GLY**
SER 1, GLY 1
**Charged residues**
LYS 5, ARG 2, GLU 3,
**Hydrophobic face :** none

**Nonpolar residues**
**Nonpolar residues (n / %)**
6 / 33.33
**Aromatic residues**
TYR 1,
**Special residues**
CYS 0, PRO 2

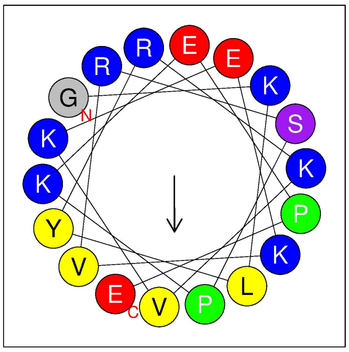

Go to screening | Manual mutation | GA mutation

---

$_{443}$**KLYRKVKEKVRSPKEPET**$_{460}$

Click to enlarge

**Physico-chemical properties**
**Hydrophobicity <H>**
-0.118

**Polar residues + GLY**
**Polar residues + GLY (n / %)**
12 / 66.67

**Nonpolar residues**
**Nonpolar residues (n / %)**
6 / 33.33

**Hydrophobic moment <μH>**
0.414
**Net charge z**
4

**Uncharged residues + GLY**
SER 1, THR 1, GLY 0
**Charged residues**
LYS 5, ARG 2, GLU 3,
**Hydrophobic face :** none

**Aromatic residues**
TYR 1,
**Special residues**
CYS 0, PRO 2

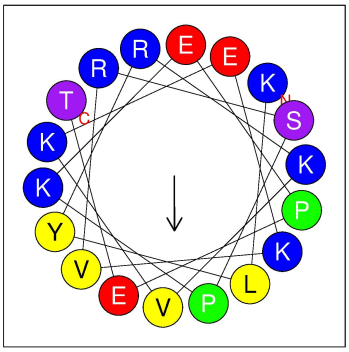

Go to screening | Manual mutation | GA mutation

---

$_{444}$**LYRKVKEKVRSPKEPETP**$_{461}$

Click to enlarge

**Physico-chemical properties**
**Hydrophobicity <H>**
-0.023
**Hydrophobic moment <μH>**
0.353
**Net charge z**
3

**Polar residues + GLY**
**Polar residues + GLY (n / %)**
11 / 61.11
**Uncharged residues + GLY**
SER 1, THR 1, GLY 0
**Charged residues**
LYS 4, ARG 2, GLU 3,
**Hydrophobic face :** none

**Nonpolar residues**
**Nonpolar residues (n / %)**
7 / 38.89
**Aromatic residues**
TYR 1,
**Special residues**
CYS 0, PRO 3

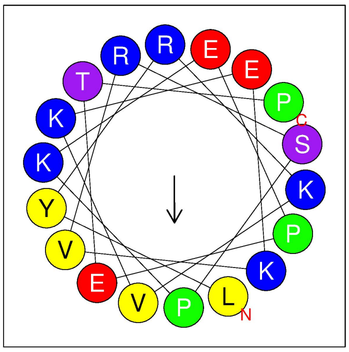

Go to screening | Manual mutation | GA mutation

---

$_{445}$**YRKVKEKVRSPKEPETPT**$_{462}$

Click to enlarge

**Physico-chemical properties**
**Hydrophobicity <H>**
-0.103
**Hydrophobic moment <μH>**
0.282
**Net charge z**
3

**Polar residues + GLY**
**Polar residues + GLY (n / %)**
12 / 66.67
**Uncharged residues + GLY**
SER 1, THR 2, GLY 0
**Charged residues**
LYS 4, ARG 2, GLU 3,
**Hydrophobic face :** none

**Nonpolar residues**
**Nonpolar residues (n / %)**
6 / 33.33
**Aromatic residues**
TYR 1,
**Special residues**
CYS 0, PRO 3

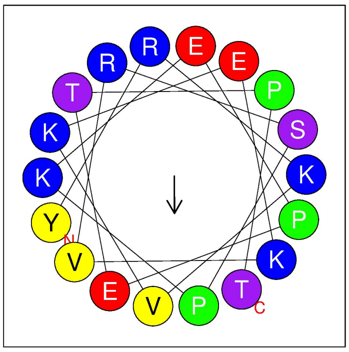

Go to screening | Manual mutation | GA mutation

---

$_{446}$**RKVKEKVRSPKEPETPTE**$_{463}$

Click to enlarge

**Physico-chemical properties**
**Hydrophobicity <H>**
-0.192
**Hydrophobic moment <μH>**
0.264
**Net charge z**
2

**Polar residues + GLY**
**Polar residues + GLY (n / %)**
13 / 72.22
**Uncharged residues + GLY**
SER 1, THR 2, GLY 0
**Charged residues**
LYS 4, ARG 2, GLU 4,
**Hydrophobic face :** none

**Nonpolar residues**
**Nonpolar residues (n / %)**
5 / 27.78
**Aromatic residues**

**Special residues**
CYS 0, PRO 3

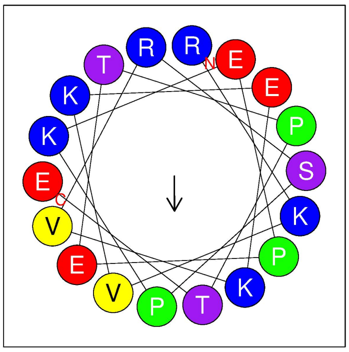

Go to screening | Manual mutation | GA mutation

---

$_{447}$**KVKEKVRSPKEPETPTEL**$_{464}$

Click to enlarge

**Physico-chemical properties**
**Hydrophobicity <H>**
-0.042

**Polar residues + GLY**
**Polar residues + GLY (n / %)**
12 / 66.67

**Nonpolar residues**
**Nonpolar residues (n / %)**
6 / 33.33

**Hydrophobic moment <μH>**
0.117
**Net charge z**
1

**Uncharged residues + GLY**
SER 1, THR 2, GLY 0
**Charged residues**
LYS 4, ARG 1, GLU 4,
**Hydrophobic face :** none

**Aromatic residues**

**Special residues**
CYS 0, PRO 3

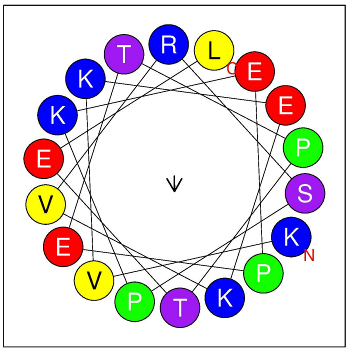

Go to screening | Manual mutation | GA mutation

---

$_{448}$**VKEKVRSPKEPETPTELY**$_{465}$

Click to enlarge

**Physico-chemical properties**
**Hydrophobicity <H>**
0.067
**Hydrophobic moment <μH>**
0.193
**Net charge z**
0

**Polar residues + GLY**
**Polar residues + GLY (n / %)**
11 / 61.11
**Uncharged residues + GLY**
SER 1, THR 2, GLY 0
**Charged residues**
LYS 3, ARG 1, GLU 4,
**Hydrophobic face :** none

**Nonpolar residues**
**Nonpolar residues (n / %)**
7 / 38.89
**Aromatic residues**
TYR 1,
**Special residues**
CYS 0, PRO 3

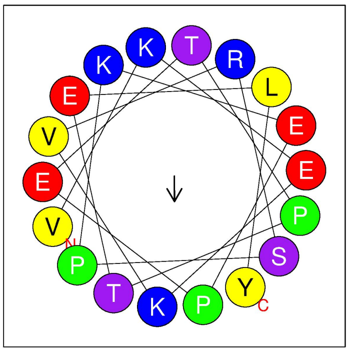

Go to screening | Manual mutation | GA mutation

---

$_{449}$**KEKVRSPKEPETPTELYT**$_{466}$

Click to enlarge

**Physico-chemical properties**
**Hydrophobicity <H>**
0.013
**Hydrophobic moment <μH>**
0.179
**Net charge z**
0

**Polar residues + GLY**
**Polar residues + GLY (n / %)**
12 / 66.67
**Uncharged residues + GLY**
SER 1, THR 3, GLY 0
**Charged residues**
LYS 3, ARG 1, GLU 4,
**Hydrophobic face :** none

**Nonpolar residues**
**Nonpolar residues (n / %)**
6 / 33.33
**Aromatic residues**
TYR 1,
**Special residues**
CYS 0, PRO 3

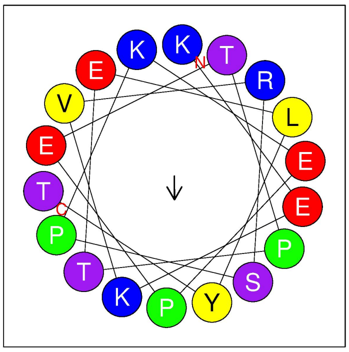

Go to screening | Manual mutation | GA mutation

---

$_{450}$**EKVRSPKEPETPTELYTK**$_{467}$

Click to enlarge

**Physico-chemical properties**
**Hydrophobicity <H>**
0.013
**Hydrophobic moment <μH>**
0.179
**Net charge z**
0

**Polar residues + GLY**
**Polar residues + GLY (n / %)**
12 / 66.67
**Uncharged residues + GLY**
SER 1, THR 3, GLY 0
**Charged residues**
LYS 3, ARG 1, GLU 4,
**Hydrophobic face :** none

**Nonpolar residues**
**Nonpolar residues (n / %)**
6 / 33.33
**Aromatic residues**
TYR 1,
**Special residues**
CYS 0, PRO 3

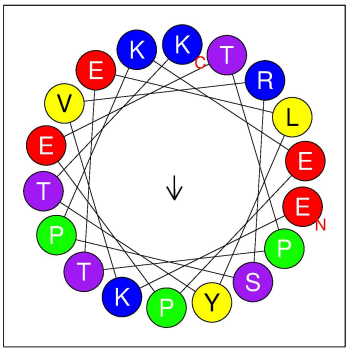

Go to screening | Manual mutation | GA mutation

---

$_{451}$**KVRSPKEPETPTELYTKE**$_{468}$

Click to enlarge

**Physico-chemical properties**
**Hydrophobicity <H>**
0.013

**Polar residues + GLY**
**Polar residues + GLY (n / %)**
12 / 66.67

**Nonpolar residues**
**Nonpolar residues (n / %)**
6 / 33.33

| | | | |
|---|---|---|---|
| **Hydrophobic moment <μH>** | **Uncharged residues + GLY** | **Aromatic residues** | 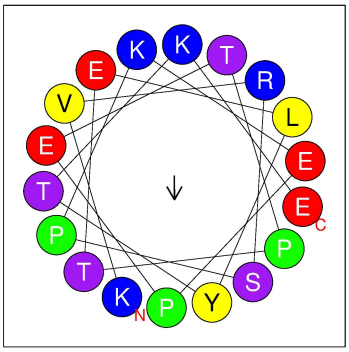 |
| 0.179 | SER 1, THR 3, GLY 0 | TYR 1, | |
| **Net charge z** | **Charged residues** | **Special residues** | |
| 0 | LYS 3, ARG 1, GLU 4, | CYS 0, PRO 3 | |
| | **Hydrophobic face :** none | | |
| Go to screening | Manual mutation | GA mutation | |

---

$_{452}$**VRSPKEPETPTELYTKER**$_{469}$

Click to enlarge

| | | | |
|---|---|---|---|
| **Physico-chemical properties** | **Polar residues + GLY** | **Nonpolar residues** | 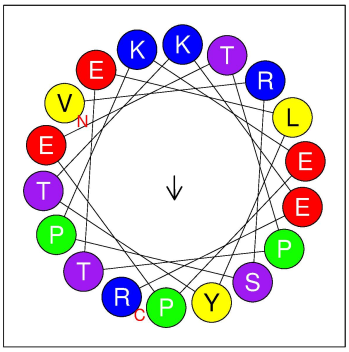 |
| **Hydrophobicity <H>** | **Polar residues + GLY (n / %)** | **Nonpolar residues (n / %)** | |
| 0.012 | 12 / 66.67 | 6 / 33.33 | |
| **Hydrophobic moment <μH>** | **Uncharged residues + GLY** | **Aromatic residues** | |
| 0.178 | SER 1, THR 3, GLY 0 | TYR 1, | |
| **Net charge z** | **Charged residues** | **Special residues** | |
| 0 | LYS 2, ARG 2, GLU 4, | CYS 0, PRO 3 | |
| | **Hydrophobic face :** none | | |
| Go to screening | Manual mutation | GA mutation | |

---

$_{453}$**RSPKEPETPTELYTKERH**$_{470}$

Click to enlarge

| | | | |
|---|---|---|---|
| **Physico-chemical properties** | **Polar residues + GLY** | **Nonpolar residues** | 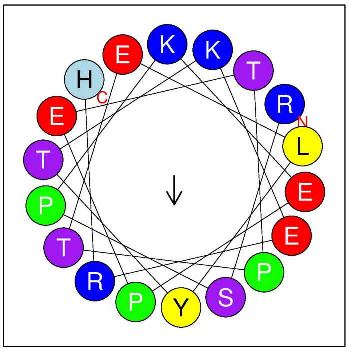 |
| **Hydrophobicity <H>** | **Polar residues + GLY (n / %)** | **Nonpolar residues (n / %)** | |
| -0.048 | 13 / 72.22 | 5 / 27.78 | |
| **Hydrophobic moment <μH>** | **Uncharged residues + GLY** | **Aromatic residues** | |
| 0.217 | HIS 1, SER 1, THR 3, GLY 0 | TYR 1, | |
| **Net charge z** | **Charged residues** | **Special residues** | |
| 0 | LYS 2, ARG 2, GLU 4, | CYS 0, PRO 3 | |
| | **Hydrophobic face :** none | | |
| Go to screening | Manual mutation | GA mutation | |

---

$_{454}$**SPKEPETPTELYTKERHV**$_{471}$

Click to enlarge

| | | | |
|---|---|---|---|
| **Physico-chemical properties** | **Polar residues + GLY** | **Nonpolar residues** | 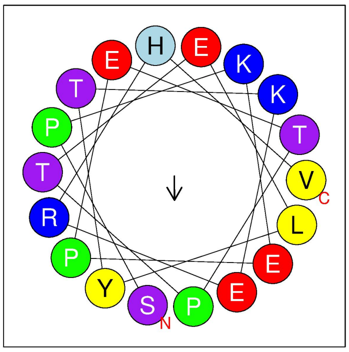 |
| **Hydrophobicity <H>** | **Polar residues + GLY (n / %)** | **Nonpolar residues (n / %)** | |
| 0.076 | 12 / 66.67 | 6 / 33.33 | |
| **Hydrophobic moment <μH>** | **Uncharged residues + GLY** | **Aromatic residues** | |
| 0.182 | HIS 1, SER 1, THR 3, GLY 0 | TYR 1, | |
| **Net charge z** | **Charged residues** | **Special residues** | |
| -1 | LYS 2, ARG 1, GLU 4, | CYS 0, PRO 3 | |
| | **Hydrophobic face :** none | | |
| Go to screening | Manual mutation | GA mutation | |

---

$_{455}$**PKEPETPTELYTKERHVL**$_{472}$

Click to enlarge

| | | |
|---|---|---|
| **Physico-chemical properties** | **Polar residues + GLY** | **Nonpolar residues** |
| **Hydrophobicity <H>** | **Polar residues + GLY (n / %)** | **Nonpolar residues (n / %)** |
| 0.172 | 11 / 61.11 | 7 / 38.89 |

**Hydrophobic moment <μH>**
0.278
**Net charge z**
-1

**Uncharged residues + GLY**
HIS 1, THR 3, GLY 0
**Charged residues**
LYS 2, ARG 1, GLU 4,
**Hydrophobic face :** none

**Aromatic residues**
TYR 1,
**Special residues**
CYS 0, PRO 3

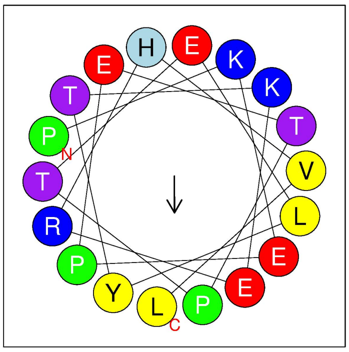

Go to screening | Manual mutation | GA mutation

---

$_{456}$**KEPETPTELYTKERHVLV**$_{473}$

Click to enlarge

**Physico-chemical properties**
**Hydrophobicity <H>**
0.200
**Hydrophobic moment <μH>**
0.271
**Net charge z**
-1

**Polar residues + GLY**
**Polar residues + GLY (n / %)**
11 / 61.11
**Uncharged residues + GLY**
HIS 1, THR 3, GLY 0
**Charged residues**
LYS 2, ARG 1, GLU 4,
**Hydrophobic face :** none

**Nonpolar residues**
**Nonpolar residues (n / %)**
7 / 38.89
**Aromatic residues**
TYR 1,
**Special residues**
CYS 0, PRO 2

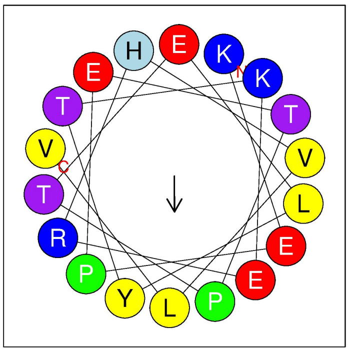

Go to screening | Manual mutation | GA mutation

---

$_{457}$**EPETPTELYTKERHVLVT**$_{474}$

Click to enlarge

**Physico-chemical properties**
**Hydrophobicity <H>**
0.269
**Hydrophobic moment <μH>**
0.208
**Net charge z**
-2

**Polar residues + GLY**
**Polar residues + GLY (n / %)**
11 / 61.11
**Uncharged residues + GLY**
HIS 1, THR 4, GLY 0
**Charged residues**
LYS 1, ARG 1, GLU 4,
**Hydrophobic face :** none

**Nonpolar residues**
**Nonpolar residues (n / %)**
7 / 38.89
**Aromatic residues**
TYR 1,
**Special residues**
CYS 0, PRO 2

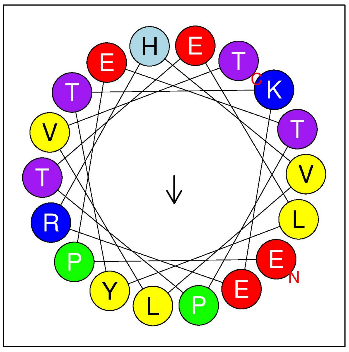

Go to screening | Manual mutation | GA mutation

---

$_{458}$**PETPTELYTKERHVLVTG**$_{475}$

Click to enlarge

**Physico-chemical properties**
**Hydrophobicity <H>**
0.305
**Hydrophobic moment <μH>**
0.232
**Net charge z**
-1

**Polar residues + GLY**
**Polar residues + GLY (n / %)**
11 / 61.11
**Uncharged residues + GLY**
HIS 1, THR 4, GLY 1
**Charged residues**
LYS 1, ARG 1, GLU 3,
**Hydrophobic face :** none

**Nonpolar residues**
**Nonpolar residues (n / %)**
7 / 38.89
**Aromatic residues**
TYR 1,
**Special residues**
CYS 0, PRO 2

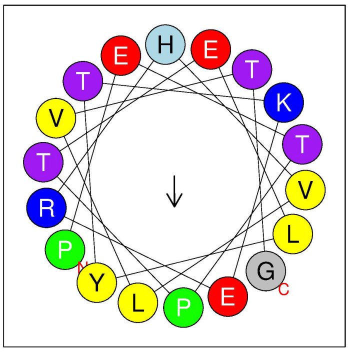

Go to screening | Manual mutation | GA mutation

---

$_{459}$**ETPTELYTKERHVLVTGD**$_{476}$

Click to enlarge

**Physico-chemical properties**
**Hydrophobicity <H>**
0.222

**Polar residues + GLY**
**Polar residues + GLY (n / %)**
12 / 66.67

**Nonpolar residues**
**Nonpolar residues (n / %)**
6 / 33.33

**Hydrophobic moment <μH>**
0.198
**Net charge z**
-2

**Uncharged residues + GLY**
HIS 1, THR 4, GLY 1
**Charged residues**
LYS 1, ARG 1, GLU 3, ASP 1,
**Hydrophobic face :** none

**Aromatic residues**
TYR 1,
**Special residues**
CYS 0, PRO 1

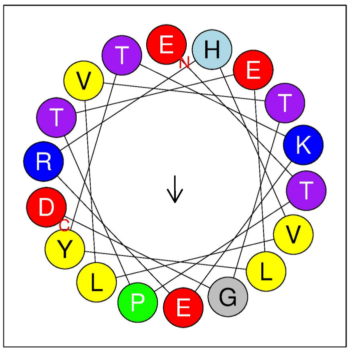


Go to screening | Manual mutation | GA mutation

---

$_{460}$**TPTELYTKERHVLVTGDA**$_{477}$

Click to enlarge

**Physico-chemical properties**
**Hydrophobicity <H>**
0.275
**Hydrophobic moment <μH>**
0.145
**Net charge z**
-1

**Polar residues + GLY**
**Polar residues + GLY (n / %)**
11 / 61.11
**Uncharged residues + GLY**
HIS 1, THR 4, GLY 1
**Charged residues**
LYS 1, ARG 1, GLU 2, ASP 1,
**Hydrophobic face :** none

**Nonpolar residues**
**Nonpolar residues (n / %)**
7 / 38.89
**Aromatic residues**
TYR 1,
**Special residues**
CYS 0, PRO 1

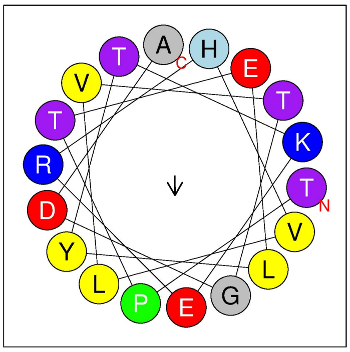


Go to screening | Manual mutation | GA mutation

---

$_{461}$**PTELYTKERHVLVTGDAN**$_{478}$

Click to enlarge

**Physico-chemical properties**
**Hydrophobicity <H>**
0.227
**Hydrophobic moment <μH>**
0.149
**Net charge z**
-1

**Polar residues + GLY**
**Polar residues + GLY (n / %)**
11 / 61.11
**Uncharged residues + GLY**
HIS 1, THR 3, ASN 1, GLY 1
**Charged residues**
LYS 1, ARG 1, GLU 2, ASP 1,
**Hydrophobic face :** none

**Nonpolar residues**
**Nonpolar residues (n / %)**
7 / 38.89
**Aromatic residues**
TYR 1,
**Special residues**
CYS 0, PRO 1

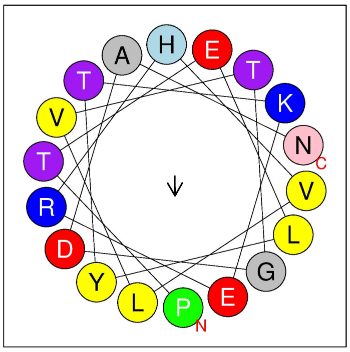


Go to screening | Manual mutation | GA mutation

---

$_{462}$**TELYTKERHVLVTGDANY**$_{479}$

Click to enlarge

**Physico-chemical properties**
**Hydrophobicity <H>**
0.241
**Hydrophobic moment <μH>**
0.162
**Net charge z**
-1

**Polar residues + GLY**
**Polar residues + GLY (n / %)**
11 / 61.11
**Uncharged residues + GLY**
HIS 1, THR 3, ASN 1, GLY 1
**Charged residues**
LYS 1, ARG 1, GLU 2, ASP 1,
**Hydrophobic face :** none

**Nonpolar residues**
**Nonpolar residues (n / %)**
7 / 38.89
**Aromatic residues**
TYR 2,
**Special residues**
CYS 0, PRO 0

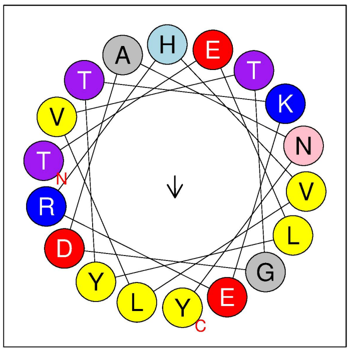


Go to screening | Manual mutation | GA mutation

---

$_{463}$**ELYTKERHVLVTGDANYV**$_{480}$

Click to enlarge

**Physico-chemical properties**
**Hydrophobicity <H>**
0.294

**Polar residues + GLY**
**Polar residues + GLY (n / %)**
10 / 55.56

**Nonpolar residues**
**Nonpolar residues (n / %)**
8 / 44.44

**Hydrophobic moment <μH>**
0.165
**Net charge z**
-1

**Uncharged residues + GLY**
HIS 1, THR 2, ASN 1, GLY 1
**Charged residues**
LYS 1, ARG 1, GLU 2, ASP 1,
**Hydrophobic face :** none

**Aromatic residues**
TYR 2,
**Special residues**
CYS 0, PRO 0

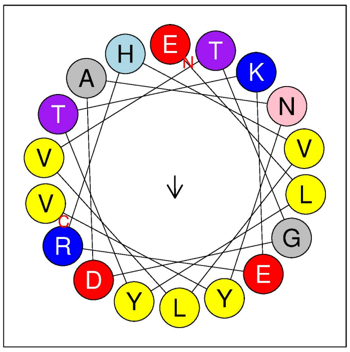


Go to screening | Manual mutation | GA mutation

---

## $_{464}$LYTKERHVLVTGDANYVD$_{481}$

Click to enlarge

**Physico-chemical properties**
**Hydrophobicity <H>**
0.287
**Hydrophobic moment <μH>**
0.172
**Net charge z**
-1

**Polar residues + GLY**
**Polar residues + GLY (n / %)**
10 / 55.56
**Uncharged residues + GLY**
HIS 1, THR 2, ASN 1, GLY 1
**Charged residues**
LYS 1, ARG 1, GLU 1, ASP 2,
**Hydrophobic face :** none

**Nonpolar residues**
**Nonpolar residues (n / %)**
8 / 44.44
**Aromatic residues**
TYR 2,
**Special residues**
CYS 0, PRO 0

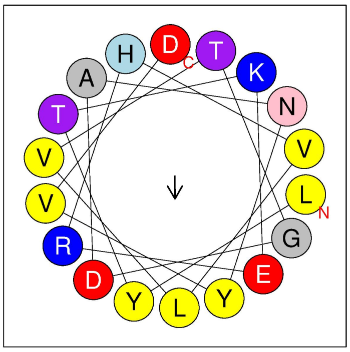


Go to screening | Manual mutation | GA mutation

---

## $_{465}$YTKERHVLVTGDANYVDP$_{482}$

Click to enlarge

**Physico-chemical properties**
**Hydrophobicity <H>**
0.232
**Hydrophobic moment <μH>**
0.173
**Net charge z**
-1

**Polar residues + GLY**
**Polar residues + GLY (n / %)**
10 / 55.56
**Uncharged residues + GLY**
HIS 1, THR 2, ASN 1, GLY 1
**Charged residues**
LYS 1, ARG 1, GLU 1, ASP 2,
**Hydrophobic face :** none

**Nonpolar residues**
**Nonpolar residues (n / %)**
8 / 44.44
**Aromatic residues**
TYR 2,
**Special residues**
CYS 0, PRO 1

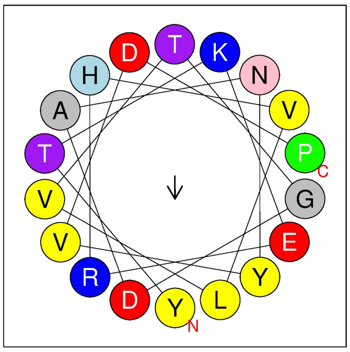


Go to screening | Manual mutation | GA mutation

---

## $_{466}$TKERHVLVTGDANYVDPR$_{483}$

Click to enlarge

**Physico-chemical properties**
**Hydrophobicity <H>**
0.123
**Hydrophobic moment <μH>**
0.064
**Net charge z**
0

**Polar residues + GLY**
**Polar residues + GLY (n / %)**
11 / 61.11
**Uncharged residues + GLY**
HIS 1, THR 2, ASN 1, GLY 1
**Charged residues**
LYS 1, ARG 2, GLU 1, ASP 2,
**Hydrophobic face :** none

**Nonpolar residues**
**Nonpolar residues (n / %)**
7 / 38.89
**Aromatic residues**
TYR 1,
**Special residues**
CYS 0, PRO 1

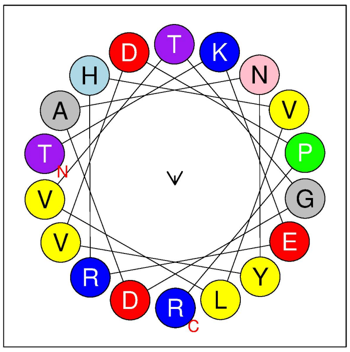


Go to screening | Manual mutation | GA mutation

---

## $_{467}$KERHVLVTGDANYVDPRF$_{484}$

Click to enlarge

**Physico-chemical properties**
**Hydrophobicity <H>**
0.208

**Polar residues + GLY**
**Polar residues + GLY (n / %)**
10 / 55.56

**Nonpolar residues**
**Nonpolar residues (n / %)**
8 / 44.44

| | | |
|---|---|---|
| **Hydrophobic moment <μH>** | **Uncharged residues + GLY** | **Aromatic residues** |
| 0.097 | HIS 1, THR 1, ASN 1, GLY 1 | TYR 1, PHE 1, |
| **Net charge z** | **Charged residues** | **Special residues** |
| 0 | LYS 1, ARG 2, GLU 1, ASP 2, | CYS 0, PRO 1 |
| | **Hydrophobic face :** none | |
| Go to screening | Manual mutation | GA mutation |

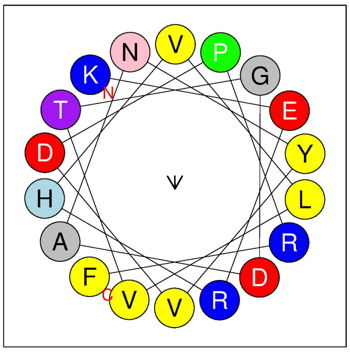

---

$_{468}$**ERHVLVTGDANYVDPRFY**$_{485}$

Click to enlarge

| **Physico-chemical properties** | **Polar residues + GLY** | **Nonpolar residues** |
|---|---|---|
| **Hydrophobicity <H>** | **Polar residues + GLY (n / %)** | **Nonpolar residues (n / %)** |
| 0.316 | 9 / 50.00 | 9 / 50.00 |
| **Hydrophobic moment <μH>** | **Uncharged residues + GLY** | **Aromatic residues** |
| 0.071 | HIS 1, THR 1, ASN 1, GLY 1 | TYR 2, PHE 1, |
| **Net charge z** | **Charged residues** | **Special residues** |
| -1 | ARG 2, GLU 1, ASP 2, | CYS 0, PRO 1 |
| | **Hydrophobic face :** none | |
| Go to screening | Manual mutation | GA mutation |

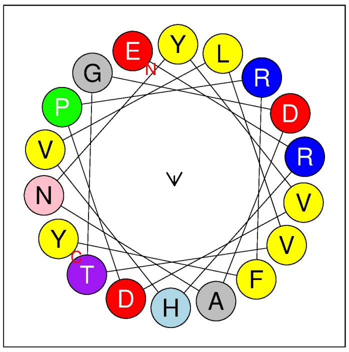

---

$_{469}$**RHVLVTGDANYVDPRFYV**$_{486}$

Click to enlarge

| **Physico-chemical properties** | **Polar residues + GLY** | **Nonpolar residues** |
|---|---|---|
| **Hydrophobicity <H>** | **Polar residues + GLY (n / %)** | **Nonpolar residues (n / %)** |
| 0.419 | 8 / 44.44 | 10 / 55.56 |
| **Hydrophobic moment <μH>** | **Uncharged residues + GLY** | **Aromatic residues** |
| 0.043 | HIS 1, THR 1, ASN 1, GLY 1 | TYR 2, PHE 1, |
| **Net charge z** | **Charged residues** | **Special residues** |
| 0 | ARG 2, ASP 2, | CYS 0, PRO 1 |
| | **Hydrophobic face :** V P G V Y L | |
| Go to screening | Manual mutation | GA mutation |

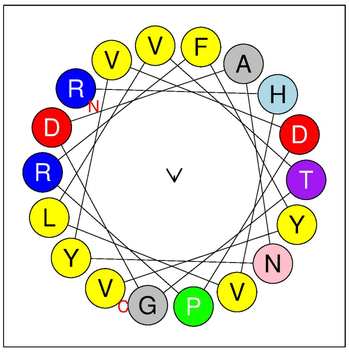

---

$_{470}$**HVLVTGDANYVDPRFYVS**$_{487}$

Click to enlarge

| **Physico-chemical properties** | **Polar residues + GLY** | **Nonpolar residues** |
|---|---|---|
| **Hydrophobicity <H>** | **Polar residues + GLY (n / %)** | **Nonpolar residues (n / %)** |
| 0.473 | 8 / 44.44 | 10 / 55.56 |
| **Hydrophobic moment <μH>** | **Uncharged residues + GLY** | **Aromatic residues** |
| 0.041 | HIS 1, SER 1, THR 1, ASN 1, GLY 1 | TYR 2, PHE 1, |
| **Net charge z** | **Charged residues** | **Special residues** |
| -1 | ARG 1, ASP 2, | CYS 0, PRO 1 |
| | **Hydrophobic face :** V P G V Y L | |
| Go to screening | Manual mutation | GA mutation |

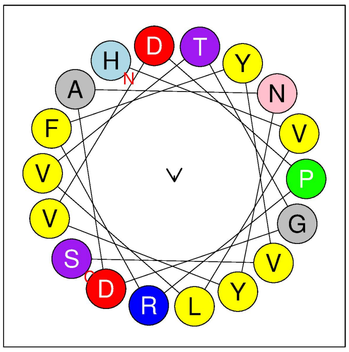

---

$_{471}$**VLVTGDANYVDPRFYVSS**$_{488}$

Click to enlarge

| **Physico-chemical properties** | **Polar residues + GLY** | **Nonpolar residues** |
|---|---|---|
| **Hydrophobicity <H>** | **Polar residues + GLY (n / %)** | **Nonpolar residues (n / %)** |
| 0.464 | 8 / 44.44 | 10 / 55.56 |

**Hydrophobic moment <μH>**
0.049
**Net charge z**
-1

**Uncharged residues + GLY**
SER 2, THR 1, ASN 1, GLY 1
**Charged residues**
ARG 1, ASP 2,
**Hydrophobic face :** V P G V Y L

**Aromatic residues**
TYR 2, PHE 1,
**Special residues**
CYS 0, PRO 1

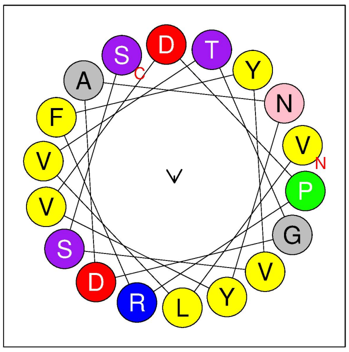

Go to screening | Manual mutation | GA mutation

---

$_{472}$**LVTGDANYVDPRFYVSSI**$_{489}$

Click to enlarge

**Physico-chemical properties**
**Hydrophobicity <H>**
0.496
**Hydrophobic moment <μH>**
0.052
**Net charge z**
-1

**Polar residues + GLY**
**Polar residues + GLY (n / %)**
8 / 44.44
**Uncharged residues + GLY**
SER 2, THR 1, ASN 1, GLY 1
**Charged residues**
ARG 1, ASP 2,
**Hydrophobic face :** I P G V Y L

**Nonpolar residues**
**Nonpolar residues (n / %)**
10 / 55.56
**Aromatic residues**
TYR 2, PHE 1,
**Special residues**
CYS 0, PRO 1

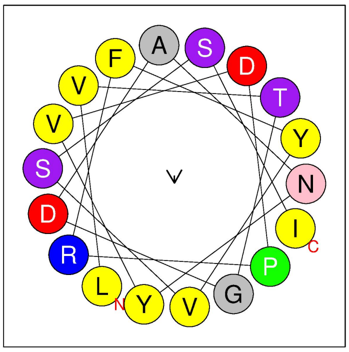

Go to screening | Manual mutation | GA mutation

---

$_{473}$**VTGDANYVDPRFYVSSIT**$_{490}$

Click to enlarge

**Physico-chemical properties**
**Hydrophobicity <H>**
0.416
**Hydrophobic moment <μH>**
0.046
**Net charge z**
-1

**Polar residues + GLY**
**Polar residues + GLY (n / %)**
9 / 50.00
**Uncharged residues + GLY**
SER 2, THR 2, ASN 1, GLY 1
**Charged residues**
ARG 1, ASP 2,
**Hydrophobic face :** I P G V Y

**Nonpolar residues**
**Nonpolar residues (n / %)**
9 / 50.00
**Aromatic residues**
TYR 2, PHE 1,
**Special residues**
CYS 0, PRO 1

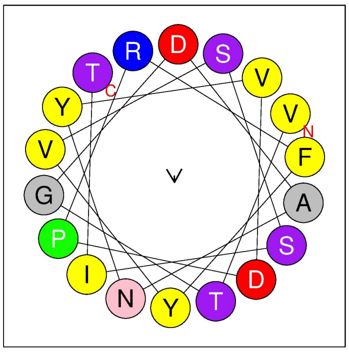

Go to screening | Manual mutation | GA mutation

---

$_{474}$**TGDANYVDPRFYVSSITA**$_{491}$

Click to enlarge

**Physico-chemical properties**
**Hydrophobicity <H>**
0.366
**Hydrophobic moment <μH>**
0.083
**Net charge z**
-1

**Polar residues + GLY**
**Polar residues + GLY (n / %)**
9 / 50.00
**Uncharged residues + GLY**
SER 2, THR 2, ASN 1, GLY 1
**Charged residues**
ARG 1, ASP 2,
**Hydrophobic face :** I P G V Y

**Nonpolar residues**
**Nonpolar residues (n / %)**
9 / 50.00
**Aromatic residues**
TYR 2, PHE 1,
**Special residues**
CYS 0, PRO 1

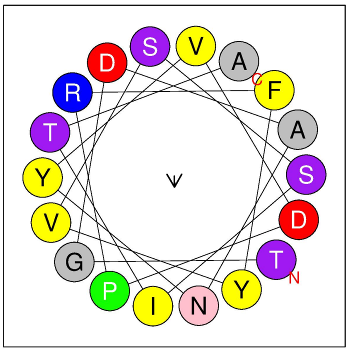

Go to screening | Manual mutation | GA mutation

---

$_{475}$**GDANYVDPRFYVSSITAK**$_{492}$

Click to enlarge

**Physico-chemical properties**
**Hydrophobicity <H>**
0.296

**Polar residues + GLY**
**Polar residues + GLY (n / %)**
9 / 50.00

**Nonpolar residues**
**Nonpolar residues (n / %)**
9 / 50.00

**Hydrophobic moment <μH>**
0.066
**Net charge z**
0

**Uncharged residues + GLY**
SER 2, THR 1, ASN 1, GLY 1
**Charged residues**
LYS 1, ARG 1, ASP 2,
**Hydrophobic face :** I P G V Y

**Aromatic residues**
TYR 2, PHE 1,
**Special residues**
CYS 0, PRO 1

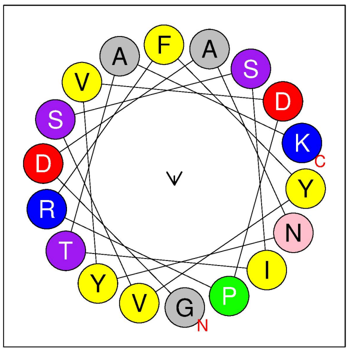

Go to screening | Manual mutation | GA mutation

---

$_{476}$**DANYVDPRFYVSSITAKG**$_{493}$

Click to enlarge

**Physico-chemical properties**
**Hydrophobicity <H>**
0.296
**Hydrophobic moment <μH>**
0.066
**Net charge z**
0

**Polar residues + GLY**
**Polar residues + GLY (n / %)**
9 / 50.00
**Uncharged residues + GLY**
SER 2, THR 1, ASN 1, GLY 1
**Charged residues**
LYS 1, ARG 1, ASP 2,
**Hydrophobic face :** I P G V Y

**Nonpolar residues**
**Nonpolar residues (n / %)**
9 / 50.00
**Aromatic residues**
TYR 2, PHE 1,
**Special residues**
CYS 0, PRO 1

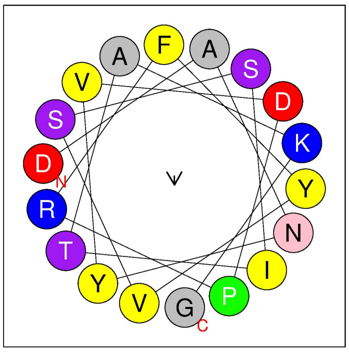

Go to screening | Manual mutation | GA mutation

---

$_{477}$**ANYVDPRFYVSSITAKGG**$_{494}$

Click to enlarge

**Physico-chemical properties**
**Hydrophobicity <H>**
0.339
**Hydrophobic moment <μH>**
0.076
**Net charge z**
1

**Polar residues + GLY**
**Polar residues + GLY (n / %)**
9 / 50.00
**Uncharged residues + GLY**
SER 2, THR 1, ASN 1, GLY 2
**Charged residues**
LYS 1, ARG 1, ASP 1,
**Hydrophobic face :** I P G V Y

**Nonpolar residues**
**Nonpolar residues (n / %)**
9 / 50.00
**Aromatic residues**
TYR 2, PHE 1,
**Special residues**
CYS 0, PRO 1

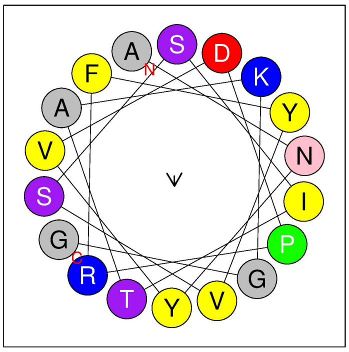

Go to screening | Manual mutation | GA mutation

---

$_{478}$**NYVDPRFYVSSITAKGGV**$_{495}$

Click to enlarge

**Physico-chemical properties**
**Hydrophobicity <H>**
0.389
**Hydrophobic moment <μH>**
0.032
**Net charge z**
1

**Polar residues + GLY**
**Polar residues + GLY (n / %)**
9 / 50.00
**Uncharged residues + GLY**
SER 2, THR 1, ASN 1, GLY 2
**Charged residues**
LYS 1, ARG 1, ASP 1,
**Hydrophobic face :** I P G V Y

**Nonpolar residues**
**Nonpolar residues (n / %)**
9 / 50.00
**Aromatic residues**
TYR 2, PHE 1,
**Special residues**
CYS 0, PRO 1

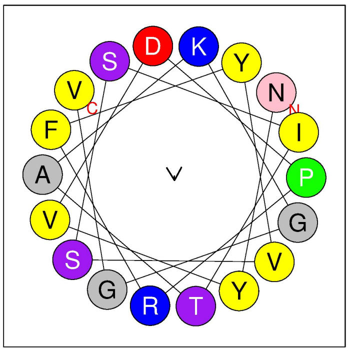

Go to screening | Manual mutation | GA mutation

---

$_{479}$**YVDPRFYVSSITAKGGVA**$_{496}$

Click to enlarge

**Physico-chemical properties**
**Hydrophobicity <H>**
0.440

**Polar residues + GLY**
**Polar residues + GLY (n / %)**
8 / 44.44

**Nonpolar residues**
**Nonpolar residues (n / %)**
10 / 55.56

| | | |
|---|---|---|
| **Hydrophobic moment <μH>** | **Uncharged residues + GLY** | **Aromatic residues** |
| 0.039 | SER 2, THR 1, GLY 2 | TYR 2, PHE 1, |
| **Net charge z** | **Charged residues** | **Special residues** |
| 1 | LYS 1, ARG 1, ASP 1, | CYS 0, PRO 1 |
| | **Hydrophobic face :** Y A I P G V Y | |
| Go to screening | Manual mutation | GA mutation |

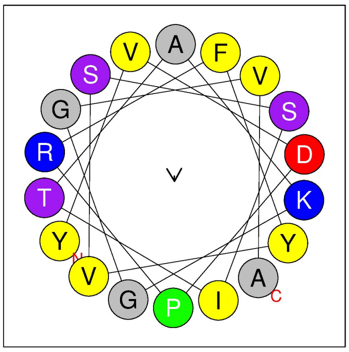

---

**$_{480}$VDPRFYVSSITAKGGVAV$_{497}$**

Click to enlarge

| | | |
|---|---|---|
| **Physico-chemical properties** | **Polar residues + GLY** | **Nonpolar residues** |
| **Hydrophobicity <H>** | **Polar residues + GLY (n / %)** | **Nonpolar residues (n / %)** |
| 0.454 | 8 / 44.44 | 10 / 55.56 |
| **Hydrophobic moment <μH>** | **Uncharged residues + GLY** | **Aromatic residues** |
| 0.048 | SER 2, THR 1, GLY 2 | TYR 1, PHE 1, |
| **Net charge z** | **Charged residues** | **Special residues** |
| 1 | LYS 1, ARG 1, ASP 1, | CYS 0, PRO 1 |
| | **Hydrophobic face :** Y A I P G V V | |
| Go to screening | Manual mutation | GA mutation |

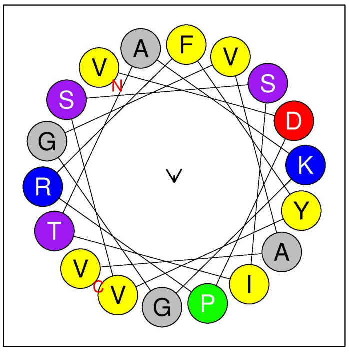

---

**$_{481}$DPRFYVSSITAKGGVAVS$_{498}$**

Click to enlarge

| | | |
|---|---|---|
| **Physico-chemical properties** | **Polar residues + GLY** | **Nonpolar residues** |
| **Hydrophobicity <H>** | **Polar residues + GLY (n / %)** | **Nonpolar residues (n / %)** |
| 0.384 | 9 / 50.00 | 9 / 50.00 |
| **Hydrophobic moment <μH>** | **Uncharged residues + GLY** | **Aromatic residues** |
| 0.113 | SER 3, THR 1, GLY 2 | TYR 1, PHE 1, |
| **Net charge z** | **Charged residues** | **Special residues** |
| 1 | LYS 1, ARG 1, ASP 1, | CYS 0, PRO 1 |
| | **Hydrophobic face :** Y A I P G V V | |
| Go to screening | Manual mutation | GA mutation |

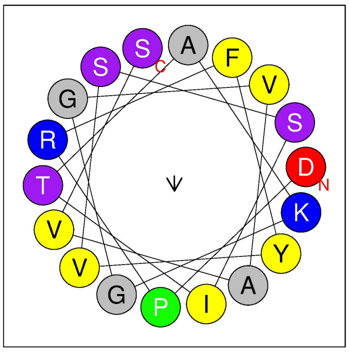

---

**$_{482}$PRFYVSSITAKGGVAVSV$_{499}$**

Click to enlarge

| | | |
|---|---|---|
| **Physico-chemical properties** | **Polar residues + GLY** | **Nonpolar residues** |
| **Hydrophobicity <H>** | **Polar residues + GLY (n / %)** | **Nonpolar residues (n / %)** |
| 0.495 | 8 / 44.44 | 10 / 55.56 |
| **Hydrophobic moment <μH>** | **Uncharged residues + GLY** | **Aromatic residues** |
| 0.152 | SER 3, THR 1, GLY 2 | TYR 1, PHE 1, |
| **Net charge z** | **Charged residues** | **Special residues** |
| 2 | LYS 1, ARG 1, | CYS 0, PRO 1 |
| | **Hydrophobic face :** Y A I P G V V | |
| Go to screening | Manual mutation | GA mutation |

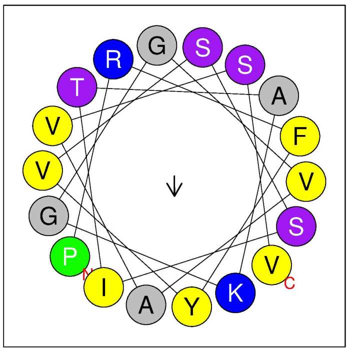

---

**$_{483}$RFYVSSITAKGGVAVSVA$_{500}$**

Click to enlarge

| | | |
|---|---|---|
| **Physico-chemical properties** | **Polar residues + GLY** | **Nonpolar residues** |
| **Hydrophobicity <H>** | **Polar residues + GLY (n / %)** | **Nonpolar residues (n / %)** |
| 0.472 | 8 / 44.44 | 10 / 55.56 |

| | | |
|---|---|---|
| **Hydrophobic moment <μH>** | **Uncharged residues + GLY** | **Aromatic residues** |
| 0.140 | SER 3, THR 1, GLY 2 | TYR 1, PHE 1, |
| **Net charge z** | **Charged residues** | **Special residues** |
| 2 | LYS 1, ARG 1, | CYS 0, PRO 0 |
| | **Hydrophobic face :** Y A I A G V V | |

Go to screening Manual mutation GA mutation

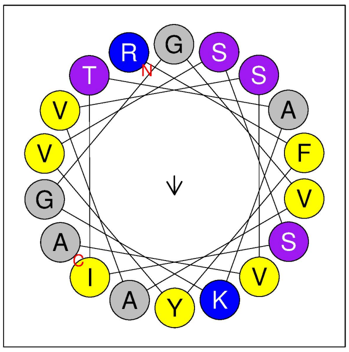

---

$_{484}$**FYVSSITAKGGVAVSVAE**$_{501}$

Click to enlarge

| | | |
|---|---|---|
| **Physico-chemical properties** | **Polar residues + GLY** | **Nonpolar residues** |
| **Hydrophobicity <H>** | **Polar residues + GLY (n / %)** | **Nonpolar residues (n / %)** |
| 0.493 | 8 / 44.44 | 10 / 55.56 |
| **Hydrophobic moment <μH>** | **Uncharged residues + GLY** | **Aromatic residues** |
| 0.120 | SER 3, THR 1, GLY 2 | TYR 1, PHE 1, |
| **Net charge z** | **Charged residues** | **Special residues** |
| 0 | LYS 1, GLU 1, | CYS 0, PRO 0 |
| | **Hydrophobic face :** Y A I A G V V | |

Go to screening Manual mutation GA mutation

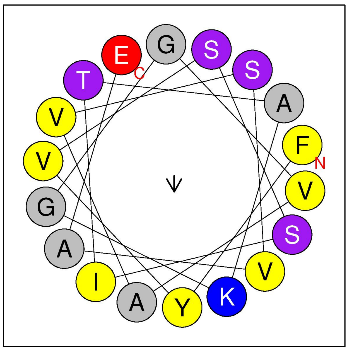

---

$_{485}$**YVSSITAKGGVAVSVAED**$_{502}$

Click to enlarge

| | | |
|---|---|---|
| **Physico-chemical properties** | **Polar residues + GLY** | **Nonpolar residues** |
| **Hydrophobicity <H>** | **Polar residues + GLY (n / %)** | **Nonpolar residues (n / %)** |
| 0.351 | 9 / 50.00 | 9 / 50.00 |
| **Hydrophobic moment <μH>** | **Uncharged residues + GLY** | **Aromatic residues** |
| 0.207 | SER 3, THR 1, GLY 2 | TYR 1, |
| **Net charge z** | **Charged residues** | **Special residues** |
| -1 | LYS 1, GLU 1, ASP 1, | CYS 0, PRO 0 |
| | **Hydrophobic face :** Y A I A G V V | |

Go to screening Manual mutation GA mutation

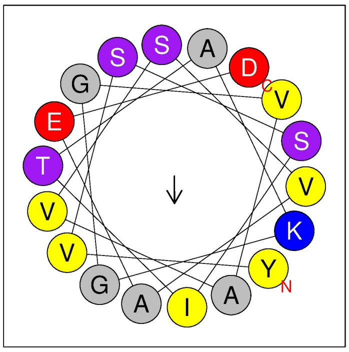

---

$_{486}$**VSSITAKGGVAVSVAEDS**$_{503}$

Click to enlarge

| | | |
|---|---|---|
| **Physico-chemical properties** | **Polar residues + GLY** | **Nonpolar residues** |
| **Hydrophobicity <H>** | **Polar residues + GLY (n / %)** | **Nonpolar residues (n / %)** |
| 0.295 | 10 / 55.56 | 8 / 44.44 |
| **Hydrophobic moment <μH>** | **Uncharged residues + GLY** | **Aromatic residues** |
| 0.172 | SER 4, THR 1, GLY 2 | |
| **Net charge z** | **Charged residues** | **Special residues** |
| -1 | LYS 1, GLU 1, ASP 1, | CYS 0, PRO 0 |
| | **Hydrophobic face :** A I A G V V | |

Go to screening Manual mutation GA mutation

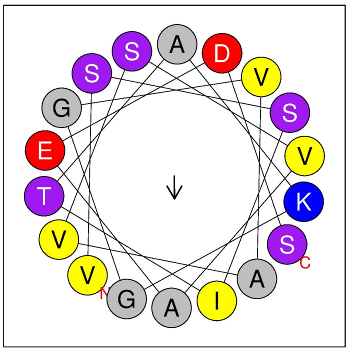

---

$_{487}$**SSITAKGGVAVSVAEDSV**$_{504}$

Click to enlarge

| | | |
|---|---|---|
| **Physico-chemical properties** | **Polar residues + GLY** | **Nonpolar residues** |
| **Hydrophobicity <H>** | **Polar residues + GLY (n / %)** | **Nonpolar residues (n / %)** |
| 0.295 | 10 / 55.56 | 8 / 44.44 |

| | | |
|---|---|---|
| **Hydrophobic moment <μH>** | **Uncharged residues + GLY** | **Aromatic residues** |
| 0.172 | SER 4, THR 1, GLY 2 | |
| **Net charge z** | **Charged residues** | **Special residues** |
| -1 | LYS 1, GLU 1, ASP 1, | CYS 0, PRO 0 |
| | **Hydrophobic face :** A I A G V V | |
| Go to screening | Manual mutation | GA mutation |

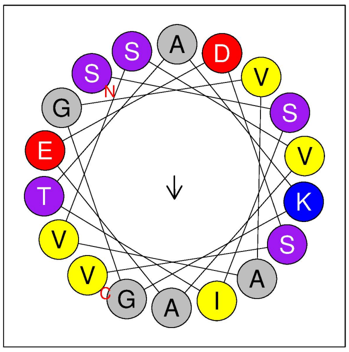

---

$_{488}$**SITAKGGVAVSVAEDSVL**$_{505}$

Click to enlarge

| | | |
|---|---|---|
| **Physico-chemical properties** | **Polar residues + GLY** | **Nonpolar residues** |
| **Hydrophobicity <H>** | **Polar residues + GLY (n / %)** | **Nonpolar residues (n / %)** |
| 0.392 | 9 / 50.00 | 9 / 50.00 |
| **Hydrophobic moment <μH>** | **Uncharged residues + GLY** | **Aromatic residues** |
| 0.114 | SER 3, THR 1, GLY 2 | |
| **Net charge z** | **Charged residues** | **Special residues** |
| -1 | LYS 1, GLU 1, ASP 1, | CYS 0, PRO 0 |
| | **Hydrophobic face :** A I A G V V | |
| Go to screening | Manual mutation | GA mutation |

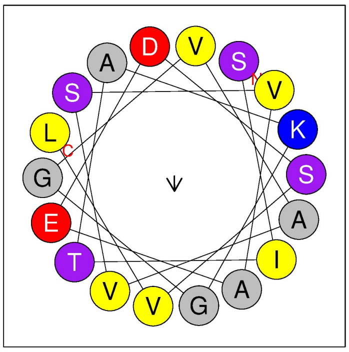

---

$_{489}$**ITAKGGVAVSVAEDSVLY**$_{506}$

Click to enlarge

| | | |
|---|---|---|
| **Physico-chemical properties** | **Polar residues + GLY** | **Nonpolar residues** |
| **Hydrophobicity <H>** | **Polar residues + GLY (n / %)** | **Nonpolar residues (n / %)** |
| 0.447 | 8 / 44.44 | 10 / 55.56 |
| **Hydrophobic moment <μH>** | **Uncharged residues + GLY** | **Aromatic residues** |
| 0.071 | SER 2, THR 1, GLY 2 | TYR 1, |
| **Net charge z** | **Charged residues** | **Special residues** |
| -1 | LYS 1, GLU 1, ASP 1, | CYS 0, PRO 0 |
| | **Hydrophobic face :** A I A G V V | |
| Go to screening | Manual mutation | GA mutation |

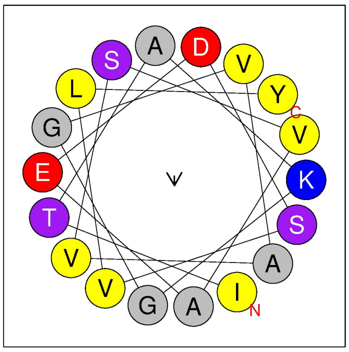

---

$_{490}$**TAKGGVAVSVAEDSVLYD**$_{507}$

Click to enlarge

| | | |
|---|---|---|
| **Physico-chemical properties** | **Polar residues + GLY** | **Nonpolar residues** |
| **Hydrophobicity <H>** | **Polar residues + GLY (n / %)** | **Nonpolar residues (n / %)** |
| 0.304 | 9 / 50.00 | 9 / 50.00 |
| **Hydrophobic moment <μH>** | **Uncharged residues + GLY** | **Aromatic residues** |
| 0.087 | SER 2, THR 1, GLY 2 | TYR 1, |
| **Net charge z** | **Charged residues** | **Special residues** |
| -2 | LYS 1, GLU 1, ASP 2, | CYS 0, PRO 0 |
| | **Hydrophobic face :** none | |
| Go to screening | Manual mutation | GA mutation |

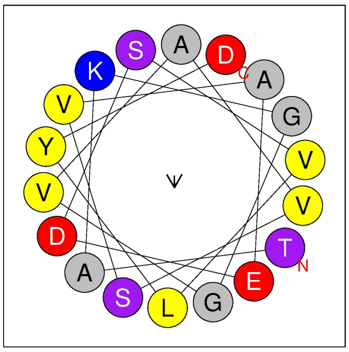

---

$_{491}$**AKGGVAVSVAEDSVLYDG**$_{508}$

Click to enlarge

| | | |
|---|---|---|
| **Physico-chemical properties** | **Polar residues + GLY** | **Nonpolar residues** |
| **Hydrophobicity <H>** | **Polar residues + GLY (n / %)** | **Nonpolar residues (n / %)** |
| 0.290 | 9 / 50.00 | 9 / 50.00 |

**Hydrophobic moment <μH>**
0.080
**Net charge z**
-2

**Uncharged residues + GLY**
SER 2, GLY 3
**Charged residues**
LYS 1, GLU 1, ASP 2,
**Hydrophobic face :** none

**Aromatic residues**
TYR 1,
**Special residues**
CYS 0, PRO 0

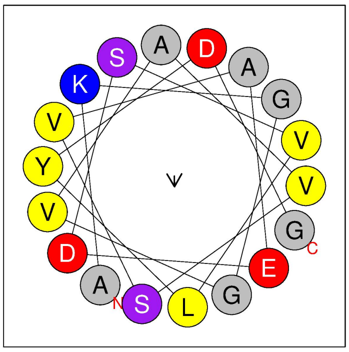

Go to screening    Manual mutation    GA mutation

---

$_{492}$**KGGVAVSVAEDSVLYDGQ**$_{509}$

Click to enlarge

**Physico-chemical properties**
**Hydrophobicity <H>**
0.261
**Hydrophobic moment <μH>**
0.058
**Net charge z**
-2

**Polar residues + GLY**
**Polar residues + GLY (n / %)**
10 / 55.56
**Uncharged residues + GLY**
GLN 1, SER 2, GLY 3
**Charged residues**
LYS 1, GLU 1, ASP 2,
**Hydrophobic face :** none

**Nonpolar residues**
**Nonpolar residues (n / %)**
8 / 44.44
**Aromatic residues**
TYR 1,
**Special residues**
CYS 0, PRO 0

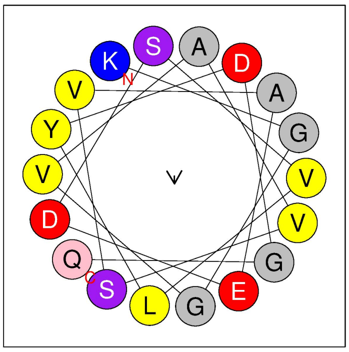

Go to screening    Manual mutation    GA mutation

---

$_{493}$**GGVAVSVAEDSVLYDGQV**$_{510}$

Click to enlarge

**Physico-chemical properties**
**Hydrophobicity <H>**
0.383
**Hydrophobic moment <μH>**
0.077
**Net charge z**
-3

**Polar residues + GLY**
**Polar residues + GLY (n / %)**
9 / 50.00
**Uncharged residues + GLY**
GLN 1, SER 2, GLY 3
**Charged residues**
GLU 1, ASP 2,
**Hydrophobic face :** none

**Nonpolar residues**
**Nonpolar residues (n / %)**
9 / 50.00
**Aromatic residues**
TYR 1,
**Special residues**
CYS 0, PRO 0

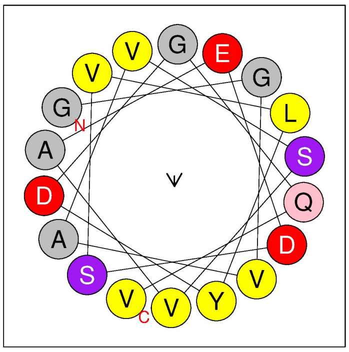

Go to screening    Manual mutation    GA mutation

---

$_{494}$**GVAVSVAEDSVLYDGQVE**$_{511}$

Click to enlarge

**Physico-chemical properties**
**Hydrophobicity <H>**
0.348
**Hydrophobic moment <μH>**
0.101
**Net charge z**
-4

**Polar residues + GLY**
**Polar residues + GLY (n / %)**
9 / 50.00
**Uncharged residues + GLY**
GLN 1, SER 2, GLY 2
**Charged residues**
GLU 2, ASP 2,
**Hydrophobic face :** none

**Nonpolar residues**
**Nonpolar residues (n / %)**
9 / 50.00
**Aromatic residues**
TYR 1,
**Special residues**
CYS 0, PRO 0

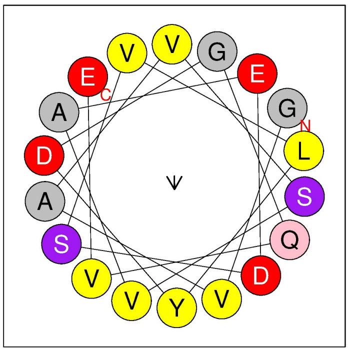

Go to screening    Manual mutation    GA mutation

---

$_{495}$**VAVSVAEDSVLYDGQVEP**$_{512}$

Click to enlarge

**Physico-chemical properties**
**Hydrophobicity <H>**
0.388

**Polar residues + GLY**
**Polar residues + GLY (n / %)**
8 / 44.44

**Nonpolar residues**
**Nonpolar residues (n / %)**
10 / 55.56

**Hydrophobic moment <μH>**
0.087
**Net charge z**
-4

**Uncharged residues + GLY**
GLN 1, SER 2, GLY 1
**Charged residues**
GLU 2, ASP 2,
**Hydrophobic face :** none

**Aromatic residues**
TYR 1,
**Special residues**
CYS 0, PRO 1

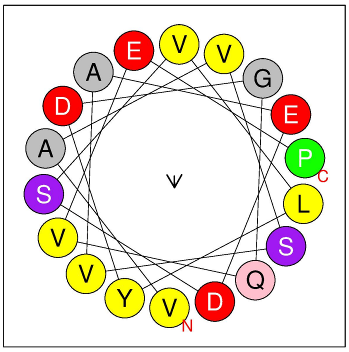

Go to screening | Manual mutation | GA mutation

---

$_{496}$**AVSVAEDSVLYDGQVEPS**$_{513}$

Click to enlarge

**Physico-chemical properties**
**Hydrophobicity <H>**
0.318
**Hydrophobic moment <μH>**
0.017
**Net charge z**
-4

**Polar residues + GLY**
**Polar residues + GLY (n / %)**
9 / 50.00
**Uncharged residues + GLY**
GLN 1, SER 3, GLY 1
**Charged residues**
GLU 2, ASP 2,
**Hydrophobic face :** none

**Nonpolar residues**
**Nonpolar residues (n / %)**
9 / 50.00
**Aromatic residues**
TYR 1,
**Special residues**
CYS 0, PRO 1

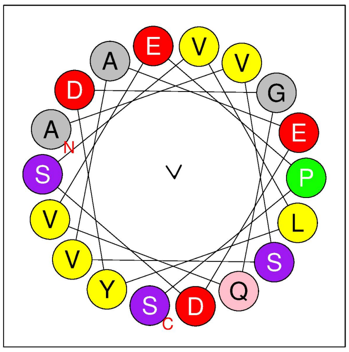

Go to screening | Manual mutation | GA mutation

---

$_{497}$**VSVAEDSVLYDGQVEPSP**$_{514}$

Click to enlarge

**Physico-chemical properties**
**Hydrophobicity <H>**
0.341
**Hydrophobic moment <μH>**
0.023
**Net charge z**
-4

**Polar residues + GLY**
**Polar residues + GLY (n / %)**
9 / 50.00
**Uncharged residues + GLY**
GLN 1, SER 3, GLY 1
**Charged residues**
GLU 2, ASP 2,
**Hydrophobic face :** none

**Nonpolar residues**
**Nonpolar residues (n / %)**
9 / 50.00
**Aromatic residues**
TYR 1,
**Special residues**
CYS 0, PRO 2

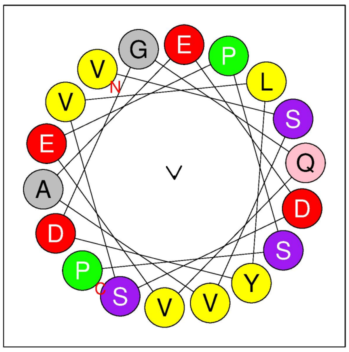

Go to screening | Manual mutation | GA mutation

---

$_{498}$**SVAEDSVLYDGQVEPSPE**$_{515}$

Click to enlarge

**Physico-chemical properties**
**Hydrophobicity <H>**
0.237
**Hydrophobic moment <μH>**
0.123
**Net charge z**
-5

**Polar residues + GLY**
**Polar residues + GLY (n / %)**
10 / 55.56
**Uncharged residues + GLY**
GLN 1, SER 3, GLY 1
**Charged residues**
GLU 3, ASP 2,
**Hydrophobic face :** none

**Nonpolar residues**
**Nonpolar residues (n / %)**
8 / 44.44
**Aromatic residues**
TYR 1,
**Special residues**
CYS 0, PRO 2

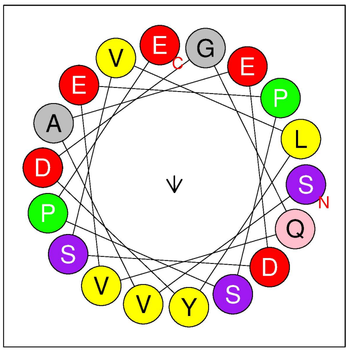

Go to screening | Manual mutation | GA mutation

---

$_{499}$**VAEDSVLYDGQVEPSPES**$_{516}$

Click to enlarge

**Physico-chemical properties**
**Hydrophobicity <H>**
0.237

**Polar residues + GLY**
**Polar residues + GLY (n / %)**
10 / 55.56

**Nonpolar residues**
**Nonpolar residues (n / %)**
8 / 44.44

**Hydrophobic moment <μH>** 0.123
**Net charge z** -5

**Uncharged residues + GLY** GLN 1, SER 3, GLY 1
**Charged residues** GLU 3, ASP 2,
**Hydrophobic face :** none

**Aromatic residues** TYR 1,
**Special residues** CYS 0, PRO 2

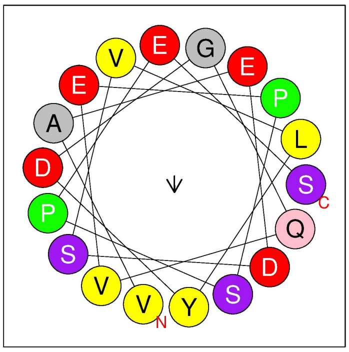


Go to screening | Manual mutation | GA mutation

---

## $_{500}$AEDSVLYDGQVEPSPESP$_{517}$

Click to enlarge

| Physico-chemical properties | Polar residues + GLY | Nonpolar residues |
|---|---|---|
| **Hydrophobicity <H>** | **Polar residues + GLY (n / %)** | **Nonpolar residues (n / %)** |
| 0.209 | 10 / 55.56 | 8 / 44.44 |
| **Hydrophobic moment <μH>** | **Uncharged residues + GLY** | **Aromatic residues** |
| 0.096 | GLN 1, SER 3, GLY 1 | TYR 1, |
| **Net charge z** | **Charged residues** | **Special residues** |
| -5 | GLU 3, ASP 2, | CYS 0, PRO 3 |
| | **Hydrophobic face :** none | |

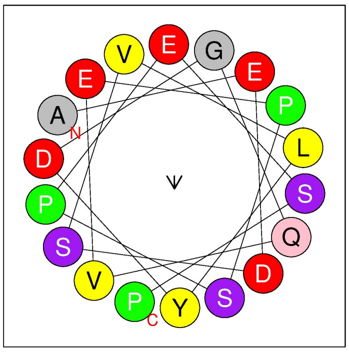


Go to screening | Manual mutation | GA mutation

---

## $_{501}$EDSVLYDGQVEPSPESPK$_{518}$

Click to enlarge

| Physico-chemical properties | Polar residues + GLY | Nonpolar residues |
|---|---|---|
| **Hydrophobicity <H>** | **Polar residues + GLY (n / %)** | **Nonpolar residues (n / %)** |
| 0.137 | 11 / 61.11 | 7 / 38.89 |
| **Hydrophobic moment <μH>** | **Uncharged residues + GLY** | **Aromatic residues** |
| 0.145 | GLN 1, SER 3, GLY 1 | TYR 1, |
| **Net charge z** | **Charged residues** | **Special residues** |
| -4 | LYS 1, GLU 3, ASP 2, | CYS 0, PRO 3 |
| | **Hydrophobic face :** none | |

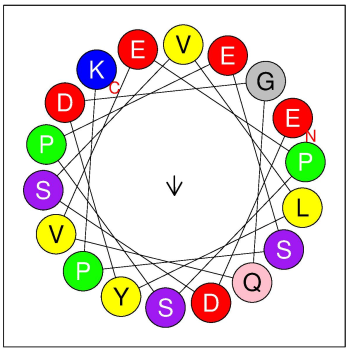


Go to screening | Manual mutation | GA mutation

---

## $_{502}$DSVLYDGQVEPSPESPKP$_{519}$

Click to enlarge

| Physico-chemical properties | Polar residues + GLY | Nonpolar residues |
|---|---|---|
| **Hydrophobicity <H>** | **Polar residues + GLY (n / %)** | **Nonpolar residues (n / %)** |
| 0.213 | 10 / 55.56 | 8 / 44.44 |
| **Hydrophobic moment <μH>** | **Uncharged residues + GLY** | **Aromatic residues** |
| 0.130 | GLN 1, SER 3, GLY 1 | TYR 1, |
| **Net charge z** | **Charged residues** | **Special residues** |
| -3 | LYS 1, GLU 2, ASP 2, | CYS 0, PRO 4 |
| | **Hydrophobic face :** none | |

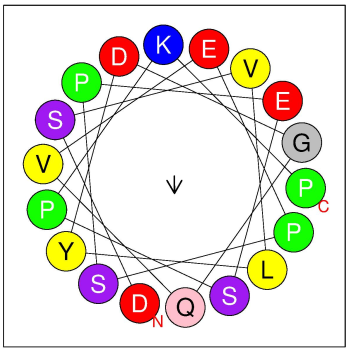


Go to screening | Manual mutation | GA mutation

---

## $_{503}$SVLYDGQVEPSPESPKPP$_{520}$

Click to enlarge

| Physico-chemical properties | Polar residues + GLY | Nonpolar residues |
|---|---|---|
| **Hydrophobicity <H>** | **Polar residues + GLY (n / %)** | **Nonpolar residues (n / %)** |
| 0.296 | 9 / 50.00 | 9 / 50.00 |

**Hydrophobic moment <μH>**
0.211
**Net charge z**
-2

**Uncharged residues + GLY**
GLN 1, SER 3, GLY 1
**Charged residues**
LYS 1, GLU 2, ASP 1,
**Hydrophobic face :** none

**Aromatic residues**
TYR 1,
**Special residues**
CYS 0, PRO 5

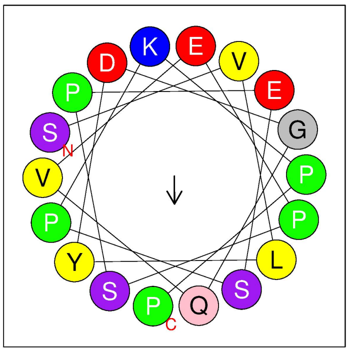


Go to screening | Manual mutation | GA mutation

---

$_{504}$**VLYDGQVEPSPESPKPPL**$_{521}$

Click to enlarge

| Physico-chemical properties | Polar residues + GLY | Nonpolar residues |
|---|---|---|
| **Hydrophobicity <H>** | **Polar residues + GLY (n / %)** | **Nonpolar residues (n / %)** |
| 0.392 | 8 / 44.44 | 10 / 55.56 |
| **Hydrophobic moment <μH>** | **Uncharged residues + GLY** | **Aromatic residues** |
| 0.201 | GLN 1, SER 2, GLY 1 | TYR 1, |
| **Net charge z** | **Charged residues** | **Special residues** |
| -2 | LYS 1, GLU 2, ASP 1, | CYS 0, PRO 5 |
| | **Hydrophobic face :** Y P V L P | |

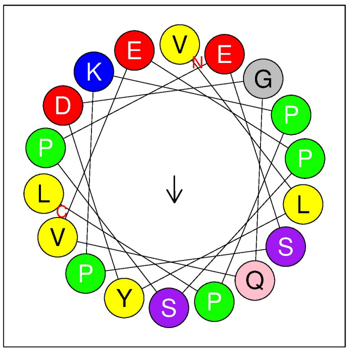


Go to screening | Manual mutation | GA mutation

---

$_{505}$**LYDGQVEPSPESPKPPLE**$_{522}$

Click to enlarge

| Physico-chemical properties | Polar residues + GLY | Nonpolar residues |
|---|---|---|
| **Hydrophobicity <H>** | **Polar residues + GLY (n / %)** | **Nonpolar residues (n / %)** |
| 0.289 | 9 / 50.00 | 9 / 50.00 |
| **Hydrophobic moment <μH>** | **Uncharged residues + GLY** | **Aromatic residues** |
| 0.304 | GLN 1, SER 2, GLY 1 | TYR 1, |
| **Net charge z** | **Charged residues** | **Special residues** |
| -3 | LYS 1, GLU 3, ASP 1, | CYS 0, PRO 5 |
| | **Hydrophobic face :** Y P V L P | |

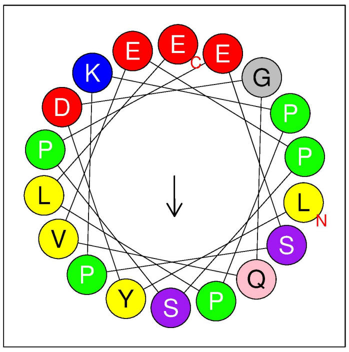


Go to screening | Manual mutation | GA mutation

---

$_{506}$**YDGQVEPSPESPKPPLEN**$_{523}$

Click to enlarge

| Physico-chemical properties | Polar residues + GLY | Nonpolar residues |
|---|---|---|
| **Hydrophobicity <H>** | **Polar residues + GLY (n / %)** | **Nonpolar residues (n / %)** |
| 0.161 | 10 / 55.56 | 8 / 44.44 |
| **Hydrophobic moment <μH>** | **Uncharged residues + GLY** | **Aromatic residues** |
| 0.306 | GLN 1, SER 2, ASN 1, GLY 1 | TYR 1, |
| **Net charge z** | **Charged residues** | **Special residues** |
| -3 | LYS 1, GLU 3, ASP 1, | CYS 0, PRO 5 |
| | **Hydrophobic face :** Y P V L P | |

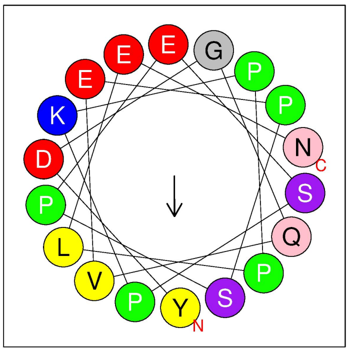


Go to screening | Manual mutation | GA mutation

---

$_{507}$**DGQVEPSPESPKPPLENG**$_{524}$

Click to enlarge

| Physico-chemical properties | Polar residues + GLY | Nonpolar residues |
|---|---|---|
| **Hydrophobicity <H>** | **Polar residues + GLY (n / %)** | **Nonpolar residues (n / %)** |
| 0.108 | 11 / 61.11 | 7 / 38.89 |

| | | | |
|---|---|---|---|
| **Hydrophobic moment <μH>** | **Uncharged residues + GLY** | **Aromatic residues** | 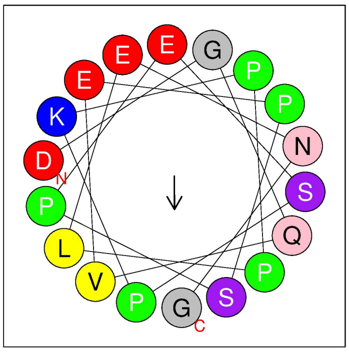 |
| 0.252 | GLN 1, SER 2, ASN 1, GLY 2 | | |
| **Net charge z** | **Charged residues** | **Special residues** | |
| -3 | LYS 1, GLU 3, ASP 1, | CYS 0, PRO 5 | |
| | **Hydrophobic face :** none | | |
| Go to screening | Manual mutation | GA mutation | |

---

$_{508}$**GQVEPSPESPKPPLENGQ**$_{525}$

Click to enlarge

| | | | |
|---|---|---|---|
| **Physico-chemical properties** | **Polar residues + GLY** | **Nonpolar residues** | 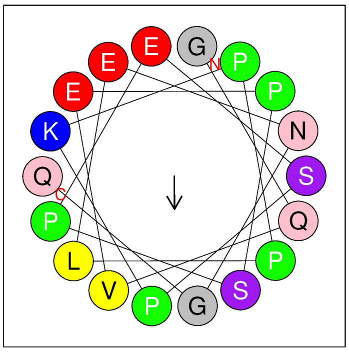 |
| **Hydrophobicity <H>** | **Polar residues + GLY (n / %)** | **Nonpolar residues (n / %)** | |
| 0.138 | 11 / 61.11 | 7 / 38.89 | |
| **Hydrophobic moment <μH>** | **Uncharged residues + GLY** | **Aromatic residues** | |
| 0.251 | GLN 2, SER 2, ASN 1, GLY 2 | | |
| **Net charge z** | **Charged residues** | **Special residues** | |
| -2 | LYS 1, GLU 3, | CYS 0, PRO 5 | |
| | **Hydrophobic face :** none | | |
| Go to screening | Manual mutation | GA mutation | |

---

$_{509}$**QVEPSPESPKPPLENGQV**$_{526}$

Click to enlarge

| | | | |
|---|---|---|---|
| **Physico-chemical properties** | **Polar residues + GLY** | **Nonpolar residues** | 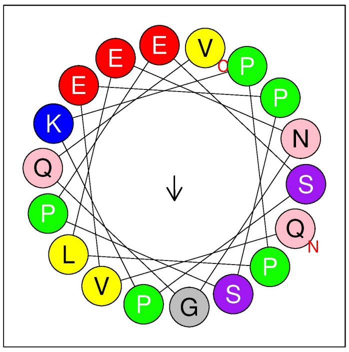 |
| **Hydrophobicity <H>** | **Polar residues + GLY (n / %)** | **Nonpolar residues (n / %)** | |
| 0.206 | 10 / 55.56 | 8 / 44.44 | |
| **Hydrophobic moment <μH>** | **Uncharged residues + GLY** | **Aromatic residues** | |
| 0.184 | GLN 2, SER 2, ASN 1, GLY 1 | | |
| **Net charge z** | **Charged residues** | **Special residues** | |
| -2 | LYS 1, GLU 3, | CYS 0, PRO 5 | |
| | **Hydrophobic face :** none | | |
| Go to screening | Manual mutation | GA mutation | |

---

$_{510}$**VEPSPESPKPPLENGQVG**$_{527}$

Click to enlarge

| | | | |
|---|---|---|---|
| **Physico-chemical properties** | **Polar residues + GLY** | **Nonpolar residues** | 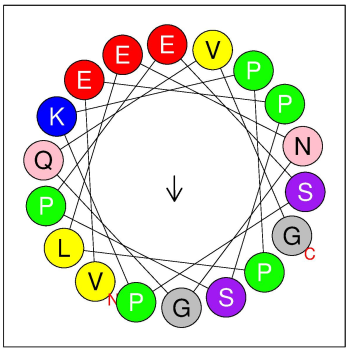 |
| **Hydrophobicity <H>** | **Polar residues + GLY (n / %)** | **Nonpolar residues (n / %)** | |
| 0.218 | 10 / 55.56 | 8 / 44.44 | |
| **Hydrophobic moment <μH>** | **Uncharged residues + GLY** | **Aromatic residues** | |
| 0.189 | GLN 1, SER 2, ASN 1, GLY 2 | | |
| **Net charge z** | **Charged residues** | **Special residues** | |
| -2 | LYS 1, GLU 3, | CYS 0, PRO 5 | |
| | **Hydrophobic face :** none | | |
| Go to screening | Manual mutation | GA mutation | |

---

$_{511}$**EPSPESPKPPLENGQVGL**$_{528}$

Click to enlarge

| | | |
|---|---|---|
| **Physico-chemical properties** | **Polar residues + GLY** | **Nonpolar residues** |
| **Hydrophobicity <H>** | **Polar residues + GLY (n / %)** | **Nonpolar residues (n / %)** |
| 0.245 | 10 / 55.56 | 8 / 44.44 |

**Hydrophobic moment <μH>**
0.211
**Net charge z**
-2

**Uncharged residues + GLY**
GLN 1, SER 2, ASN 1, GLY 2
**Charged residues**
LYS 1, GLU 3,
**Hydrophobic face :** none

**Aromatic residues**

**Special residues**
CYS 0, PRO 5

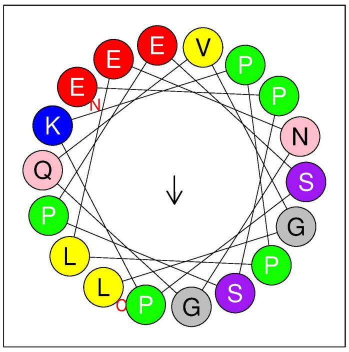

Go to screening | Manual mutation | GA mutation

---

**$_{512}$PSPESPKPPLENGQVGLQ$_{529}$**

Click to enlarge

**Physico-chemical properties**
**Hydrophobicity <H>**
0.268
**Hydrophobic moment <μH>**
0.196
**Net charge z**
-1

**Polar residues + GLY**
**Polar residues + GLY (n / %)**
10 / 55.56
**Uncharged residues + GLY**
GLN 2, SER 2, ASN 1, GLY 2
**Charged residues**
LYS 1, GLU 2,
**Hydrophobic face :** none

**Nonpolar residues**
**Nonpolar residues (n / %)**
8 / 44.44
**Aromatic residues**

**Special residues**
CYS 0, PRO 5

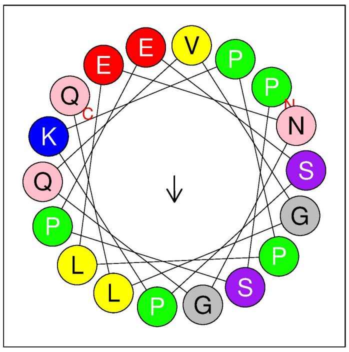

Go to screening | Manual mutation | GA mutation

---

**$_{513}$SPESPKPPLENGQVGLQE$_{530}$**

Click to enlarge

**Physico-chemical properties**
**Hydrophobicity <H>**
0.193
**Hydrophobic moment <μH>**
0.254
**Net charge z**
-2

**Polar residues + GLY**
**Polar residues + GLY (n / %)**
11 / 61.11
**Uncharged residues + GLY**
GLN 2, SER 2, ASN 1, GLY 2
**Charged residues**
LYS 1, GLU 3,
**Hydrophobic face :** none

**Nonpolar residues**
**Nonpolar residues (n / %)**
7 / 38.89
**Aromatic residues**

**Special residues**
CYS 0, PRO 4

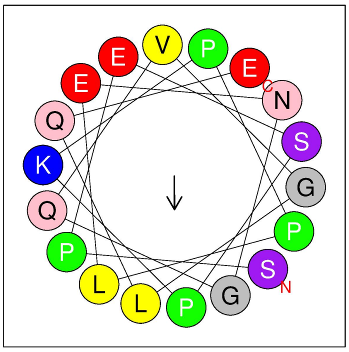

Go to screening | Manual mutation | GA mutation

---

**$_{514}$PESPKPPLENGQVGLQEK$_{531}$**

Click to enlarge

**Physico-chemical properties**
**Hydrophobicity <H>**
0.140
**Hydrophobic moment <μH>**
0.220
**Net charge z**
-1

**Polar residues + GLY**
**Polar residues + GLY (n / %)**
11 / 61.11
**Uncharged residues + GLY**
GLN 2, SER 1, ASN 1, GLY 2
**Charged residues**
LYS 2, GLU 3,
**Hydrophobic face :** none

**Nonpolar residues**
**Nonpolar residues (n / %)**
7 / 38.89
**Aromatic residues**

**Special residues**
CYS 0, PRO 4

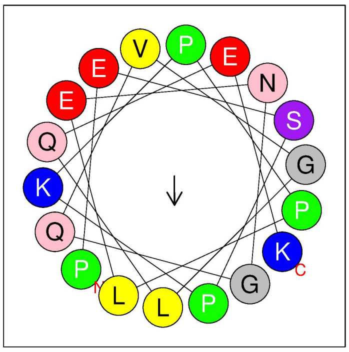

Go to screening | Manual mutation | GA mutation

---

**$_{515}$ESPKPPLENGQVGLQEKE$_{532}$**

Click to enlarge

**Physico-chemical properties**
**Hydrophobicity <H>**
0.064

**Polar residues + GLY**
**Polar residues + GLY (n / %)**
12 / 66.67

**Nonpolar residues**
**Nonpolar residues (n / %)**
6 / 33.33

| | | | |
|---|---|---|---|
| **Hydrophobic moment <μH>** | **Uncharged residues + GLY** | **Aromatic residues** | 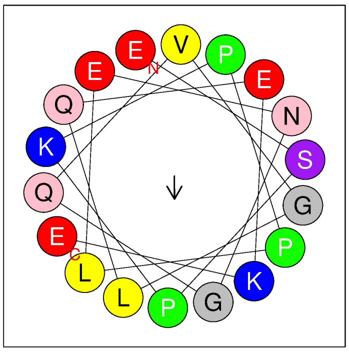 |
| 0.175 | GLN 2, SER 1, ASN 1, GLY 2 | | |
| **Net charge z** | **Charged residues** | **Special residues** | |
| -2 | LYS 2, GLU 4, | CYS 0, PRO 3 | |
| | **Hydrophobic face :** none | | |
| Go to screening | Manual mutation | GA mutation | |

---

$_{516}$**SPKPPLENGQVGLQEKED**$_{533}$

Click to enlarge

| | | | |
|---|---|---|---|
| **Physico-chemical properties** | **Polar residues + GLY** | **Nonpolar residues** | 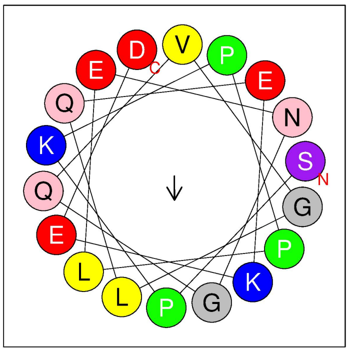 |
| **Hydrophobicity <H>** | **Polar residues + GLY (n / %)** | **Nonpolar residues (n / %)** | |
| 0.057 | 12 / 66.67 | 6 / 33.33 | |
| **Hydrophobic moment <μH>** | **Uncharged residues + GLY** | **Aromatic residues** | |
| 0.181 | GLN 2, SER 1, ASN 1, GLY 2 | | |
| **Net charge z** | **Charged residues** | **Special residues** | |
| -2 | LYS 2, GLU 3, ASP 1, | CYS 0, PRO 3 | |
| | **Hydrophobic face :** none | | |
| Go to screening | Manual mutation | GA mutation | |

---

$_{517}$**PKPPLENGQVGLQEKEDG**$_{534}$

Click to enlarge

| | | | |
|---|---|---|---|
| **Physico-chemical properties** | **Polar residues + GLY** | **Nonpolar residues** | 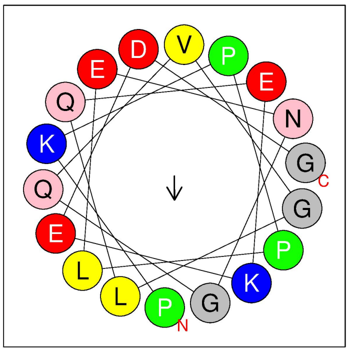 |
| **Hydrophobicity <H>** | **Polar residues + GLY (n / %)** | **Nonpolar residues (n / %)** | |
| 0.059 | 12 / 66.67 | 6 / 33.33 | |
| **Hydrophobic moment <μH>** | **Uncharged residues + GLY** | **Aromatic residues** | |
| 0.181 | GLN 2, ASN 1, GLY 3 | | |
| **Net charge z** | **Charged residues** | **Special residues** | |
| -2 | LYS 2, GLU 3, ASP 1, | CYS 0, PRO 3 | |
| | **Hydrophobic face :** none | | |
| Go to screening | Manual mutation | GA mutation | |

---

$_{518}$**KPPLENGQVGLQEKEDGQ**$_{535}$

Click to enlarge

| | | | |
|---|---|---|---|
| **Physico-chemical properties** | **Polar residues + GLY** | **Nonpolar residues** | 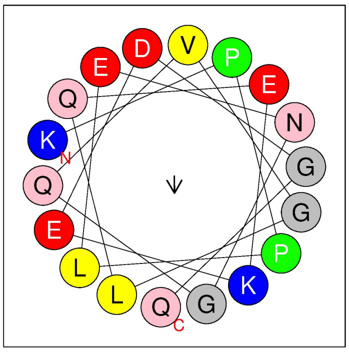 |
| **Hydrophobicity <H>** | **Polar residues + GLY (n / %)** | **Nonpolar residues (n / %)** | |
| 0.007 | 13 / 72.22 | 5 / 27.78 | |
| **Hydrophobic moment <μH>** | **Uncharged residues + GLY** | **Aromatic residues** | |
| 0.129 | GLN 3, ASN 1, GLY 3 | | |
| **Net charge z** | **Charged residues** | **Special residues** | |
| -2 | LYS 2, GLU 3, ASP 1, | CYS 0, PRO 2 | |
| | **Hydrophobic face :** none | | |
| Go to screening | Manual mutation | GA mutation | |

---

$_{519}$**PPLENGQVGLQEKEDGQP**$_{536}$

Click to enlarge

| | | |
|---|---|---|
| **Physico-chemical properties** | **Polar residues + GLY** | **Nonpolar residues** |
| **Hydrophobicity <H>** | **Polar residues + GLY (n / %)** | **Nonpolar residues (n / %)** |
| 0.102 | 12 / 66.67 | 6 / 33.33 |

**Hydrophobic moment <μH>**
0.138
**Net charge z**
-3

**Uncharged residues + GLY**
GLN 3, ASN 1, GLY 3
**Charged residues**
LYS 1, GLU 3, ASP 1,
**Hydrophobic face :** none

**Aromatic residues**

**Special residues**
CYS 0, PRO 3

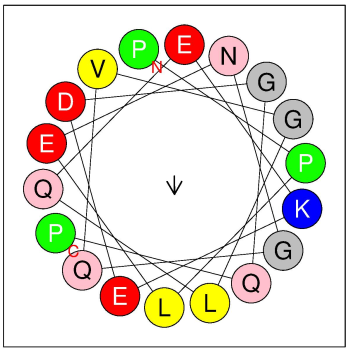


Go to screening | Manual mutation | GA mutation

---

### $_{520}$PLENGQVGLQEKEDGQPI$_{537}$

Click to enlarge

**Physico-chemical properties**
**Hydrophobicity <H>**
0.162
**Hydrophobic moment <μH>**
0.082
**Net charge z**
-3

**Polar residues + GLY**
**Polar residues + GLY (n / %)**
12 / 66.67
**Uncharged residues + GLY**
GLN 3, ASN 1, GLY 3
**Charged residues**
LYS 1, GLU 3, ASP 1,
**Hydrophobic face :** none

**Nonpolar residues**
**Nonpolar residues (n / %)**
6 / 33.33
**Aromatic residues**

**Special residues**
CYS 0, PRO 2

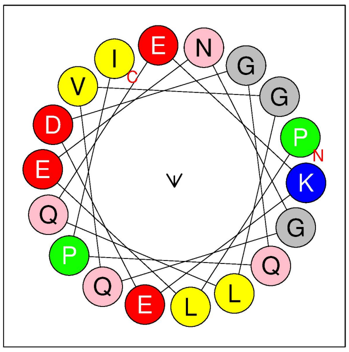


Go to screening | Manual mutation | GA mutation

---

### $_{521}$LENGQVGLQEKEDGQPID$_{538}$

Click to enlarge

**Physico-chemical properties**
**Hydrophobicity <H>**
0.079
**Hydrophobic moment <μH>**
0.132
**Net charge z**
-4

**Polar residues + GLY**
**Polar residues + GLY (n / %)**
13 / 72.22
**Uncharged residues + GLY**
GLN 3, ASN 1, GLY 3
**Charged residues**
LYS 1, GLU 3, ASP 2,
**Hydrophobic face :** none

**Nonpolar residues**
**Nonpolar residues (n / %)**
5 / 27.78
**Aromatic residues**

**Special residues**
CYS 0, PRO 1

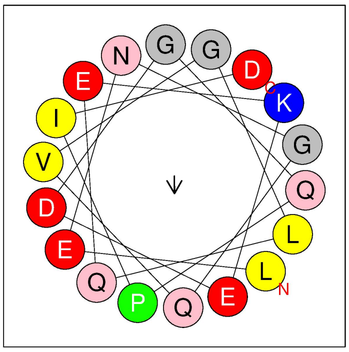


Go to screening | Manual mutation | GA mutation

---

### $_{522}$ENGQVGLQEKEDGQPIDQ$_{539}$

Click to enlarge

**Physico-chemical properties**
**Hydrophobicity <H>**
-0.027
**Hydrophobic moment <μH>**
0.092
**Net charge z**
-4

**Polar residues + GLY**
**Polar residues + GLY (n / %)**
14 / 77.78
**Uncharged residues + GLY**
GLN 4, ASN 1, GLY 3
**Charged residues**
LYS 1, GLU 3, ASP 2,
**Hydrophobic face :** none

**Nonpolar residues**
**Nonpolar residues (n / %)**
4 / 22.22
**Aromatic residues**

**Special residues**
CYS 0, PRO 1

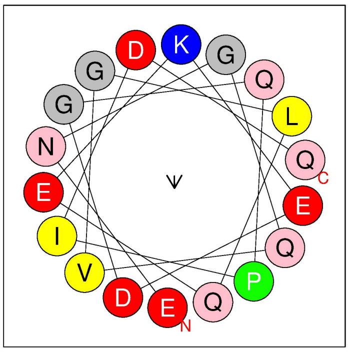


Go to screening | Manual mutation | GA mutation

---

### $_{523}$NGQVGLQEKEDGQPIDQQ$_{540}$

Click to enlarge

**Physico-chemical properties**
**Hydrophobicity <H>**
-0.004

**Polar residues + GLY**
**Polar residues + GLY (n / %)**
14 / 77.78

**Nonpolar residues**
**Nonpolar residues (n / %)**
4 / 22.22

| | | |
|---|---|---|
| **Hydrophobic moment <μH>** | **Uncharged residues + GLY** | **Aromatic residues** |
| 0.116 | GLN 5, ASN 1, GLY 3 | |
| **Net charge z** | **Charged residues** | **Special residues** |
| -3 | LYS 1, GLU 2, ASP 2, | CYS 0, PRO 1 |
| | **Hydrophobic face :** none | |
| Go to screening | Manual mutation | GA mutation |

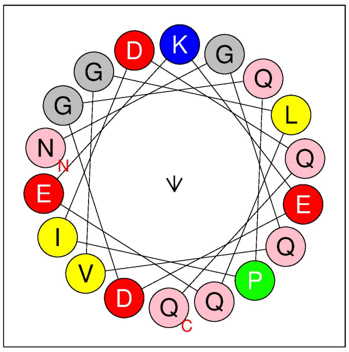


---

**$_{524}$GQVGLQEKEDGQPIDQQP$_{541}$**

Click to enlarge

| | | |
|---|---|---|
| **Physico-chemical properties** | **Polar residues + GLY** | **Nonpolar residues** |
| **Hydrophobicity <H>** | **Polar residues + GLY (n / %)** | **Nonpolar residues (n / %)** |
| 0.069 | 13 / 72.22 | 5 / 27.78 |
| **Hydrophobic moment <μH>** | **Uncharged residues + GLY** | **Aromatic residues** |
| 0.123 | GLN 5, GLY 3 | |
| **Net charge z** | **Charged residues** | **Special residues** |
| -3 | LYS 1, GLU 2, ASP 2, | CYS 0, PRO 2 |
| | **Hydrophobic face :** none | |
| Go to screening | Manual mutation | GA mutation |

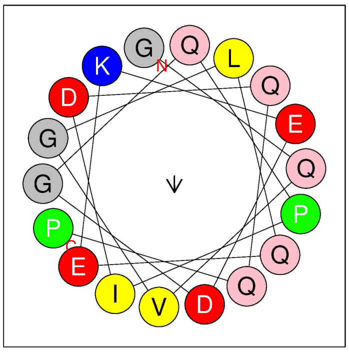


---

**$_{525}$QVGLQEKEDGQPIDQQPI$_{542}$**

Click to enlarge

| | | |
|---|---|---|
| **Physico-chemical properties** | **Polar residues + GLY** | **Nonpolar residues** |
| **Hydrophobicity <H>** | **Polar residues + GLY (n / %)** | **Nonpolar residues (n / %)** |
| 0.169 | 12 / 66.67 | 6 / 33.33 |
| **Hydrophobic moment <μH>** | **Uncharged residues + GLY** | **Aromatic residues** |
| 0.034 | GLN 5, GLY 2 | |
| **Net charge z** | **Charged residues** | **Special residues** |
| -3 | LYS 1, GLU 2, ASP 2, | CYS 0, PRO 2 |
| | **Hydrophobic face :** none | |
| Go to screening | Manual mutation | GA mutation |

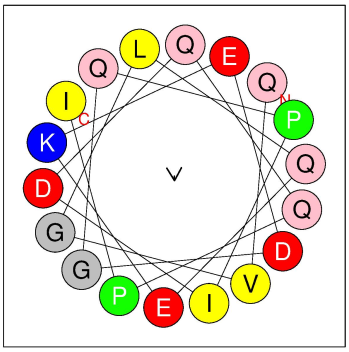


---

**$_{526}$VGLQEKEDGQPIDQQPID$_{543}$**

Click to enlarge

| | | |
|---|---|---|
| **Physico-chemical properties** | **Polar residues + GLY** | **Nonpolar residues** |
| **Hydrophobicity <H>** | **Polar residues + GLY (n / %)** | **Nonpolar residues (n / %)** |
| 0.139 | 12 / 66.67 | 6 / 33.33 |
| **Hydrophobic moment <μH>** | **Uncharged residues + GLY** | **Aromatic residues** |
| 0.060 | GLN 4, GLY 2 | |
| **Net charge z** | **Charged residues** | **Special residues** |
| -4 | LYS 1, GLU 2, ASP 3, | CYS 0, PRO 2 |
| | **Hydrophobic face :** none | |
| Go to screening | Manual mutation | GA mutation |

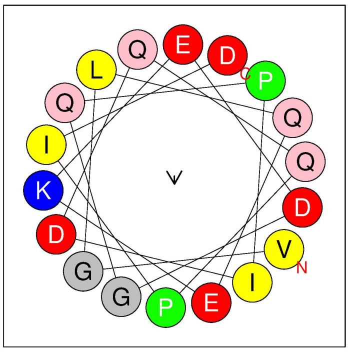


---

**$_{527}$GLQEKEDGQPIDQQPIDK$_{544}$**

Click to enlarge

| | | |
|---|---|---|
| **Physico-chemical properties** | **Polar residues + GLY** | **Nonpolar residues** |
| **Hydrophobicity <H>** | **Polar residues + GLY (n / %)** | **Nonpolar residues (n / %)** |
| 0.016 | 13 / 72.22 | 5 / 27.78 |

| | | |
|---|---|---|
| **Hydrophobic moment <μH>** | **Uncharged residues + GLY** | **Aromatic residues** |
| 0.102 | GLN 4, GLY 2 | |
| **Net charge z** | **Charged residues** | **Special residues** |
| -3 | LYS 2, GLU 2, ASP 3, | CYS 0, PRO 2 |
| | **Hydrophobic face :** none | |
| Go to screening | Manual mutation | GA mutation |

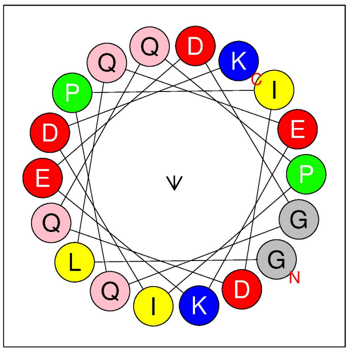

---

$_{528}$**LQEKEDGQPIDQQPIDKE**$_{545}$

Click to enlarge

| | | |
|---|---|---|
| **Physico-chemical properties** | **Polar residues + GLY** | **Nonpolar residues** |
| **Hydrophobicity <H>** | **Polar residues + GLY (n / %)** | **Nonpolar residues (n / %)** |
| -0.019 | 13 / 72.22 | 5 / 27.78 |
| **Hydrophobic moment <μH>** | **Uncharged residues + GLY** | **Aromatic residues** |
| 0.085 | GLN 4, GLY 1 | |
| **Net charge z** | **Charged residues** | **Special residues** |
| -4 | LYS 2, GLU 3, ASP 3, | CYS 0, PRO 2 |
| | **Hydrophobic face :** none | |
| Go to screening | Manual mutation | GA mutation |

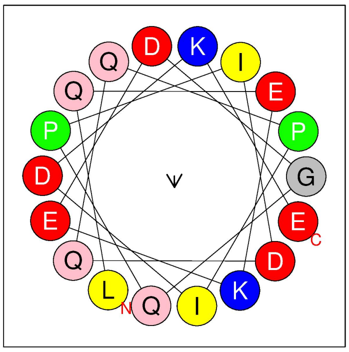

---

$_{529}$**QEKEDGQPIDQQPIDKEI**$_{546}$

Click to enlarge

| | | |
|---|---|---|
| **Physico-chemical properties** | **Polar residues + GLY** | **Nonpolar residues** |
| **Hydrophobicity <H>** | **Polar residues + GLY (n / %)** | **Nonpolar residues (n / %)** |
| -0.014 | 13 / 72.22 | 5 / 27.78 |
| **Hydrophobic moment <μH>** | **Uncharged residues + GLY** | **Aromatic residues** |
| 0.089 | GLN 4, GLY 1 | |
| **Net charge z** | **Charged residues** | **Special residues** |
| -4 | LYS 2, GLU 3, ASP 3, | CYS 0, PRO 2 |
| | **Hydrophobic face :** none | |
| Go to screening | Manual mutation | GA mutation |

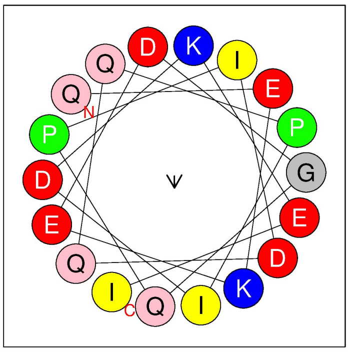

---

$_{530}$**EKEDGQPIDQQPIDKEIE**$_{547}$

Click to enlarge

| | | |
|---|---|---|
| **Physico-chemical properties** | **Polar residues + GLY** | **Nonpolar residues** |
| **Hydrophobicity <H>** | **Polar residues + GLY (n / %)** | **Nonpolar residues (n / %)** |
| -0.037 | 13 / 72.22 | 5 / 27.78 |
| **Hydrophobic moment <μH>** | **Uncharged residues + GLY** | **Aromatic residues** |
| 0.105 | GLN 3, GLY 1 | |
| **Net charge z** | **Charged residues** | **Special residues** |
| -5 | LYS 2, GLU 4, ASP 3, | CYS 0, PRO 2 |
| | **Hydrophobic face :** none | |
| Go to screening | Manual mutation | GA mutation |

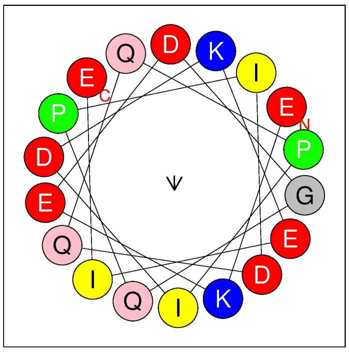

---

$_{531}$**KEDGQPIDQQPIDKEIEP**$_{548}$

Click to enlarge

| | | |
|---|---|---|
| **Physico-chemical properties** | **Polar residues + GLY** | **Nonpolar residues** |
| **Hydrophobicity <H>** | **Polar residues + GLY (n / %)** | **Nonpolar residues (n / %)** |
| 0.038 | 12 / 66.67 | 6 / 33.33 |

**Hydrophobic moment <μH>**
0.092
**Net charge z**
-4

**Uncharged residues + GLY**
GLN 3, GLY 1
**Charged residues**
LYS 2, GLU 3, ASP 3,
**Hydrophobic face :** none

**Aromatic residues**

**Special residues**
CYS 0, PRO 3

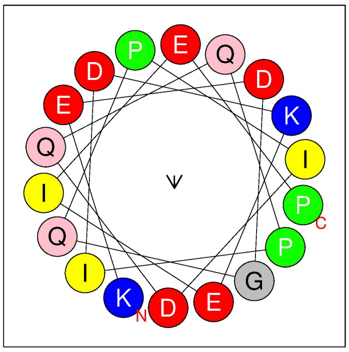

Go to screening | Manual mutation | GA mutation

---

## $_{532}$EDGQPIDQQPIDKEIEPD$_{549}$

Click to enlarge

**Physico-chemical properties**
**Hydrophobicity <H>**
0.051
**Hydrophobic moment <μH>**
0.103
**Net charge z**
-6

**Polar residues + GLY**
**Polar residues + GLY (n / %)**
12 / 66.67
**Uncharged residues + GLY**
GLN 3, GLY 1
**Charged residues**
LYS 1, GLU 3, ASP 4,
**Hydrophobic face :** none

**Nonpolar residues**
**Nonpolar residues (n / %)**
6 / 33.33
**Aromatic residues**

**Special residues**
CYS 0, PRO 3

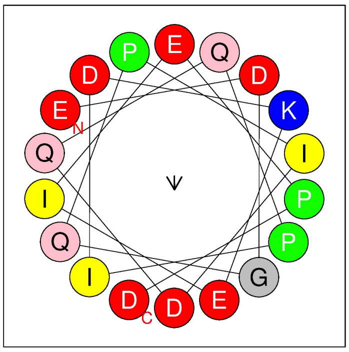

Go to screening | Manual mutation | GA mutation

---

## $_{533}$DGQPIDQQPIDKEIEPDG$_{550}$

Click to enlarge

**Physico-chemical properties**
**Hydrophobicity <H>**
0.086
**Hydrophobic moment <μH>**
0.091
**Net charge z**
-5

**Polar residues + GLY**
**Polar residues + GLY (n / %)**
12 / 66.67
**Uncharged residues + GLY**
GLN 3, GLY 2
**Charged residues**
LYS 1, GLU 2, ASP 4,
**Hydrophobic face :** none

**Nonpolar residues**
**Nonpolar residues (n / %)**
6 / 33.33
**Aromatic residues**

**Special residues**
CYS 0, PRO 3

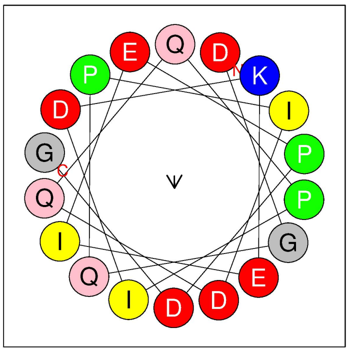

Go to screening | Manual mutation | GA mutation

---

## $_{534}$GQPIDQQPIDKEIEPDGA$_{551}$

Click to enlarge

**Physico-chemical properties**
**Hydrophobicity <H>**
0.146
**Hydrophobic moment <μH>**
0.040
**Net charge z**
-4

**Polar residues + GLY**
**Polar residues + GLY (n / %)**
11 / 61.11
**Uncharged residues + GLY**
GLN 3, GLY 2
**Charged residues**
LYS 1, GLU 2, ASP 3,
**Hydrophobic face :** none

**Nonpolar residues**
**Nonpolar residues (n / %)**
7 / 38.89
**Aromatic residues**

**Special residues**
CYS 0, PRO 3

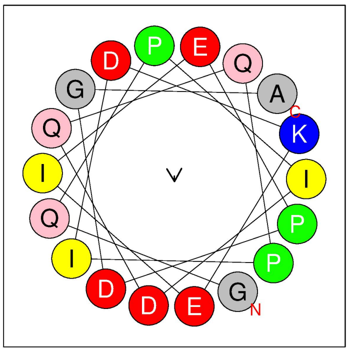

Go to screening | Manual mutation | GA mutation

---

## $_{535}$QPIDQQPIDKEIEPDGAE$_{552}$

Click to enlarge

**Physico-chemical properties**
**Hydrophobicity <H>**
0.111

**Polar residues + GLY**
**Polar residues + GLY (n / %)**
11 / 61.11

**Nonpolar residues**
**Nonpolar residues (n / %)**
7 / 38.89

| | | |
|---|---|---|
| **Hydrophobic moment <μH>** | **Uncharged residues + GLY** | **Aromatic residues** |
| 0.019 | GLN 3, GLY 1 | |
| **Net charge z** | **Charged residues** | **Special residues** |
| -5 | LYS 1, GLU 3, ASP 3, | CYS 0, PRO 3 |
| | **Hydrophobic face :** none | |
| Go to screening | Manual mutation | GA mutation |

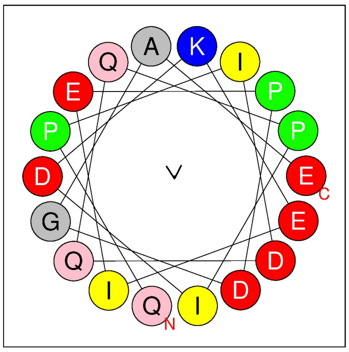

---

$_{536}$**PIDQQPIDKEIEPDGAEL**$_{553}$

Click to enlarge

| **Physico-chemical properties** | **Polar residues + GLY** | **Nonpolar residues** |
|---|---|---|
| **Hydrophobicity <H>** | **Polar residues + GLY (n / %)** | **Nonpolar residues (n / %)** |
| 0.217 | 10 / 55.56 | 8 / 44.44 |
| **Hydrophobic moment <μH>** | **Uncharged residues + GLY** | **Aromatic residues** |
| 0.126 | GLN 2, GLY 1 | |
| **Net charge z** | **Charged residues** | **Special residues** |
| -5 | LYS 1, GLU 3, ASP 3, | CYS 0, PRO 3 |
| | **Hydrophobic face :** none | |
| Go to screening | Manual mutation | GA mutation |

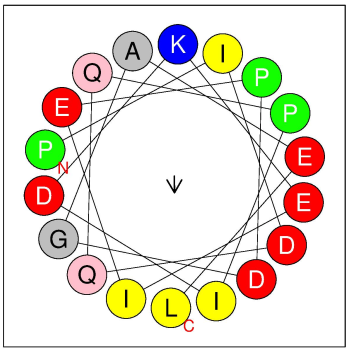

---

$_{537}$**IDQQPIDKEIEPDGAELE**$_{554}$

Click to enlarge

| **Physico-chemical properties** | **Polar residues + GLY** | **Nonpolar residues** |
|---|---|---|
| **Hydrophobicity <H>** | **Polar residues + GLY (n / %)** | **Nonpolar residues (n / %)** |
| 0.142 | 11 / 61.11 | 7 / 38.89 |
| **Hydrophobic moment <μH>** | **Uncharged residues + GLY** | **Aromatic residues** |
| 0.159 | GLN 2, GLY 1 | |
| **Net charge z** | **Charged residues** | **Special residues** |
| -6 | LYS 1, GLU 4, ASP 3, | CYS 0, PRO 2 |
| | **Hydrophobic face :** none | |
| Go to screening | Manual mutation | GA mutation |

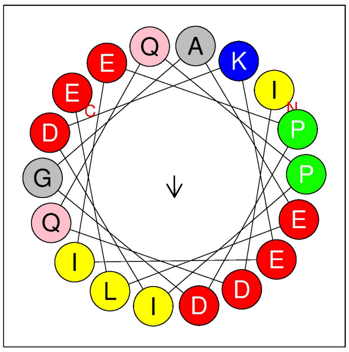

---

$_{538}$**DQQPIDKEIEPDGAELEG**$_{555}$

Click to enlarge

| **Physico-chemical properties** | **Polar residues + GLY** | **Nonpolar residues** |
|---|---|---|
| **Hydrophobicity <H>** | **Polar residues + GLY (n / %)** | **Nonpolar residues (n / %)** |
| 0.042 | 12 / 66.67 | 6 / 33.33 |
| **Hydrophobic moment <μH>** | **Uncharged residues + GLY** | **Aromatic residues** |
| 0.237 | GLN 2, GLY 2 | |
| **Net charge z** | **Charged residues** | **Special residues** |
| -6 | LYS 1, GLU 4, ASP 3, | CYS 0, PRO 2 |
| | **Hydrophobic face :** none | |
| Go to screening | Manual mutation | GA mutation |

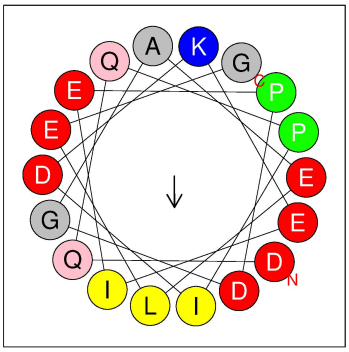

---

$_{539}$**QQPIDKEIEPDGAELEGP**$_{556}$

Click to enlarge

| **Physico-chemical properties** | **Polar residues + GLY** | **Nonpolar residues** |
|---|---|---|
| **Hydrophobicity <H>** | **Polar residues + GLY (n / %)** | **Nonpolar residues (n / %)** |
| 0.124 | 11 / 61.11 | 7 / 38.89 |

| | | |
|---|---|---|
| **Hydrophobic moment <μH>** | **Uncharged residues + GLY** | **Aromatic residues** |
| 0.298 | GLN 2, GLY 2 | |
| **Net charge z** | **Charged residues** | **Special residues** |
| -5 | LYS 1, GLU 4, ASP 2, | CYS 0, PRO 3 |
| | **Hydrophobic face :** none | |

Go to screening | Manual mutation | GA mutation

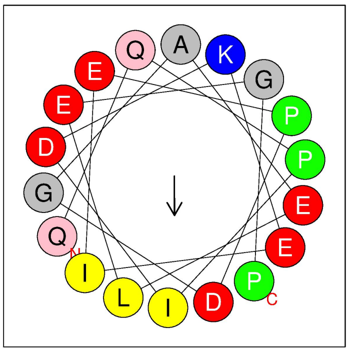


---

$_{540}$**QPIDKEIEPDGAELEGPE**$_{557}$

Click to enlarge

| **Physico-chemical properties** | **Polar residues + GLY** | **Nonpolar residues** |
|---|---|---|
| **Hydrophobicity <H>** | **Polar residues + GLY (n / %)** | **Nonpolar residues (n / %)** |
| 0.101 | 11 / 61.11 | 7 / 38.89 |
| **Hydrophobic moment <μH>** | **Uncharged residues + GLY** | **Aromatic residues** |
| 0.288 | GLN 1, GLY 2 | |
| **Net charge z** | **Charged residues** | **Special residues** |
| -6 | LYS 1, GLU 5, ASP 2, | CYS 0, PRO 3 |
| | **Hydrophobic face :** none | |

Go to screening | Manual mutation | GA mutation

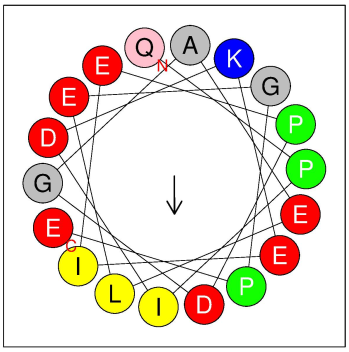


---

$_{541}$**PIDKEIEPDGAELEGPEE**$_{558}$

Click to enlarge

| **Physico-chemical properties** | **Polar residues + GLY** | **Nonpolar residues** |
|---|---|---|
| **Hydrophobicity <H>** | **Polar residues + GLY (n / %)** | **Nonpolar residues (n / %)** |
| 0.078 | 11 / 61.11 | 7 / 38.89 |
| **Hydrophobic moment <μH>** | **Uncharged residues + GLY** | **Aromatic residues** |
| 0.310 | GLY 2 | |
| **Net charge z** | **Charged residues** | **Special residues** |
| -7 | LYS 1, GLU 6, ASP 2, | CYS 0, PRO 3 |
| | **Hydrophobic face :** none | |

Go to screening | Manual mutation | GA mutation

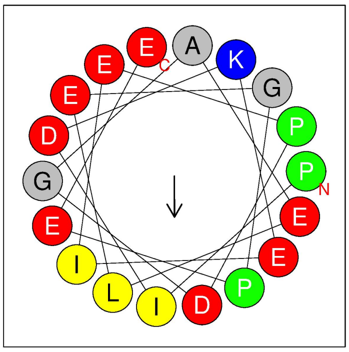


---

$_{542}$**IDKEIEPDGAELEGPEEK**$_{559}$

Click to enlarge

| **Physico-chemical properties** | **Polar residues + GLY** | **Nonpolar residues** |
|---|---|---|
| **Hydrophobicity <H>** | **Polar residues + GLY (n / %)** | **Nonpolar residues (n / %)** |
| -0.017 | 12 / 66.67 | 6 / 33.33 |
| **Hydrophobic moment <μH>** | **Uncharged residues + GLY** | **Aromatic residues** |
| 0.328 | GLY 2 | |
| **Net charge z** | **Charged residues** | **Special residues** |
| -6 | LYS 2, GLU 6, ASP 2, | CYS 0, PRO 2 |
| | **Hydrophobic face :** none | |

Go to screening | Manual mutation | GA mutation

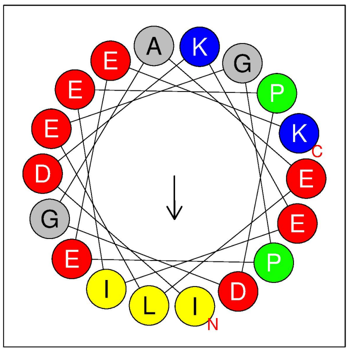


---

$_{543}$**DKEIEPDGAELEGPEEKR**$_{560}$

Click to enlarge

| **Physico-chemical properties** | **Polar residues + GLY** | **Nonpolar residues** |
|---|---|---|
| **Hydrophobicity <H>** | **Polar residues + GLY (n / %)** | **Nonpolar residues (n / %)** |
| -0.173 | 13 / 72.22 | 5 / 27.78 |

**Hydrophobic moment <μH>**
0.175
**Net charge z**
-5

**Uncharged residues + GLY**
GLY 2
**Charged residues**
LYS 2, ARG 1, GLU 6, ASP 2,
**Hydrophobic face :** none

**Aromatic residues**

**Special residues**
CYS 0, PRO 2

Go to screening    Manual mutation    GA mutation

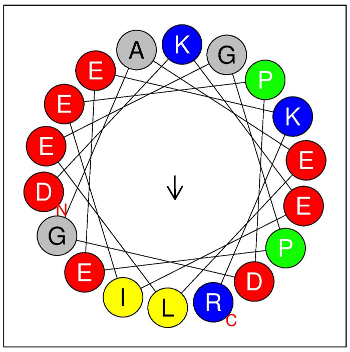


---

$_{544}$**KEIEPDGAELEGPEEKRE**$_{561}$

Click to enlarge

**Physico-chemical properties**
**Hydrophobicity <H>**
-0.166
**Hydrophobic moment <μH>**
0.176
**Net charge z**
-5

**Polar residues + GLY**
**Polar residues + GLY (n / %)**
13 / 72.22
**Uncharged residues + GLY**
GLY 2
**Charged residues**
LYS 2, ARG 1, GLU 7, ASP 1,
**Hydrophobic face :** none

**Nonpolar residues**
**Nonpolar residues (n / %)**
5 / 27.78
**Aromatic residues**

**Special residues**
CYS 0, PRO 2

Go to screening    Manual mutation    GA mutation

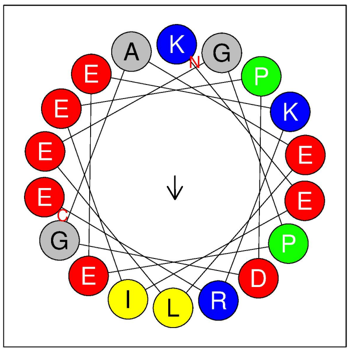


---

$_{545}$**EIEPDGAELEGPEEKREG**$_{562}$

Click to enlarge

**Physico-chemical properties**
**Hydrophobicity <H>**
-0.111
**Hydrophobic moment <μH>**
0.121
**Net charge z**
-6

**Polar residues + GLY**
**Polar residues + GLY (n / %)**
13 / 72.22
**Uncharged residues + GLY**
GLY 3
**Charged residues**
LYS 1, ARG 1, GLU 7, ASP 1,
**Hydrophobic face :** none

**Nonpolar residues**
**Nonpolar residues (n / %)**
5 / 27.78
**Aromatic residues**

**Special residues**
CYS 0, PRO 2

Go to screening    Manual mutation    GA mutation

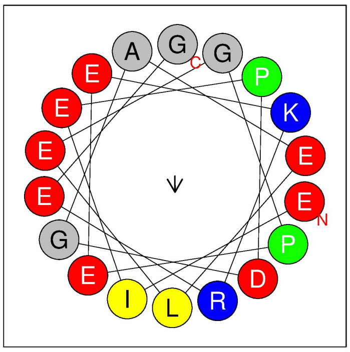


---

$_{546}$**IEPDGAELEGPEEKREGE**$_{563}$

Click to enlarge

**Physico-chemical properties**
**Hydrophobicity <H>**
-0.111
**Hydrophobic moment <μH>**
0.121
**Net charge z**
-6

**Polar residues + GLY**
**Polar residues + GLY (n / %)**
13 / 72.22
**Uncharged residues + GLY**
GLY 3
**Charged residues**
LYS 1, ARG 1, GLU 7, ASP 1,
**Hydrophobic face :** none

**Nonpolar residues**
**Nonpolar residues (n / %)**
5 / 27.78
**Aromatic residues**

**Special residues**
CYS 0, PRO 2

Go to screening    Manual mutation    GA mutation

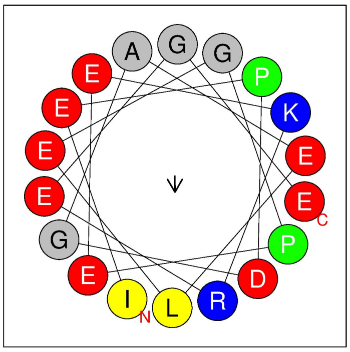


---

$_{547}$**EPDGAELEGPEEKREGEE**$_{564}$

Click to enlarge

**Physico-chemical properties**
**Hydrophobicity <H>**
-0.247

**Polar residues + GLY**
**Polar residues + GLY (n / %)**
14 / 77.78

**Nonpolar residues**
**Nonpolar residues (n / %)**
4 / 22.22

| | | |
|---|---|---|
| **Hydrophobic moment <μH>** | **Uncharged residues + GLY** | **Aromatic residues** |
| 0.049 | GLY 3 | |
| **Net charge z** | **Charged residues** | **Special residues** |
| -7 | LYS 1, ARG 1, GLU 8, ASP 1, | CYS 0, PRO 2 |
| | **Hydrophobic face :** none | |
| Go to screening | Manual mutation | GA mutation |

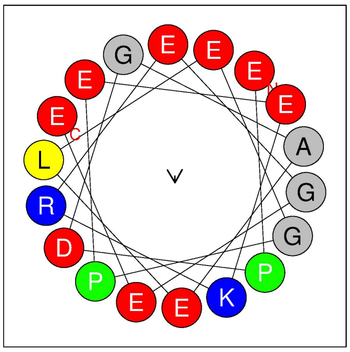

---

$_{548}$**PDGAELEGPEEKREGEER**$_{565}$

Click to enlarge

| | | |
|---|---|---|
| **Physico-chemical properties** | **Polar residues + GLY** | **Nonpolar residues** |
| **Hydrophobicity <H>** | **Polar residues + GLY (n / %)** | **Nonpolar residues (n / %)** |
| -0.267 | 14 / 77.78 | 4 / 22.22 |
| **Hydrophobic moment <μH>** | **Uncharged residues + GLY** | **Aromatic residues** |
| 0.067 | GLY 3 | |
| **Net charge z** | **Charged residues** | **Special residues** |
| -5 | LYS 1, ARG 2, GLU 7, ASP 1, | CYS 0, PRO 2 |
| | **Hydrophobic face :** none | |
| Go to screening | Manual mutation | GA mutation |

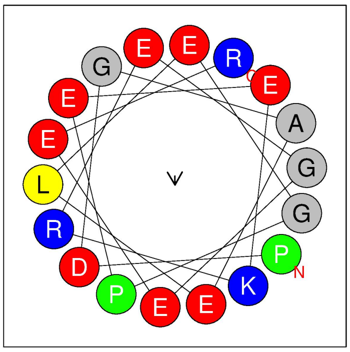

---

$_{549}$**DGAELEGPEEKREGEERD**$_{566}$

Click to enlarge

| | | |
|---|---|---|
| **Physico-chemical properties** | **Polar residues + GLY** | **Nonpolar residues** |
| **Hydrophobicity <H>** | **Polar residues + GLY (n / %)** | **Nonpolar residues (n / %)** |
| -0.350 | 15 / 83.33 | 3 / 16.67 |
| **Hydrophobic moment <μH>** | **Uncharged residues + GLY** | **Aromatic residues** |
| 0.069 | GLY 3 | |
| **Net charge z** | **Charged residues** | **Special residues** |
| -6 | LYS 1, ARG 2, GLU 7, ASP 2, | CYS 0, PRO 1 |
| | **Hydrophobic face :** none | |
| Go to screening | Manual mutation | GA mutation |

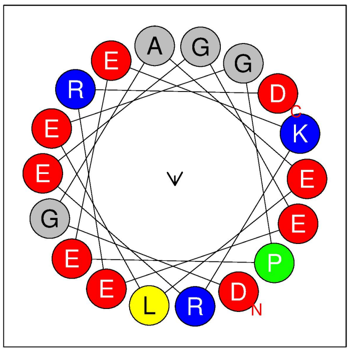

---

$_{550}$**GAELEGPEEKREGEERDE**$_{567}$

Click to enlarge

| | | |
|---|---|---|
| **Physico-chemical properties** | **Polar residues + GLY** | **Nonpolar residues** |
| **Hydrophobicity <H>** | **Polar residues + GLY (n / %)** | **Nonpolar residues (n / %)** |
| -0.343 | 15 / 83.33 | 3 / 16.67 |
| **Hydrophobic moment <μH>** | **Uncharged residues + GLY** | **Aromatic residues** |
| 0.075 | GLY 3 | |
| **Net charge z** | **Charged residues** | **Special residues** |
| -6 | LYS 1, ARG 2, GLU 8, ASP 1, | CYS 0, PRO 1 |
| | **Hydrophobic face :** none | |
| Go to screening | Manual mutation | GA mutation |

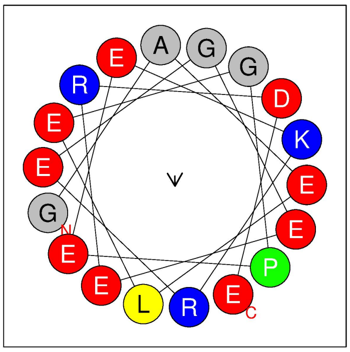

---

$_{551}$**AELEGPEEKREGEERDEE**$_{568}$

Click to enlarge

| | | |
|---|---|---|
| **Physico-chemical properties** | **Polar residues + GLY** | **Nonpolar residues** |
| **Hydrophobicity <H>** | **Polar residues + GLY (n / %)** | **Nonpolar residues (n / %)** |
| -0.378 | 15 / 83.33 | 3 / 16.67 |

**Hydrophobic moment <μH>**
0.074
**Net charge z**
-7

**Uncharged residues + GLY**
GLY 2
**Charged residues**
LYS 1, ARG 2, GLU 9, ASP 1,
**Hydrophobic face :** none

**Aromatic residues**

**Special residues**
CYS 0, PRO 1

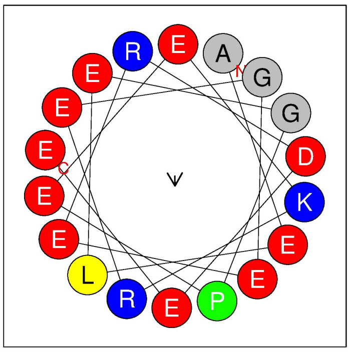

Go to screening | Manual mutation | GA mutation

---

$_{552}$**ELEGPEEKREGEERDEES**$_{569}$

Click to enlarge

**Physico-chemical properties**
**Hydrophobicity <H>**
-0.398
**Hydrophobic moment <μH>**
0.092
**Net charge z**
-7

**Polar residues + GLY**
**Polar residues + GLY (n / %)**
16 / 88.89
**Uncharged residues + GLY**
SER 1, GLY 2
**Charged residues**
LYS 1, ARG 2, GLU 9, ASP 1,
**Hydrophobic face :** none

**Nonpolar residues**
**Nonpolar residues (n / %)**
2 / 11.11
**Aromatic residues**

**Special residues**
CYS 0, PRO 1

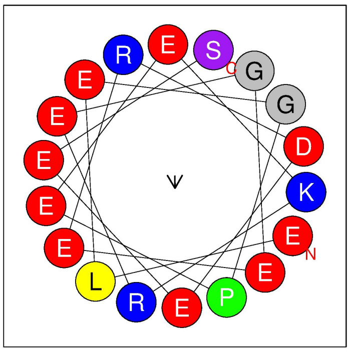

Go to screening | Manual mutation | GA mutation

---

$_{553}$**LEGPEEKREGEERDEESR**$_{570}$

Click to enlarge

**Physico-chemical properties**
**Hydrophobicity <H>**
-0.418
**Hydrophobic moment <μH>**
0.085
**Net charge z**
-5

**Polar residues + GLY**
**Polar residues + GLY (n / %)**
16 / 88.89
**Uncharged residues + GLY**
SER 1, GLY 2
**Charged residues**
LYS 1, ARG 3, GLU 8, ASP 1,
**Hydrophobic face :** none

**Nonpolar residues**
**Nonpolar residues (n / %)**
2 / 11.11
**Aromatic residues**

**Special residues**
CYS 0, PRO 1

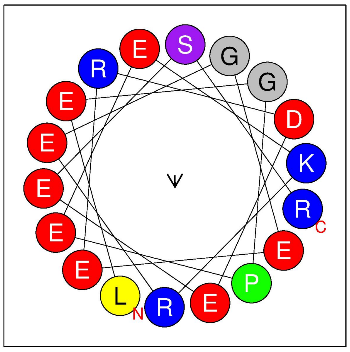

Go to screening | Manual mutation | GA mutation

---

$_{554}$**EGPEEKREGEERDEESRR**$_{571}$

Click to enlarge

**Physico-chemical properties**
**Hydrophobicity <H>**
-0.569
**Hydrophobic moment <μH>**
0.081
**Net charge z**
-4

**Polar residues + GLY**
**Polar residues + GLY (n / %)**
17 / 94.44
**Uncharged residues + GLY**
SER 1, GLY 2
**Charged residues**
LYS 1, ARG 4, GLU 8, ASP 1,
**Hydrophobic face :** none

**Nonpolar residues**
**Nonpolar residues (n / %)**
1 / 5.56
**Aromatic residues**

**Special residues**
CYS 0, PRO 1

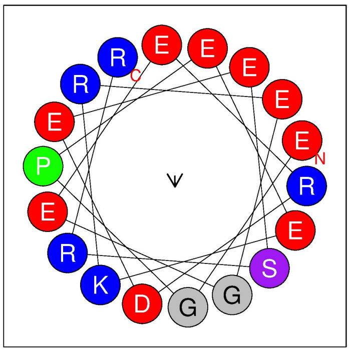

Go to screening | Manual mutation | GA mutation

---

$_{555}$**GPEEKREGEERDEESRRP**$_{572}$

Click to enlarge

**Physico-chemical properties**
**Hydrophobicity <H>**
-0.493

**Polar residues + GLY**
**Polar residues + GLY (n / %)**
16 / 88.89

**Nonpolar residues**
**Nonpolar residues (n / %)**
2 / 11.11

| | | |
|---|---|---|
| **Hydrophobic moment <μH>** | **Uncharged residues + GLY** | **Aromatic residues** |
| 0.095 | SER 1, GLY 2 | |
| **Net charge z** | **Charged residues** | **Special residues** |
| -3 | LYS 1, ARG 4, GLU 7, ASP 1, | CYS 0, PRO 2 |
| | **Hydrophobic face :** none | |
| Go to screening | Manual mutation | GA mutation |

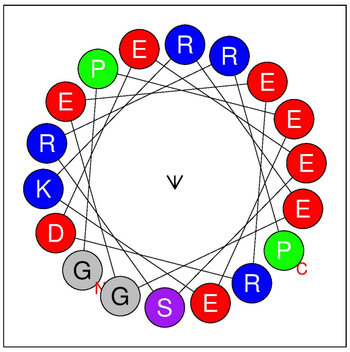


---

$_{556}$**PEEKREGEERDEESRRPC**$_{573}$

Click to enlarge

| | | |
|---|---|---|
| **Physico-chemical properties** | **Polar residues + GLY** | **Nonpolar residues** |
| **Hydrophobicity <H>** | **Polar residues + GLY (n / %)** | **Nonpolar residues (n / %)** |
| -0.408 | 15 / 83.33 | 3 / 16.67 |
| **Hydrophobic moment <μH>** | **Uncharged residues + GLY** | **Aromatic residues** |
| 0.167 | SER 1, GLY 1 | |
| **Net charge z** | **Charged residues** | **Special residues** |
| -3 | LYS 1, ARG 4, GLU 7, ASP 1, | CYS 1, PRO 2 |
| | **Hydrophobic face :** none | |
| Go to screening | Manual mutation | GA mutation |

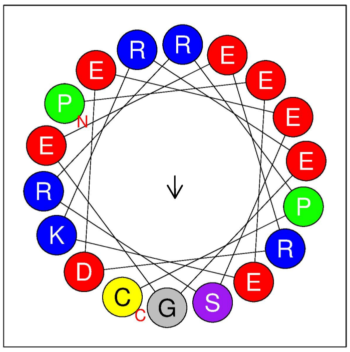


---

$_{557}$**EEKREGEERDEESRRPCA**$_{574}$

Click to enlarge

| | | |
|---|---|---|
| **Physico-chemical properties** | **Polar residues + GLY** | **Nonpolar residues** |
| **Hydrophobicity <H>** | **Polar residues + GLY (n / %)** | **Nonpolar residues (n / %)** |
| -0.431 | 15 / 83.33 | 3 / 16.67 |
| **Hydrophobic moment <μH>** | **Uncharged residues + GLY** | **Aromatic residues** |
| 0.181 | SER 1, GLY 1 | |
| **Net charge z** | **Charged residues** | **Special residues** |
| -3 | LYS 1, ARG 4, GLU 7, ASP 1, | CYS 1, PRO 1 |
| | **Hydrophobic face :** none | |
| Go to screening | Manual mutation | GA mutation |

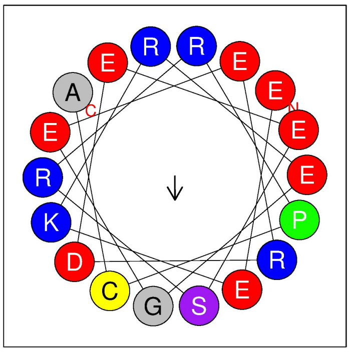


---

$_{558}$**EKREGEERDEESRRPCAM**$_{575}$

Click to enlarge

| | | |
|---|---|---|
| **Physico-chemical properties** | **Polar residues + GLY** | **Nonpolar residues** |
| **Hydrophobicity <H>** | **Polar residues + GLY (n / %)** | **Nonpolar residues (n / %)** |
| -0.327 | 14 / 77.78 | 4 / 22.22 |
| **Hydrophobic moment <μH>** | **Uncharged residues + GLY** | **Aromatic residues** |
| 0.138 | SER 1, GLY 1 | |
| **Net charge z** | **Charged residues** | **Special residues** |
| -2 | LYS 1, ARG 4, GLU 6, ASP 1, | CYS 1, PRO 1 |
| | **Hydrophobic face :** none | |
| Go to screening | Manual mutation | GA mutation |

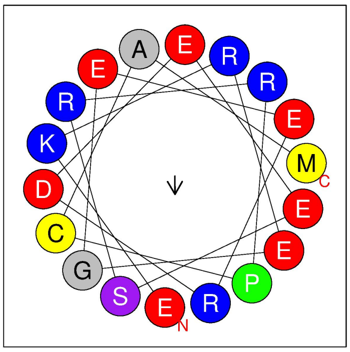


---

$_{559}$**KREGEERDEESRRPCAMV**$_{576}$

Click to enlarge

| | | |
|---|---|---|
| **Physico-chemical properties** | **Polar residues + GLY** | **Nonpolar residues** |
| **Hydrophobicity <H>** | **Polar residues + GLY (n / %)** | **Nonpolar residues (n / %)** |
| -0.223 | 13 / 72.22 | 5 / 27.78 |

| | | |
|---|---|---|
| **Hydrophobic moment <μH>** | **Uncharged residues + GLY** | **Aromatic residues** |
| 0.241 | SER 1, GLY 1 | |
| **Net charge z** | **Charged residues** | **Special residues** |
| -1 | LYS 1, ARG 4, GLU 5, ASP 1, | CYS 1, PRO 1 |
| | **Hydrophobic face :** none | |
| Go to screening | Manual mutation | GA mutation |

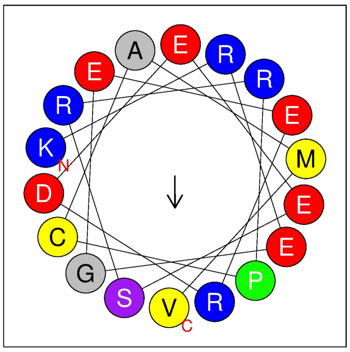

---

$_{560}$**REGEEERDEESRRPCAMVT**$_{577}$

Click to enlarge

| | | |
|---|---|---|
| **Physico-chemical properties** | **Polar residues + GLY** | **Nonpolar residues** |
| **Hydrophobicity <H>** | **Polar residues + GLY (n / %)** | **Nonpolar residues (n / %)** |
| -0.154 | 13 / 72.22 | 5 / 27.78 |
| **Hydrophobic moment <μH>** | **Uncharged residues + GLY** | **Aromatic residues** |
| 0.236 | SER 1, THR 1, GLY 1 | |
| **Net charge z** | **Charged residues** | **Special residues** |
| -2 | ARG 4, GLU 5, ASP 1, | CYS 1, PRO 1 |
| | **Hydrophobic face :** none | |
| Go to screening | Manual mutation | GA mutation |

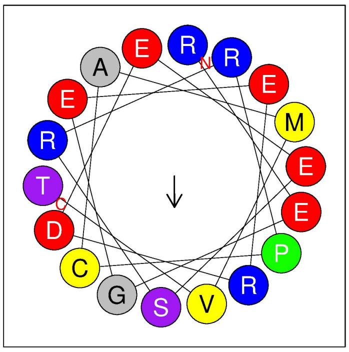

---

$_{561}$**EGEERDEESRRPCAMVTP**$_{578}$

Click to enlarge

| | | |
|---|---|---|
| **Physico-chemical properties** | **Polar residues + GLY** | **Nonpolar residues** |
| **Hydrophobicity <H>** | **Polar residues + GLY (n / %)** | **Nonpolar residues (n / %)** |
| -0.058 | 12 / 66.67 | 6 / 33.33 |
| **Hydrophobic moment <μH>** | **Uncharged residues + GLY** | **Aromatic residues** |
| 0.141 | SER 1, THR 1, GLY 1 | |
| **Net charge z** | **Charged residues** | **Special residues** |
| -3 | ARG 3, GLU 5, ASP 1, | CYS 1, PRO 2 |
| | **Hydrophobic face :** none | |
| Go to screening | Manual mutation | GA mutation |

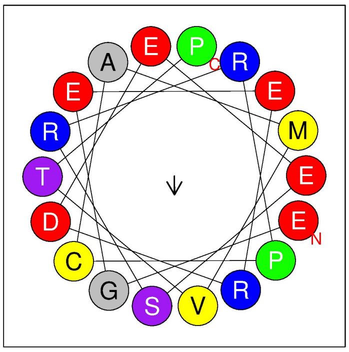

---

$_{562}$**GEERDEESRRPCAMVTPG**$_{579}$

Click to enlarge

| | | |
|---|---|---|
| **Physico-chemical properties** | **Polar residues + GLY** | **Nonpolar residues** |
| **Hydrophobicity <H>** | **Polar residues + GLY (n / %)** | **Nonpolar residues (n / %)** |
| -0.022 | 12 / 66.67 | 6 / 33.33 |
| **Hydrophobic moment <μH>** | **Uncharged residues + GLY** | **Aromatic residues** |
| 0.156 | SER 1, THR 1, GLY 2 | |
| **Net charge z** | **Charged residues** | **Special residues** |
| -2 | ARG 3, GLU 4, ASP 1, | CYS 1, PRO 2 |
| | **Hydrophobic face :** none | |
| Go to screening | Manual mutation | GA mutation |

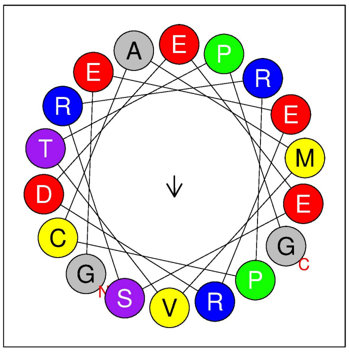

---

$_{563}$**EERDEESRRPCAMVTPGA**$_{580}$

Click to enlarge

| | | |
|---|---|---|
| **Physico-chemical properties** | **Polar residues + GLY** | **Nonpolar residues** |
| **Hydrophobicity <H>** | **Polar residues + GLY (n / %)** | **Nonpolar residues (n / %)** |
| -0.005 | 11 / 61.11 | 7 / 38.89 |

| | | |
|---|---|---|
| **Hydrophobic moment <μH>** | **Uncharged residues + GLY** | **Aromatic residues** |
| 0.170 | SER 1, THR 1, GLY 1 | |
| **Net charge z** | **Charged residues** | **Special residues** |
| -2 | ARG 3, GLU 4, ASP 1, | CYS 1, PRO 2 |
| | **Hydrophobic face :** none | |
| Go to screening | Manual mutation | GA mutation |

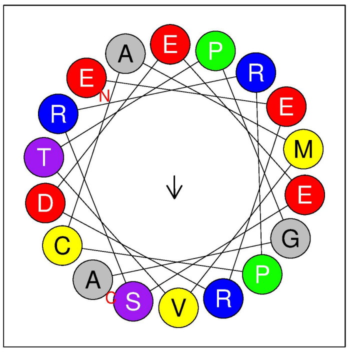


---

$_{564}$**ERDEESRRPCAMVTPGAE**$_{581}$

Click to enlarge

| | | |
|---|---|---|
| **Physico-chemical properties** | **Polar residues + GLY** | **Nonpolar residues** |
| **Hydrophobicity <H>** | **Polar residues + GLY (n / %)** | **Nonpolar residues (n / %)** |
| -0.005 | 11 / 61.11 | 7 / 38.89 |
| **Hydrophobic moment <μH>** | **Uncharged residues + GLY** | **Aromatic residues** |
| 0.170 | SER 1, THR 1, GLY 1 | |
| **Net charge z** | **Charged residues** | **Special residues** |
| -2 | ARG 3, GLU 4, ASP 1, | CYS 1, PRO 2 |
| | **Hydrophobic face :** none | |
| Go to screening | Manual mutation | GA mutation |

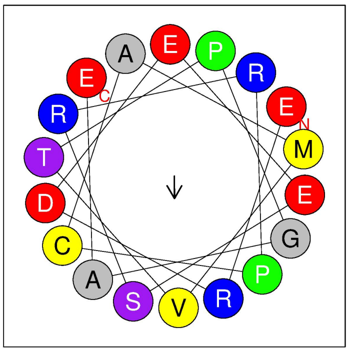


---

$_{565}$**RDEESRRPCAMVTPGAEE**$_{582}$

Click to enlarge

| | | |
|---|---|---|
| **Physico-chemical properties** | **Polar residues + GLY** | **Nonpolar residues** |
| **Hydrophobicity <H>** | **Polar residues + GLY (n / %)** | **Nonpolar residues (n / %)** |
| -0.005 | 11 / 61.11 | 7 / 38.89 |
| **Hydrophobic moment <μH>** | **Uncharged residues + GLY** | **Aromatic residues** |
| 0.170 | SER 1, THR 1, GLY 1 | |
| **Net charge z** | **Charged residues** | **Special residues** |
| -2 | ARG 3, GLU 4, ASP 1, | CYS 1, PRO 2 |
| | **Hydrophobic face :** none | |
| Go to screening | Manual mutation | GA mutation |

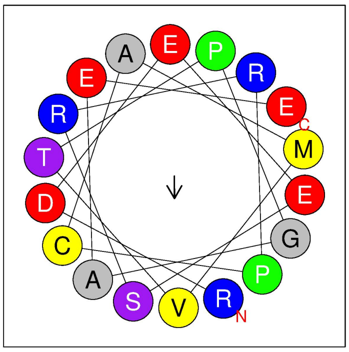


---

$_{566}$**DEESRRPCAMVTPGAEEP**$_{583}$

Click to enlarge

| | | |
|---|---|---|
| **Physico-chemical properties** | **Polar residues + GLY** | **Nonpolar residues** |
| **Hydrophobicity <H>** | **Polar residues + GLY (n / %)** | **Nonpolar residues (n / %)** |
| 0.091 | 10 / 55.56 | 8 / 44.44 |
| **Hydrophobic moment <μH>** | **Uncharged residues + GLY** | **Aromatic residues** |
| 0.261 | SER 1, THR 1, GLY 1 | |
| **Net charge z** | **Charged residues** | **Special residues** |
| -3 | ARG 2, GLU 4, ASP 1, | CYS 1, PRO 3 |
| | **Hydrophobic face :** none | |
| Go to screening | Manual mutation | GA mutation |

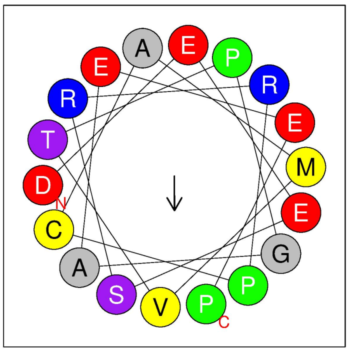


---

$_{567}$**EESRRPCAMVTPGAEEPS**$_{584}$

Click to enlarge

| | | |
|---|---|---|
| **Physico-chemical properties** | **Polar residues + GLY** | **Nonpolar residues** |
| **Hydrophobicity <H>** | **Polar residues + GLY (n / %)** | **Nonpolar residues (n / %)** |
| 0.132 | 10 / 55.56 | 8 / 44.44 |

| | | | |
|---|---|---|---|
| **Hydrophobic moment <μH>** | **Uncharged residues + GLY** | **Aromatic residues** | |
| 0.267 | SER 2, THR 1, GLY 1 | | |
| **Net charge z** | **Charged residues** | **Special residues** | |
| -2 | ARG 2, GLU 4, | CYS 1, PRO 3 | |
| | **Hydrophobic face :** none | | |
| Go to screening | Manual mutation | GA mutation | |

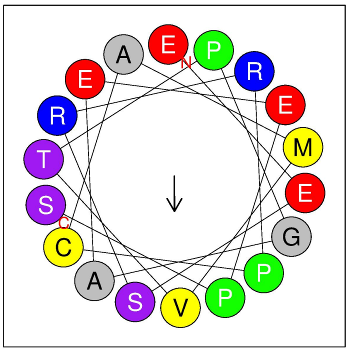


---

$_{568}$**ESRRPCAMVTPGAEEPSI**$_{585}$

Click to enlarge

| | | |
|---|---|---|
| **Physico-chemical properties** | **Polar residues + GLY** | **Nonpolar residues** |
| **Hydrophobicity <H>** | **Polar residues + GLY (n / %)** | **Nonpolar residues (n / %)** |
| 0.267 | 9 / 50.00 | 9 / 50.00 |
| **Hydrophobic moment <μH>** | **Uncharged residues + GLY** | **Aromatic residues** |
| 0.132 | SER 2, THR 1, GLY 1 | |
| **Net charge z** | **Charged residues** | **Special residues** |
| -1 | ARG 2, GLU 3, | CYS 1, PRO 3 |
| | **Hydrophobic face :** none | |
| Go to screening | Manual mutation | GA mutation |

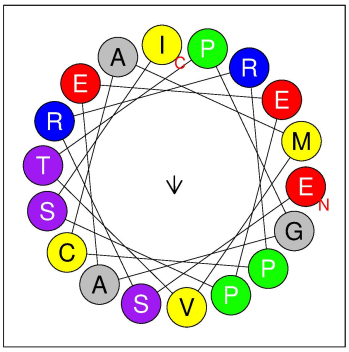


---

$_{569}$**SRRPCAMVTPGAEEPSIP**$_{586}$

Click to enlarge

| | | |
|---|---|---|
| **Physico-chemical properties** | **Polar residues + GLY** | **Nonpolar residues** |
| **Hydrophobicity <H>** | **Polar residues + GLY (n / %)** | **Nonpolar residues (n / %)** |
| 0.343 | 8 / 44.44 | 10 / 55.56 |
| **Hydrophobic moment <μH>** | **Uncharged residues + GLY** | **Aromatic residues** |
| 0.157 | SER 2, THR 1, GLY 1 | |
| **Net charge z** | **Charged residues** | **Special residues** |
| 0 | ARG 2, GLU 2, | CYS 1, PRO 4 |
| | **Hydrophobic face :** M P G P P V | |
| Go to screening | Manual mutation | GA mutation |

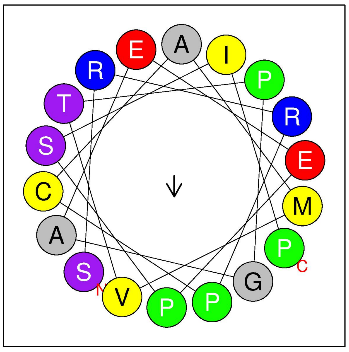


---

$_{570}$**RRPCAMVTPGAEEPSIPE**$_{587}$

Click to enlarge

| | | |
|---|---|---|
| **Physico-chemical properties** | **Polar residues + GLY** | **Nonpolar residues** |
| **Hydrophobicity <H>** | **Polar residues + GLY (n / %)** | **Nonpolar residues (n / %)** |
| 0.309 | 8 / 44.44 | 10 / 55.56 |
| **Hydrophobic moment <μH>** | **Uncharged residues + GLY** | **Aromatic residues** |
| 0.135 | SER 1, THR 1, GLY 1 | |
| **Net charge z** | **Charged residues** | **Special residues** |
| -1 | ARG 2, GLU 3, | CYS 1, PRO 4 |
| | **Hydrophobic face :** M P G P P V | |
| Go to screening | Manual mutation | GA mutation |

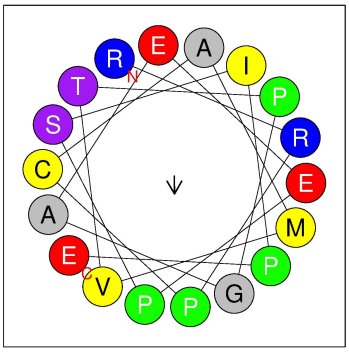


---

$_{571}$**RPCAMVTPGAEEPSIPEP**$_{588}$

Click to enlarge

| | | |
|---|---|---|
| **Physico-chemical properties** | **Polar residues + GLY** | **Nonpolar residues** |
| **Hydrophobicity <H>** | **Polar residues + GLY (n / %)** | **Nonpolar residues (n / %)** |
| 0.406 | 7 / 38.89 | 11 / 61.11 |

| | | | |
|---|---|---|---|
| **Hydrophobic moment <μH>** | **Uncharged residues + GLY** | **Aromatic residues** | 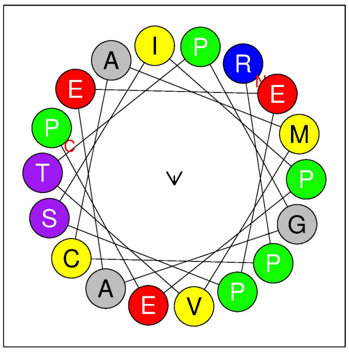 |
| 0.066 | SER 1, THR 1, GLY 1 | | |
| **Net charge z** | **Charged residues** | **Special residues** | |
| -2 | ARG 1, GLU 3, | CYS 1, PRO 5 | |
| | **Hydrophobic face :** M P G P P V | | |
| [Go to screening]() | [Manual mutation]() | [GA mutation]() | |

---

$_{572}$**PCAMVTPGAEEPSIPEPP**$_{589}$

Click to enlarge

| | | | |
|---|---|---|---|
| **Physico-chemical properties** | **Polar residues + GLY** | **Nonpolar residues** | 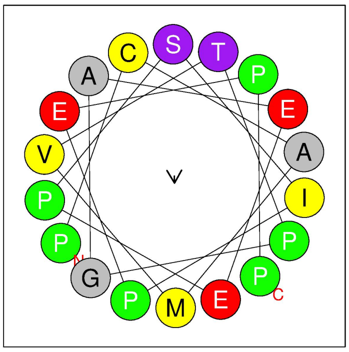 |
| **Hydrophobicity <H>** | **Polar residues + GLY (n / %)** | **Nonpolar residues (n / %)** | |
| 0.502 | 6 / 33.33 | 12 / 66.67 | |
| **Hydrophobic moment <μH>** | **Uncharged residues + GLY** | **Aromatic residues** | |
| 0.053 | SER 1, THR 1, GLY 1 | | |
| **Net charge z** | **Charged residues** | **Special residues** | |
| -3 | GLU 3, | CYS 1, PRO 6 | |
| | **Hydrophobic face :** M P G P P V | | |
| [Go to screening]() | [Manual mutation]() | [GA mutation]() | |

---

$_{573}$**CAMVTPGAEEPSIPEPPK**$_{590}$

Click to enlarge

| | | | |
|---|---|---|---|
| **Physico-chemical properties** | **Polar residues + GLY** | **Nonpolar residues** | 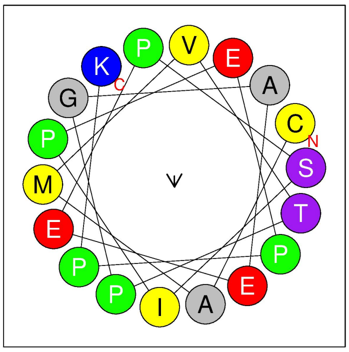 |
| **Hydrophobicity <H>** | **Polar residues + GLY (n / %)** | **Nonpolar residues (n / %)** | |
| 0.407 | 7 / 38.89 | 11 / 61.11 | |
| **Hydrophobic moment <μH>** | **Uncharged residues + GLY** | **Aromatic residues** | |
| 0.082 | SER 1, THR 1, GLY 1 | | |
| **Net charge z** | **Charged residues** | **Special residues** | |
| -2 | LYS 1, GLU 3, | CYS 1, PRO 5 | |
| | **Hydrophobic face :** none | | |
| [Go to screening]() | [Manual mutation]() | [GA mutation]() | |

---

$_{574}$**AMVTPGAEEPSIPEPPKP**$_{591}$

Click to enlarge

| | | | |
|---|---|---|---|
| **Physico-chemical properties** | **Polar residues + GLY** | **Nonpolar residues** | 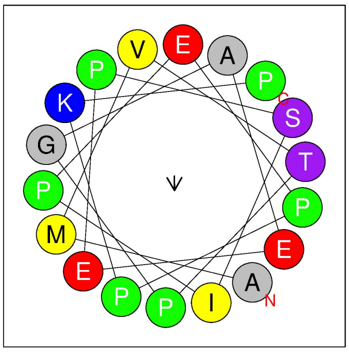 |
| **Hydrophobicity <H>** | **Polar residues + GLY (n / %)** | **Nonpolar residues (n / %)** | |
| 0.361 | 7 / 38.89 | 11 / 61.11 | |
| **Hydrophobic moment <μH>** | **Uncharged residues + GLY** | **Aromatic residues** | |
| 0.109 | SER 1, THR 1, GLY 1 | | |
| **Net charge z** | **Charged residues** | **Special residues** | |
| -2 | LYS 1, GLU 3, | CYS 0, PRO 6 | |
| | **Hydrophobic face :** none | | |
| [Go to screening]() | [Manual mutation]() | [GA mutation]() | |

---

$_{575}$**MVTPGAEEPSIPEPPKPA**$_{592}$

Click to enlarge

| | | |
|---|---|---|
| **Physico-chemical properties** | **Polar residues + GLY** | **Nonpolar residues** |
| **Hydrophobicity <H>** | **Polar residues + GLY (n / %)** | **Nonpolar residues (n / %)** |
| 0.361 | 7 / 38.89 | 11 / 61.11 |

**Hydrophobic moment <μH>**
0.109
**Net charge z**
-2

**Uncharged residues + GLY**
SER 1, THR 1, GLY 1
**Charged residues**
LYS 1, GLU 3,
**Hydrophobic face :** none

**Aromatic residues**

**Special residues**
CYS 0, PRO 6

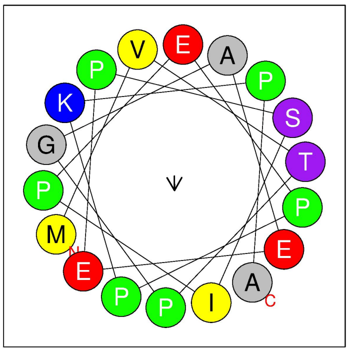


Go to screening    Manual mutation    GA mutation

---

$_{576}$**VTPGAEEPSIPEPPKPAA**$_{593}$

Click to enlarge

**Physico-chemical properties**
**Hydrophobicity <H>**
0.310
**Hydrophobic moment <μH>**
0.097
**Net charge z**
-2

**Polar residues + GLY**
**Polar residues + GLY (n / %)**
7 / 38.89
**Uncharged residues + GLY**
SER 1, THR 1, GLY 1
**Charged residues**
LYS 1, GLU 3,
**Hydrophobic face :** none

**Nonpolar residues**
**Nonpolar residues (n / %)**
11 / 61.11
**Aromatic residues**

**Special residues**
CYS 0, PRO 6

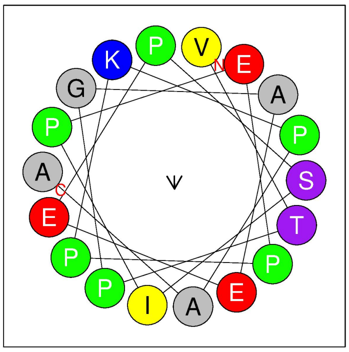


Go to screening    Manual mutation    GA mutation

---

$_{577}$**TPGAEEPSIPEPPKPAAD**$_{594}$

Click to enlarge

**Physico-chemical properties**
**Hydrophobicity <H>**
0.199
**Hydrophobic moment <μH>**
0.207
**Net charge z**
-3

**Polar residues + GLY**
**Polar residues + GLY (n / %)**
8 / 44.44
**Uncharged residues + GLY**
SER 1, THR 1, GLY 1
**Charged residues**
LYS 1, GLU 3, ASP 1,
**Hydrophobic face :** none

**Nonpolar residues**
**Nonpolar residues (n / %)**
10 / 55.56
**Aromatic residues**

**Special residues**
CYS 0, PRO 6

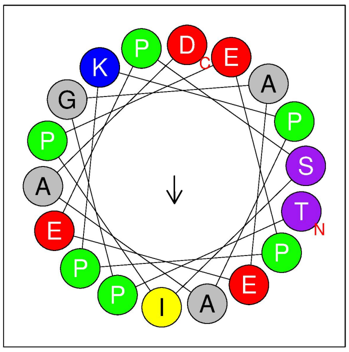


Go to screening    Manual mutation    GA mutation

---

$_{578}$**PGAEEPSIPEPPKPAADQ**$_{595}$

Click to enlarge

**Physico-chemical properties**
**Hydrophobicity <H>**
0.173
**Hydrophobic moment <μH>**
0.201
**Net charge z**
-3

**Polar residues + GLY**
**Polar residues + GLY (n / %)**
8 / 44.44
**Uncharged residues + GLY**
GLN 1, SER 1, GLY 1
**Charged residues**
LYS 1, GLU 3, ASP 1,
**Hydrophobic face :** none

**Nonpolar residues**
**Nonpolar residues (n / %)**
10 / 55.56
**Aromatic residues**

**Special residues**
CYS 0, PRO 6

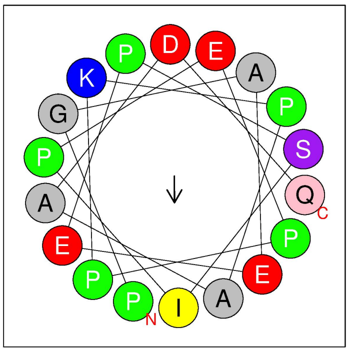


Go to screening    Manual mutation    GA mutation

---

$_{579}$**GAEEPSIPEPPKPAADQD**$_{596}$

Click to enlarge

**Physico-chemical properties**
**Hydrophobicity <H>**
0.090

**Polar residues + GLY**
**Polar residues + GLY (n / %)**
9 / 50.00

**Nonpolar residues**
**Nonpolar residues (n / %)**
9 / 50.00

| | | |
|---|---|---|
| **Hydrophobic moment <μH>** | **Uncharged residues + GLY** | **Aromatic residues** |
| 0.125 | GLN 1, SER 1, GLY 1 | |
| **Net charge z** | **Charged residues** | **Special residues** |
| -4 | LYS 1, GLU 3, ASP 2, | CYS 0, PRO 5 |
| | **Hydrophobic face :** none | |
| Go to screening | Manual mutation | GA mutation |

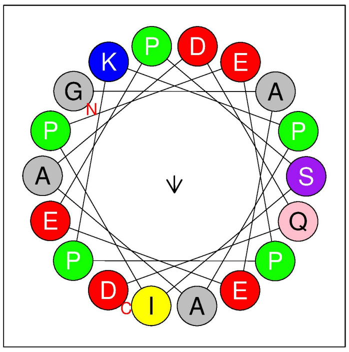


---

**$_{580}$AEEPSIPEPPKPAADQDG$_{597}$**

Click to enlarge

| | | |
|---|---|---|
| **Physico-chemical properties** | **Polar residues + GLY** | **Nonpolar residues** |
| **Hydrophobicity <H>** | **Polar residues + GLY (n / %)** | **Nonpolar residues (n / %)** |
| 0.090 | 9 / 50.00 | 9 / 50.00 |
| **Hydrophobic moment <μH>** | **Uncharged residues + GLY** | **Aromatic residues** |
| 0.125 | GLN 1, SER 1, GLY 1 | |
| **Net charge z** | **Charged residues** | **Special residues** |
| -4 | LYS 1, GLU 3, ASP 2, | CYS 0, PRO 5 |
| | **Hydrophobic face :** none | |
| Go to screening | Manual mutation | GA mutation |

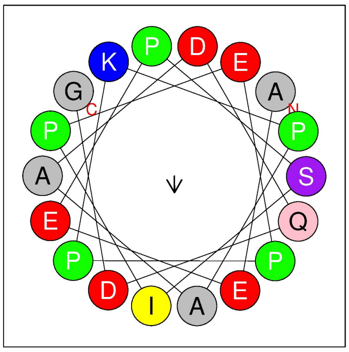


---

**$_{581}$EEPSIPEPPKPAADQDGA$_{598}$**

Click to enlarge

| | | |
|---|---|---|
| **Physico-chemical properties** | **Polar residues + GLY** | **Nonpolar residues** |
| **Hydrophobicity <H>** | **Polar residues + GLY (n / %)** | **Nonpolar residues (n / %)** |
| 0.090 | 9 / 50.00 | 9 / 50.00 |
| **Hydrophobic moment <μH>** | **Uncharged residues + GLY** | **Aromatic residues** |
| 0.125 | GLN 1, SER 1, GLY 1 | |
| **Net charge z** | **Charged residues** | **Special residues** |
| -4 | LYS 1, GLU 3, ASP 2, | CYS 0, PRO 5 |
| | **Hydrophobic face :** none | |
| Go to screening | Manual mutation | GA mutation |

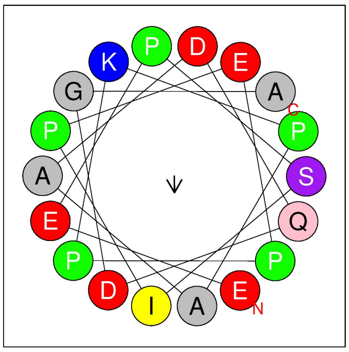


---

**$_{582}$EPSIPEPPKPAADQDGAE$_{599}$**

Click to enlarge

| | | |
|---|---|---|
| **Physico-chemical properties** | **Polar residues + GLY** | **Nonpolar residues** |
| **Hydrophobicity <H>** | **Polar residues + GLY (n / %)** | **Nonpolar residues (n / %)** |
| 0.090 | 9 / 50.00 | 9 / 50.00 |
| **Hydrophobic moment <μH>** | **Uncharged residues + GLY** | **Aromatic residues** |
| 0.125 | GLN 1, SER 1, GLY 1 | |
| **Net charge z** | **Charged residues** | **Special residues** |
| -4 | LYS 1, GLU 3, ASP 2, | CYS 0, PRO 5 |
| | **Hydrophobic face :** none | |
| Go to screening | Manual mutation | GA mutation |

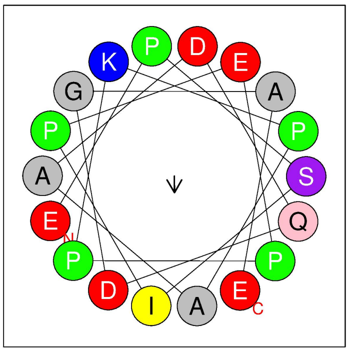


---

**$_{583}$PSIPEPPKPAADQDGAEV$_{600}$**

Click to enlarge

| | | |
|---|---|---|
| **Physico-chemical properties** | **Polar residues + GLY** | **Nonpolar residues** |
| **Hydrophobicity <H>** | **Polar residues + GLY (n / %)** | **Nonpolar residues (n / %)** |
| 0.193 | 8 / 44.44 | 10 / 55.56 |

**Hydrophobic moment <μH>**
0.188
**Net charge z**
-3

**Uncharged residues + GLY**
GLN 1, SER 1, GLY 1
**Charged residues**
LYS 1, GLU 2, ASP 2,
**Hydrophobic face :** none

**Aromatic residues**

**Special residues**
CYS 0, PRO 5

Go to screening | Manual mutation | GA mutation

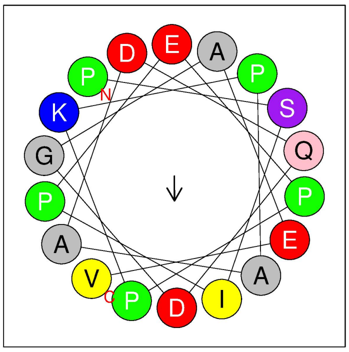


---

**${}_{584}$SIPEPPKPAADQDGAEVL${}_{601}$**

Click to enlarge

**Physico-chemical properties**
**Hydrophobicity <H>**
0.248
**Hydrophobic moment <μH>**
0.151
**Net charge z**
-3

**Polar residues + GLY**
**Polar residues + GLY (n / %)**
8 / 44.44
**Uncharged residues + GLY**
GLN 1, SER 1, GLY 1
**Charged residues**
LYS 1, GLU 2, ASP 2,
**Hydrophobic face :** none

**Nonpolar residues**
**Nonpolar residues (n / %)**
10 / 55.56
**Aromatic residues**

**Special residues**
CYS 0, PRO 4

Go to screening | Manual mutation | GA mutation

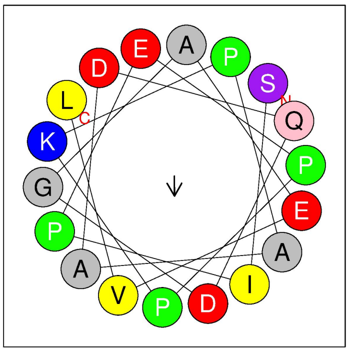


---

**${}_{585}$IPEPPKPAADQDGAEVLG${}_{602}$**

Click to enlarge

**Physico-chemical properties**
**Hydrophobicity <H>**
0.250
**Hydrophobic moment <μH>**
0.149
**Net charge z**
-3

**Polar residues + GLY**
**Polar residues + GLY (n / %)**
8 / 44.44
**Uncharged residues + GLY**
GLN 1, GLY 2
**Charged residues**
LYS 1, GLU 2, ASP 2,
**Hydrophobic face :** none

**Nonpolar residues**
**Nonpolar residues (n / %)**
10 / 55.56
**Aromatic residues**

**Special residues**
CYS 0, PRO 4

Go to screening | Manual mutation | GA mutation

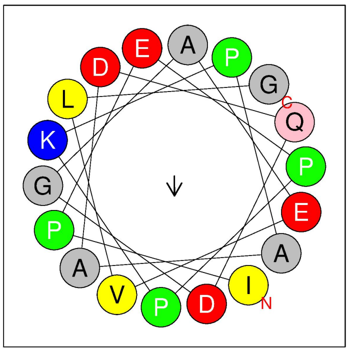


---

**${}_{586}$PEPPKPAADQDGAEVLGT${}_{603}$**

Click to enlarge

**Physico-chemical properties**
**Hydrophobicity <H>**
0.164
**Hydrophobic moment <μH>**
0.092
**Net charge z**
-3

**Polar residues + GLY**
**Polar residues + GLY (n / %)**
9 / 50.00
**Uncharged residues + GLY**
GLN 1, THR 1, GLY 2
**Charged residues**
LYS 1, GLU 2, ASP 2,
**Hydrophobic face :** none

**Nonpolar residues**
**Nonpolar residues (n / %)**
9 / 50.00
**Aromatic residues**

**Special residues**
CYS 0, PRO 4

Go to screening | Manual mutation | GA mutation

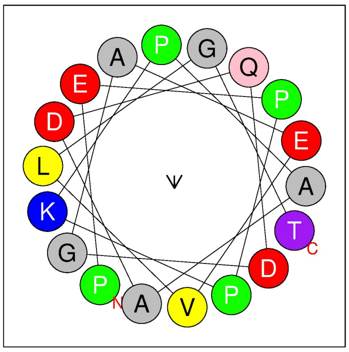


---

**${}_{587}$EPPKPAADQDGAEVLGTR${}_{604}$**

Click to enlarge

**Physico-chemical properties**
**Hydrophobicity <H>**
0.068

**Polar residues + GLY**
**Polar residues + GLY (n / %)**
10 / 55.56

**Nonpolar residues**
**Nonpolar residues (n / %)**
8 / 44.44

| | | |
|---|---|---|
| **Hydrophobic moment <μH>** | **Uncharged residues + GLY** | **Aromatic residues** |
| 0.056 | GLN 1, THR 1, GLY 2 | |
| **Net charge z** | **Charged residues** | **Special residues** |
| -2 | LYS 1, ARG 1, GLU 2, ASP 2, | CYS 0, PRO 3 |
| | **Hydrophobic face :** none | |
| Go to screening | Manual mutation | GA mutation |

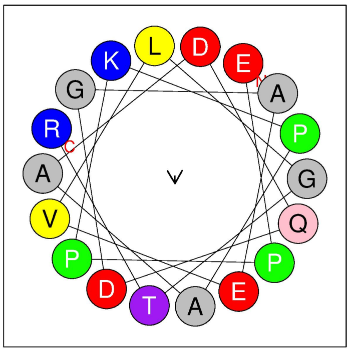

---

$_{588}$**PPKPAADQDGAEVLGTRS**$_{605}$

Click to enlarge

| | | |
|---|---|---|
| **Physico-chemical properties** | **Polar residues + GLY** | **Nonpolar residues** |
| **Hydrophobicity <H>** | **Polar residues + GLY (n / %)** | **Nonpolar residues (n / %)** |
| 0.102 | 10 / 55.56 | 8 / 44.44 |
| **Hydrophobic moment <μH>** | **Uncharged residues + GLY** | **Aromatic residues** |
| 0.032 | GLN 1, SER 1, THR 1, GLY 2 | |
| **Net charge z** | **Charged residues** | **Special residues** |
| -1 | LYS 1, ARG 1, GLU 1, ASP 2, | CYS 0, PRO 3 |
| | **Hydrophobic face :** none | |
| Go to screening | Manual mutation | GA mutation |

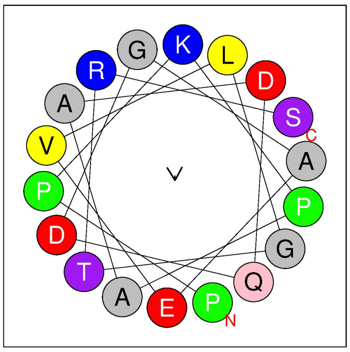

---

$_{589}$**PKPAADQDGAEVLGTRSR**$_{606}$

Click to enlarge

| | | |
|---|---|---|
| **Physico-chemical properties** | **Polar residues + GLY** | **Nonpolar residues** |
| **Hydrophobicity <H>** | **Polar residues + GLY (n / %)** | **Nonpolar residues (n / %)** |
| 0.006 | 11 / 61.11 | 7 / 38.89 |
| **Hydrophobic moment <μH>** | **Uncharged residues + GLY** | **Aromatic residues** |
| 0.066 | GLN 1, SER 1, THR 1, GLY 2 | |
| **Net charge z** | **Charged residues** | **Special residues** |
| 0 | LYS 1, ARG 2, GLU 1, ASP 2, | CYS 0, PRO 2 |
| | **Hydrophobic face :** none | |
| Go to screening | Manual mutation | GA mutation |

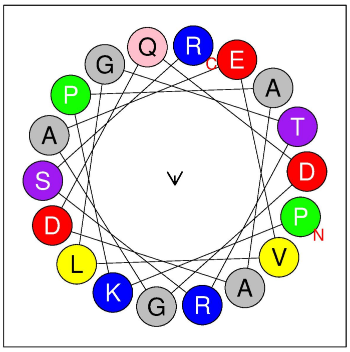

---

$_{590}$**KPAADQDGAEVLGTRSRS**$_{607}$

Click to enlarge

| | | |
|---|---|---|
| **Physico-chemical properties** | **Polar residues + GLY** | **Nonpolar residues** |
| **Hydrophobicity <H>** | **Polar residues + GLY (n / %)** | **Nonpolar residues (n / %)** |
| -0.037 | 12 / 66.67 | 6 / 33.33 |
| **Hydrophobic moment <μH>** | **Uncharged residues + GLY** | **Aromatic residues** |
| 0.066 | GLN 1, SER 2, THR 1, GLY 2 | |
| **Net charge z** | **Charged residues** | **Special residues** |
| 0 | LYS 1, ARG 2, GLU 1, ASP 2, | CYS 0, PRO 1 |
| | **Hydrophobic face :** none | |
| Go to screening | Manual mutation | GA mutation |

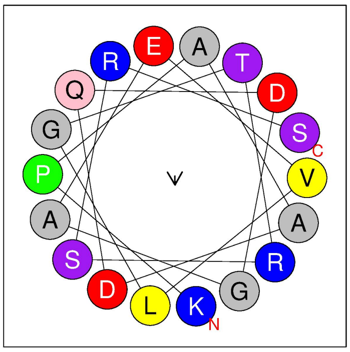

---

$_{591}$**PAADQDGAEVLGTRSRSL**$_{608}$

Click to enlarge

| | | |
|---|---|---|
| **Physico-chemical properties** | **Polar residues + GLY** | **Nonpolar residues** |
| **Hydrophobicity <H>** | **Polar residues + GLY (n / %)** | **Nonpolar residues (n / %)** |
| 0.113 | 11 / 61.11 | 7 / 38.89 |

**Hydrophobic moment <μH>**
0.215
**Net charge z**
-1

**Uncharged residues + GLY**
GLN 1, SER 2, THR 1, GLY 2
**Charged residues**
ARG 2, GLU 1, ASP 2,
**Hydrophobic face :** none

**Aromatic residues**

**Special residues**
CYS 0, PRO 1

Go to screening | Manual mutation | GA mutation

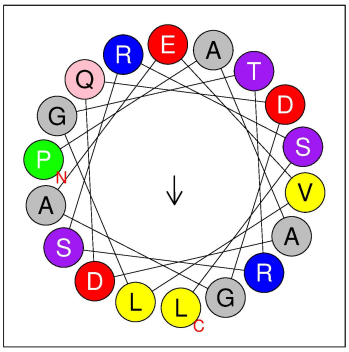


---

## $_{592}$AADQDGAEVLGTRSRSLP$_{609}$

Click to enlarge

**Physico-chemical properties**
**Hydrophobicity <H>**
0.113
**Hydrophobic moment <μH>**
0.215
**Net charge z**
-1

**Polar residues + GLY**
**Polar residues + GLY (n / %)**
11 / 61.11
**Uncharged residues + GLY**
GLN 1, SER 2, THR 1, GLY 2
**Charged residues**
ARG 2, GLU 1, ASP 2,
**Hydrophobic face :** none

**Nonpolar residues**
**Nonpolar residues (n / %)**
7 / 38.89
**Aromatic residues**

**Special residues**
CYS 0, PRO 1

Go to screening | Manual mutation | GA mutation

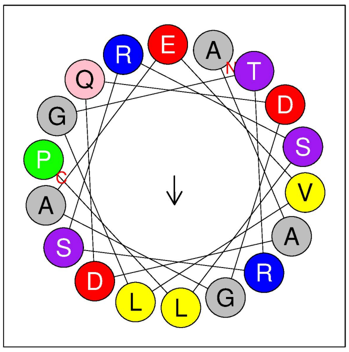


---

## $_{593}$ADQDGAEVLGTRSRSLPE$_{610}$

Click to enlarge

**Physico-chemical properties**
**Hydrophobicity <H>**
0.060
**Hydrophobic moment <μH>**
0.266
**Net charge z**
-2

**Polar residues + GLY**
**Polar residues + GLY (n / %)**
12 / 66.67
**Uncharged residues + GLY**
GLN 1, SER 2, THR 1, GLY 2
**Charged residues**
ARG 2, GLU 2, ASP 2,
**Hydrophobic face :** none

**Nonpolar residues**
**Nonpolar residues (n / %)**
6 / 33.33
**Aromatic residues**

**Special residues**
CYS 0, PRO 1

Go to screening | Manual mutation | GA mutation

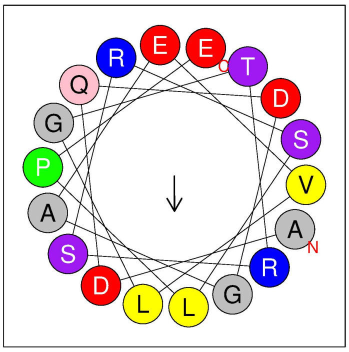


---

## $_{594}$DQDGAEVLGTRSRSLPEK$_{611}$

Click to enlarge

**Physico-chemical properties**
**Hydrophobicity <H>**
-0.012
**Hydrophobic moment <μH>**
0.246
**Net charge z**
-1

**Polar residues + GLY**
**Polar residues + GLY (n / %)**
13 / 72.22
**Uncharged residues + GLY**
GLN 1, SER 2, THR 1, GLY 2
**Charged residues**
LYS 1, ARG 2, GLU 2, ASP 2,
**Hydrophobic face :** none

**Nonpolar residues**
**Nonpolar residues (n / %)**
5 / 27.78
**Aromatic residues**

**Special residues**
CYS 0, PRO 1

Go to screening | Manual mutation | GA mutation

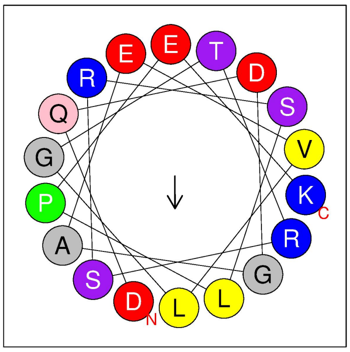


---

## $_{595}$QDGAEVLGTRSRSLPEKG$_{612}$

Click to enlarge

**Physico-chemical properties**
**Hydrophobicity <H>**
0.031

**Polar residues + GLY**
**Polar residues + GLY (n / %)**
13 / 72.22

**Nonpolar residues**
**Nonpolar residues (n / %)**
5 / 27.78

| | | | |
|---|---|---|---|
| **Hydrophobic moment <μH>** | **Uncharged residues + GLY** | **Aromatic residues** | 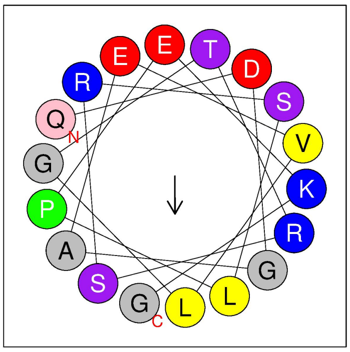 |
| 0.287 | GLN 1, SER 2, THR 1, GLY 3 | | |
| **Net charge z** | **Charged residues** | **Special residues** | |
| 0 | LYS 1, ARG 2, GLU 2, ASP 1, | CYS 0, PRO 1 | |
| | **Hydrophobic face :** none | | |
| Go to screening | Manual mutation | GA mutation | |

---

$_{596}$**DGAEVLGTRSRSLPEKGP**$_{613}$

Click to enlarge

| | | | |
|---|---|---|---|
| **Physico-chemical properties** | **Polar residues + GLY** | **Nonpolar residues** | 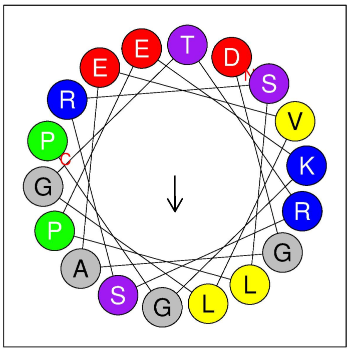 |
| **Hydrophobicity <H>** | **Polar residues + GLY (n / %)** | **Nonpolar residues (n / %)** | |
| 0.083 | 12 / 66.67 | 6 / 33.33 | |
| **Hydrophobic moment <μH>** | **Uncharged residues + GLY** | **Aromatic residues** | |
| 0.269 | SER 2, THR 1, GLY 3 | | |
| **Net charge z** | **Charged residues** | **Special residues** | |
| 0 | LYS 1, ARG 2, GLU 2, ASP 1, | CYS 0, PRO 2 | |
| | **Hydrophobic face :** none | | |
| Go to screening | Manual mutation | GA mutation | |

---

$_{597}$**GAEVLGTRSRSLPEKGPP**$_{614}$

Click to enlarge

| | | | |
|---|---|---|---|
| **Physico-chemical properties** | **Polar residues + GLY** | **Nonpolar residues** | 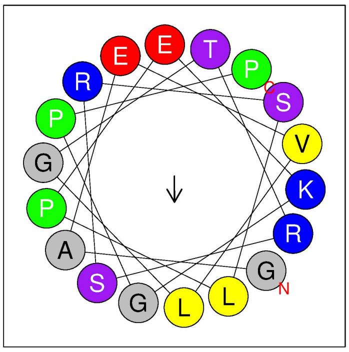 |
| **Hydrophobicity <H>** | **Polar residues + GLY (n / %)** | **Nonpolar residues (n / %)** | |
| 0.166 | 11 / 61.11 | 7 / 38.89 | |
| **Hydrophobic moment <μH>** | **Uncharged residues + GLY** | **Aromatic residues** | |
| 0.197 | SER 2, THR 1, GLY 3 | | |
| **Net charge z** | **Charged residues** | **Special residues** | |
| 1 | LYS 1, ARG 2, GLU 2, | CYS 0, PRO 3 | |
| | **Hydrophobic face :** none | | |
| Go to screening | Manual mutation | GA mutation | |

---

$_{598}$**AEVLGTRSRSLPEKGPPK**$_{615}$

Click to enlarge

| | | | |
|---|---|---|---|
| **Physico-chemical properties** | **Polar residues + GLY** | **Nonpolar residues** | 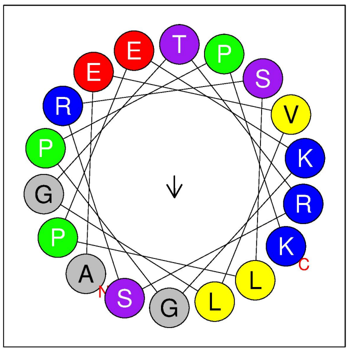 |
| **Hydrophobicity <H>** | **Polar residues + GLY (n / %)** | **Nonpolar residues (n / %)** | |
| 0.111 | 11 / 61.11 | 7 / 38.89 | |
| **Hydrophobic moment <μH>** | **Uncharged residues + GLY** | **Aromatic residues** | |
| 0.162 | SER 2, THR 1, GLY 2 | | |
| **Net charge z** | **Charged residues** | **Special residues** | |
| 2 | LYS 2, ARG 2, GLU 2, | CYS 0, PRO 3 | |
| | **Hydrophobic face :** none | | |
| Go to screening | Manual mutation | GA mutation | |

---

$_{599}$**EVLGTRSRSLPEKGPPKA**$_{616}$

Click to enlarge

| | | |
|---|---|---|
| **Physico-chemical properties** | **Polar residues + GLY** | **Nonpolar residues** |
| **Hydrophobicity <H>** | **Polar residues + GLY (n / %)** | **Nonpolar residues (n / %)** |
| 0.111 | 11 / 61.11 | 7 / 38.89 |

**Hydrophobic moment <μH>**
0.162
**Net charge z**
2

**Uncharged residues + GLY**
SER 2, THR 1, GLY 2
**Charged residues**
LYS 2, ARG 2, GLU 2,
**Hydrophobic face :** none

**Aromatic residues**

**Special residues**
CYS 0, PRO 3

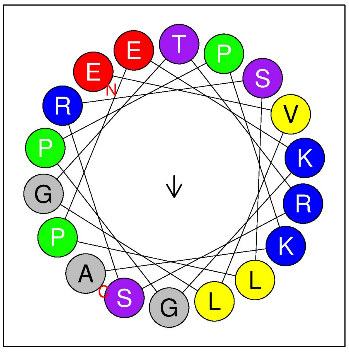

Go to screening | Manual mutation | GA mutation

---

$_{600}$**VLGTRSRSLPEKGPPKAL**$_{617}$

Click to enlarge

**Physico-chemical properties**
**Hydrophobicity <H>**
0.241
**Hydrophobic moment <μH>**
0.099
**Net charge z**
3

**Polar residues + GLY**
**Polar residues + GLY (n / %)**
10 / 55.56
**Uncharged residues + GLY**
SER 2, THR 1, GLY 2
**Charged residues**
LYS 2, ARG 2, GLU 1,
**Hydrophobic face :** none

**Nonpolar residues**
**Nonpolar residues (n / %)**
8 / 44.44
**Aromatic residues**

**Special residues**
CYS 0, PRO 3

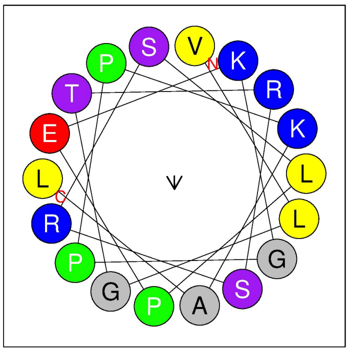

Go to screening | Manual mutation | GA mutation

---

$_{601}$**LGTRSRSLPEKGPPKALA**$_{618}$

Click to enlarge

**Physico-chemical properties**
**Hydrophobicity <H>**
0.190
**Hydrophobic moment <μH>**
0.150
**Net charge z**
3

**Polar residues + GLY**
**Polar residues + GLY (n / %)**
10 / 55.56
**Uncharged residues + GLY**
SER 2, THR 1, GLY 2
**Charged residues**
LYS 2, ARG 2, GLU 1,
**Hydrophobic face :** none

**Nonpolar residues**
**Nonpolar residues (n / %)**
8 / 44.44
**Aromatic residues**

**Special residues**
CYS 0, PRO 3

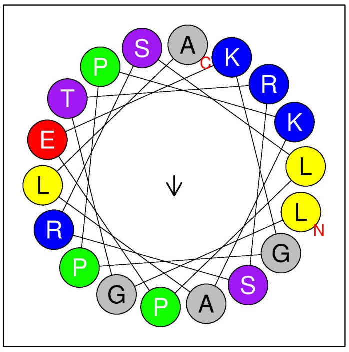

Go to screening | Manual mutation | GA mutation

---

$_{602}$**GTRSRSLPEKGPPKALAY**$_{619}$

Click to enlarge

**Physico-chemical properties**
**Hydrophobicity <H>**
0.149
**Hydrophobic moment <μH>**
0.144
**Net charge z**
3

**Polar residues + GLY**
**Polar residues + GLY (n / %)**
10 / 55.56
**Uncharged residues + GLY**
SER 2, THR 1, GLY 2
**Charged residues**
LYS 2, ARG 2, GLU 1,
**Hydrophobic face :** none

**Nonpolar residues**
**Nonpolar residues (n / %)**
8 / 44.44
**Aromatic residues**
TYR 1,
**Special residues**
CYS 0, PRO 3

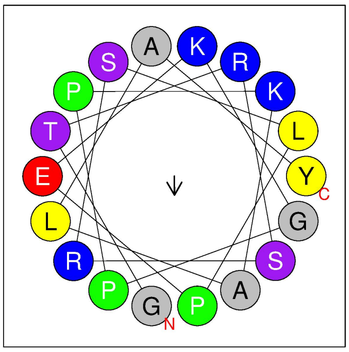

Go to screening | Manual mutation | GA mutation

---

$_{603}$**TRSRSLPEKGPPKALAYK**$_{620}$

Click to enlarge

**Physico-chemical properties**
**Hydrophobicity <H>**
0.094

**Polar residues + GLY**
**Polar residues + GLY (n / %)**
10 / 55.56

**Nonpolar residues**
**Nonpolar residues (n / %)**
8 / 44.44

**Hydrophobic moment <μH>** 0.090
**Net charge z** 4

**Uncharged residues + GLY** SER 2, THR 1, GLY 1
**Charged residues** LYS 3, ARG 2, GLU 1,
**Hydrophobic face :** none

**Aromatic residues** TYR 1,
**Special residues** CYS 0, PRO 3

Go to screening | Manual mutation | GA mutation

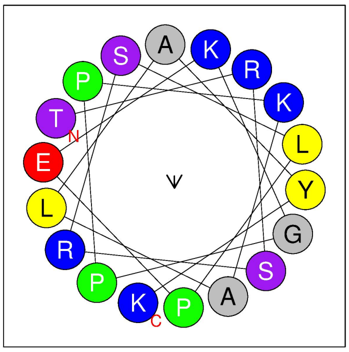


---

## $_{604}$RSRSLPEKGPPKALAYKT$_{621}$

Click to enlarge

**Physico-chemical properties**
**Hydrophobicity <H>** 0.094
**Hydrophobic moment <μH>** 0.090
**Net charge z** 4

**Polar residues + GLY**
**Polar residues + GLY (n / %)** 10 / 55.56
**Uncharged residues + GLY** SER 2, THR 1, GLY 1
**Charged residues** LYS 3, ARG 2, GLU 1,
**Hydrophobic face :** none

**Nonpolar residues**
**Nonpolar residues (n / %)** 8 / 44.44
**Aromatic residues** TYR 1,
**Special residues** CYS 0, PRO 3

Go to screening | Manual mutation | GA mutation

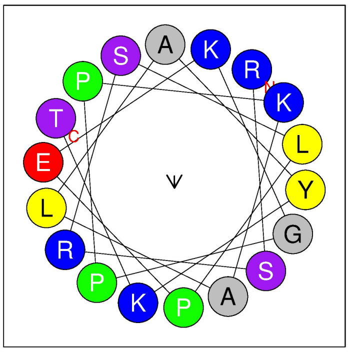


---

## $_{605}$SRSLPEKGPPKALAYKTV$_{622}$

Click to enlarge

**Physico-chemical properties**
**Hydrophobicity <H>** 0.218
**Hydrophobic moment <μH>** 0.074
**Net charge z** 3

**Polar residues + GLY**
**Polar residues + GLY (n / %)** 9 / 50.00
**Uncharged residues + GLY** SER 2, THR 1, GLY 1
**Charged residues** LYS 3, ARG 1, GLU 1,
**Hydrophobic face :** none

**Nonpolar residues**
**Nonpolar residues (n / %)** 9 / 50.00
**Aromatic residues** TYR 1,
**Special residues** CYS 0, PRO 3

Go to screening | Manual mutation | GA mutation

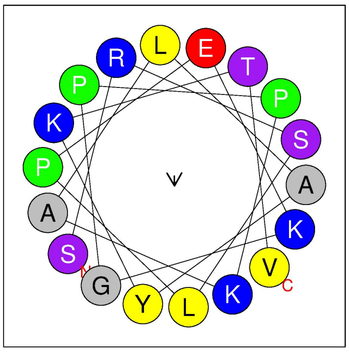


---

## $_{606}$RSLPEKGPPKALAYKTVE$_{623}$

Click to enlarge

**Physico-chemical properties**
**Hydrophobicity <H>** 0.184
**Hydrophobic moment <μH>** 0.060
**Net charge z** 2

**Polar residues + GLY**
**Polar residues + GLY (n / %)** 9 / 50.00
**Uncharged residues + GLY** SER 1, THR 1, GLY 1
**Charged residues** LYS 3, ARG 1, GLU 2,
**Hydrophobic face :** none

**Nonpolar residues**
**Nonpolar residues (n / %)** 9 / 50.00
**Aromatic residues** TYR 1,
**Special residues** CYS 0, PRO 3

Go to screening | Manual mutation | GA mutation

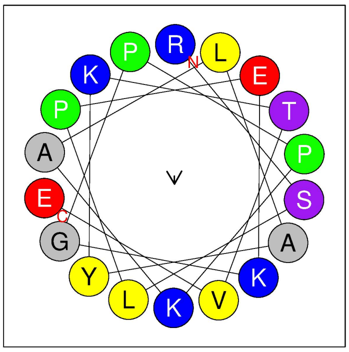


---

## $_{607}$SLPEKGPPKALAYKTVEV$_{624}$

Click to enlarge

**Physico-chemical properties**
**Hydrophobicity <H>** 0.308

**Polar residues + GLY**
**Polar residues + GLY (n / %)** 8 / 44.44

**Nonpolar residues**
**Nonpolar residues (n / %)** 10 / 55.56

**Hydrophobic moment <μH>**
0.064
**Net charge z**
1

**Uncharged residues + GLY**
SER 1, THR 1, GLY 1
**Charged residues**
LYS 3, GLU 2,
**Hydrophobic face :** none

**Aromatic residues**
TYR 1,
**Special residues**
CYS 0, PRO 3

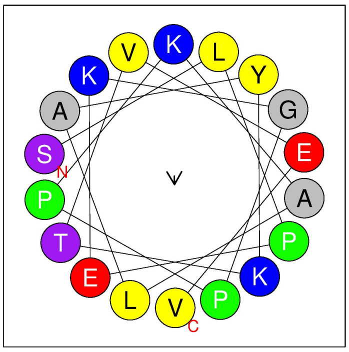

Go to screening | Manual mutation | GA mutation

---

$_{608}$**LPEKGPPKALAYKTVEVV**$_{625}$

Click to enlarge

**Physico-chemical properties**
**Hydrophobicity <H>**
0.378
**Hydrophobic moment <μH>**
0.086
**Net charge z**
1

**Polar residues + GLY**
**Polar residues + GLY (n / %)**
7 / 38.89
**Uncharged residues + GLY**
THR 1, GLY 1
**Charged residues**
LYS 3, GLU 2,
**Hydrophobic face :** none

**Nonpolar residues**
**Nonpolar residues (n / %)**
11 / 61.11
**Aromatic residues**
TYR 1,
**Special residues**
CYS 0, PRO 3

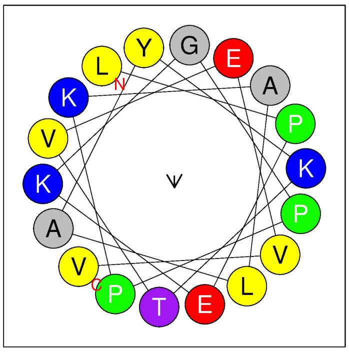

Go to screening | Manual mutation | GA mutation

---

$_{609}$**PEKGPPKALAYKTVEVVE**$_{626}$

Click to enlarge

**Physico-chemical properties**
**Hydrophobicity <H>**
0.248
**Hydrophobic moment <μH>**
0.208
**Net charge z**
0

**Polar residues + GLY**
**Polar residues + GLY (n / %)**
8 / 44.44
**Uncharged residues + GLY**
THR 1, GLY 1
**Charged residues**
LYS 3, GLU 3,
**Hydrophobic face :** none

**Nonpolar residues**
**Nonpolar residues (n / %)**
10 / 55.56
**Aromatic residues**
TYR 1,
**Special residues**
CYS 0, PRO 3

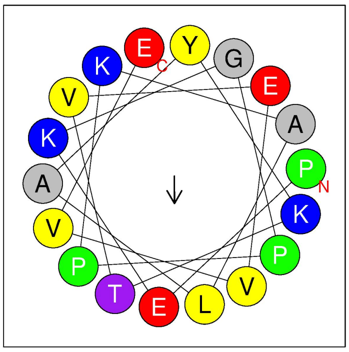

Go to screening | Manual mutation | GA mutation

---

$_{610}$**EKGPPKALAYKTVEVVES**$_{627}$

Click to enlarge

**Physico-chemical properties**
**Hydrophobicity <H>**
0.206
**Hydrophobic moment <μH>**
0.214
**Net charge z**
0

**Polar residues + GLY**
**Polar residues + GLY (n / %)**
9 / 50.00
**Uncharged residues + GLY**
SER 1, THR 1, GLY 1
**Charged residues**
LYS 3, GLU 3,
**Hydrophobic face :** none

**Nonpolar residues**
**Nonpolar residues (n / %)**
9 / 50.00
**Aromatic residues**
TYR 1,
**Special residues**
CYS 0, PRO 2

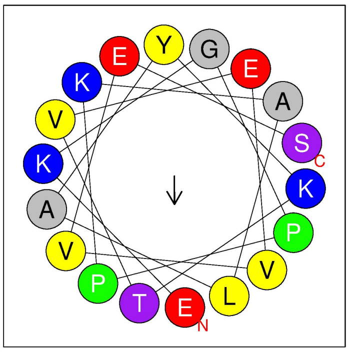

Go to screening | Manual mutation | GA mutation

---

$_{611}$**KGPPKALAYKTVEVVESI**$_{628}$

Click to enlarge

**Physico-chemical properties**
**Hydrophobicity <H>**
0.342

**Polar residues + GLY**
**Polar residues + GLY (n / %)**
8 / 44.44

**Nonpolar residues**
**Nonpolar residues (n / %)**
10 / 55.56

| | | |
|---|---|---|
| **Hydrophobic moment <μH>** | **Uncharged residues + GLY** | **Aromatic residues** |
| 0.350 | SER 1, THR 1, GLY 1 | TYR 1, |
| **Net charge z** | **Charged residues** | **Special residues** |
| 1 | LYS 3, GLU 2, | CYS 0, PRO 2 |
| | **Hydrophobic face :** none | |
| Go to screening | Manual mutation | GA mutation |

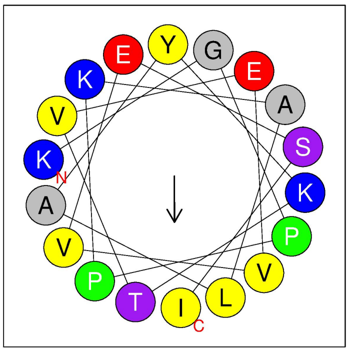

---

**$_{612}$GPPKALAYKTVEVVESIE$_{629}$**

Click to enlarge

| | | |
|---|---|---|
| **Physico-chemical properties** | **Polar residues + GLY** | **Nonpolar residues** |
| **Hydrophobicity <H>** | **Polar residues + GLY (n / %)** | **Nonpolar residues (n / %)** |
| 0.361 | 8 / 44.44 | 10 / 55.56 |
| **Hydrophobic moment <μH>** | **Uncharged residues + GLY** | **Aromatic residues** |
| 0.348 | SER 1, THR 1, GLY 1 | TYR 1, |
| **Net charge z** | **Charged residues** | **Special residues** |
| -1 | LYS 2, GLU 3, | CYS 0, PRO 2 |
| | **Hydrophobic face :** none | |
| Go to screening | Manual mutation | GA mutation |

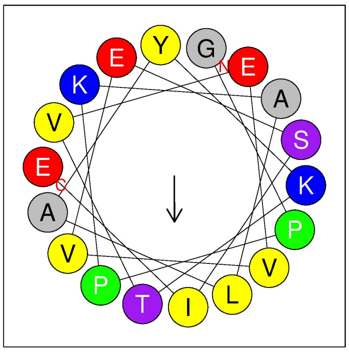

---

**$_{613}$PPKALAYKTVEVVESIEK$_{630}$**

Click to enlarge

| | | |
|---|---|---|
| **Physico-chemical properties** | **Polar residues + GLY** | **Nonpolar residues** |
| **Hydrophobicity <H>** | **Polar residues + GLY (n / %)** | **Nonpolar residues (n / %)** |
| 0.306 | 8 / 44.44 | 10 / 55.56 |
| **Hydrophobic moment <μH>** | **Uncharged residues + GLY** | **Aromatic residues** |
| 0.401 | SER 1, THR 1, GLY 0 | TYR 1, |
| **Net charge z** | **Charged residues** | **Special residues** |
| 0 | LYS 3, GLU 3, | CYS 0, PRO 2 |
| | **Hydrophobic face :** none | |
| Go to screening | Manual mutation | GA mutation |

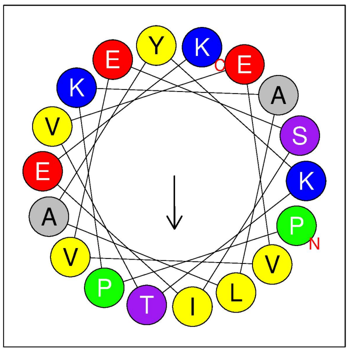

---

**$_{614}$PKALAYKTVEVVESIEKI$_{631}$**

Click to enlarge

| | | |
|---|---|---|
| **Physico-chemical properties** | **Polar residues + GLY** | **Nonpolar residues** |
| **Hydrophobicity <H>** | **Polar residues + GLY (n / %)** | **Nonpolar residues (n / %)** |
| 0.366 | 8 / 44.44 | 10 / 55.56 |
| **Hydrophobic moment <μH>** | **Uncharged residues + GLY** | **Aromatic residues** |
| 0.427 | SER 1, THR 1, GLY 0 | TYR 1, |
| **Net charge z** | **Charged residues** | **Special residues** |
| 0 | LYS 3, GLU 3, | CYS 0, PRO 1 |
| | **Hydrophobic face :** none | |
| Go to screening | Manual mutation | GA mutation |

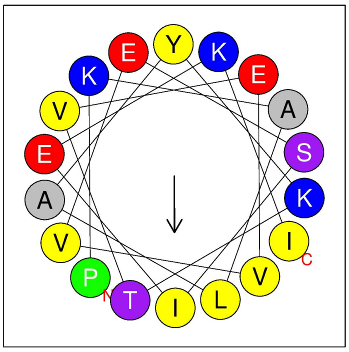

---

**$_{615}$KALAYKTVEVVESIEKIS$_{632}$**

Click to enlarge

| | | |
|---|---|---|
| **Physico-chemical properties** | **Polar residues + GLY** | **Nonpolar residues** |
| **Hydrophobicity <H>** | **Polar residues + GLY (n / %)** | **Nonpolar residues (n / %)** |
| 0.324 | 9 / 50.00 | 9 / 50.00 |

**Hydrophobic moment <μH>**
0.396
**Net charge z**
0

**Uncharged residues + GLY**
SER 2, THR 1, GLY 0
**Charged residues**
LYS 3, GLU 3,
**Hydrophobic face :** none

**Aromatic residues**
TYR 1,
**Special residues**
CYS 0, PRO 0

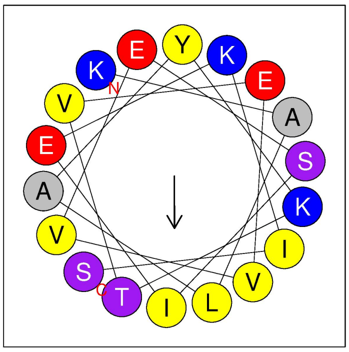

Go to screening | Manual mutation | GA mutation

---

$_{616}$**ALAYKTVEVVESIEKIST**$_{633}$

Click to enlarge

**Physico-chemical properties**
**Hydrophobicity <H>**
0.393
**Hydrophobic moment <μH>**
0.342
**Net charge z**
-1

**Polar residues + GLY**
**Polar residues + GLY (n / %)**
9 / 50.00
**Uncharged residues + GLY**
SER 2, THR 2, GLY 0
**Charged residues**
LYS 2, GLU 3,
**Hydrophobic face :** none

**Nonpolar residues**
**Nonpolar residues (n / %)**
9 / 50.00
**Aromatic residues**
TYR 1,
**Special residues**
CYS 0, PRO 0

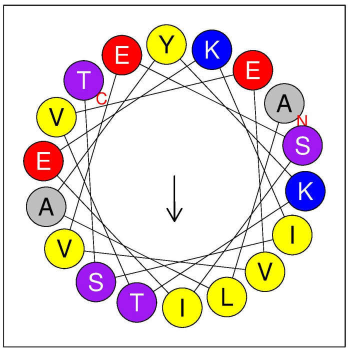

Go to screening | Manual mutation | GA mutation

---

$_{617}$**LAYKTVEVVESIEKISTE**$_{634}$

Click to enlarge

**Physico-chemical properties**
**Hydrophobicity <H>**
0.341
**Hydrophobic moment <μH>**
0.374
**Net charge z**
-2

**Polar residues + GLY**
**Polar residues + GLY (n / %)**
10 / 55.56
**Uncharged residues + GLY**
SER 2, THR 2, GLY 0
**Charged residues**
LYS 2, GLU 4,
**Hydrophobic face :** none

**Nonpolar residues**
**Nonpolar residues (n / %)**
8 / 44.44
**Aromatic residues**
TYR 1,
**Special residues**
CYS 0, PRO 0

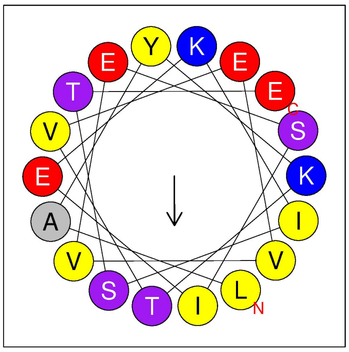

Go to screening | Manual mutation | GA mutation

---

$_{618}$**AYKTVEVVESIEKISTES**$_{635}$

Click to enlarge

**Physico-chemical properties**
**Hydrophobicity <H>**
0.244
**Hydrophobic moment <μH>**
0.295
**Net charge z**
-2

**Polar residues + GLY**
**Polar residues + GLY (n / %)**
11 / 61.11
**Uncharged residues + GLY**
SER 3, THR 2, GLY 0
**Charged residues**
LYS 2, GLU 4,
**Hydrophobic face :** none

**Nonpolar residues**
**Nonpolar residues (n / %)**
7 / 38.89
**Aromatic residues**
TYR 1,
**Special residues**
CYS 0, PRO 0

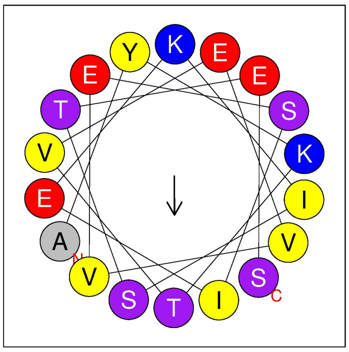

Go to screening | Manual mutation | GA mutation

---

$_{619}$**YKTVEVVESIEKISTESI**$_{636}$

Click to enlarge

**Physico-chemical properties**
**Hydrophobicity <H>**
0.327

**Polar residues + GLY**
**Polar residues + GLY (n / %)**
11 / 61.11

**Nonpolar residues**
**Nonpolar residues (n / %)**
7 / 38.89

**Hydrophobic moment <μH>**
0.343
**Net charge z**
-2

**Uncharged residues + GLY**
SER 3, THR 2, GLY 0
**Charged residues**
LYS 2, GLU 4,
**Hydrophobic face :** none

**Aromatic residues**
TYR 1,
**Special residues**
CYS 0, PRO 0

Go to screening | Manual mutation | GA mutation

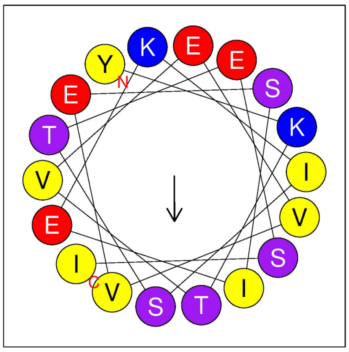


---

Click to enlarge

**$_{620}$KTVEVVESIEKISTESIQ$_{637}$**

**Physico-chemical properties**
**Hydrophobicity <H>**
0.261
**Hydrophobic moment <μH>**
0.401
**Net charge z**
-2

**Polar residues + GLY**
**Polar residues + GLY (n / %)**
12 / 66.67
**Uncharged residues + GLY**
GLN 1, SER 3, THR 2, GLY 0
**Charged residues**
LYS 2, GLU 4,
**Hydrophobic face :** none

**Nonpolar residues**
**Nonpolar residues (n / %)**
6 / 33.33
**Aromatic residues**

**Special residues**
CYS 0, PRO 0

Go to screening | Manual mutation | GA mutation

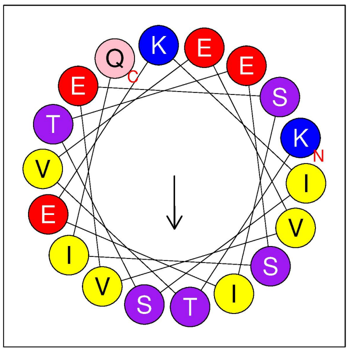


---

Click to enlarge

**$_{621}$TVEVVESIEKISTESIQT$_{638}$**

**Physico-chemical properties**
**Hydrophobicity <H>**
0.331
**Hydrophobic moment <μH>**
0.386
**Net charge z**
-3

**Polar residues + GLY**
**Polar residues + GLY (n / %)**
12 / 66.67
**Uncharged residues + GLY**
GLN 1, SER 3, THR 3, GLY 0
**Charged residues**
LYS 1, GLU 4,
**Hydrophobic face :** none

**Nonpolar residues**
**Nonpolar residues (n / %)**
6 / 33.33
**Aromatic residues**

**Special residues**
CYS 0, PRO 0

Go to screening | Manual mutation | GA mutation

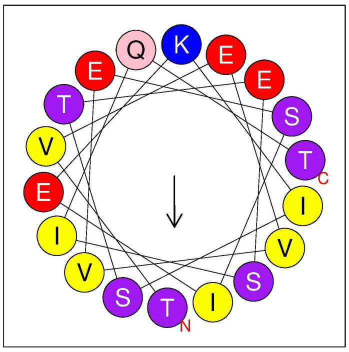


---

Click to enlarge

**$_{622}$VEVVESIEKISTESIQTY$_{639}$**

**Physico-chemical properties**
**Hydrophobicity <H>**
0.369
**Hydrophobic moment <μH>**
0.425
**Net charge z**
-3

**Polar residues + GLY**
**Polar residues + GLY (n / %)**
11 / 61.11
**Uncharged residues + GLY**
GLN 1, SER 3, THR 2, GLY 0
**Charged residues**
LYS 1, GLU 4,
**Hydrophobic face :** none

**Nonpolar residues**
**Nonpolar residues (n / %)**
7 / 38.89
**Aromatic residues**
TYR 1,
**Special residues**
CYS 0, PRO 0

Go to screening | Manual mutation | GA mutation

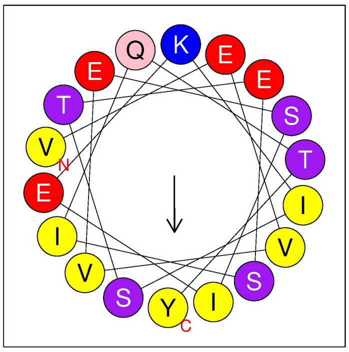


---

Click to enlarge

**$_{623}$EVVESIEKISTESIQTYE$_{640}$**

**Physico-chemical properties**
**Hydrophobicity <H>**
0.266

**Polar residues + GLY**
**Polar residues + GLY (n / %)**
12 / 66.67

**Nonpolar residues**
**Nonpolar residues (n / %)**
6 / 33.33

**Hydrophobic moment <μH>**
0.459
**Net charge z**
-4

**Uncharged residues + GLY**
GLN 1, SER 3, THR 2, GLY 0
**Charged residues**
LYS 1, GLU 5,
**Hydrophobic face :** none

**Aromatic residues**
TYR 1,
**Special residues**
CYS 0, PRO 0

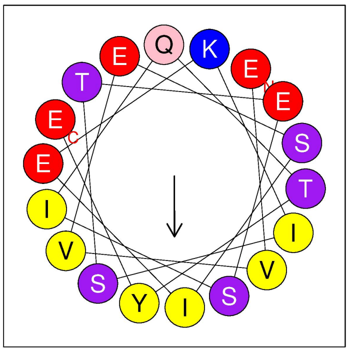


Go to screening | Manual mutation | GA mutation

---

$_{624}$**VVESIEKISTESIQTYEE**$_{641}$

Click to enlarge

**Physico-chemical properties**
**Hydrophobicity <H>**
0.266
**Hydrophobic moment <μH>**
0.459
**Net charge z**
-4

**Polar residues + GLY**
**Polar residues + GLY (n / %)**
12 / 66.67
**Uncharged residues + GLY**
GLN 1, SER 3, THR 2, GLY 0
**Charged residues**
LYS 1, GLU 5,
**Hydrophobic face :** none

**Nonpolar residues**
**Nonpolar residues (n / %)**
6 / 33.33
**Aromatic residues**
TYR 1,
**Special residues**
CYS 0, PRO 0

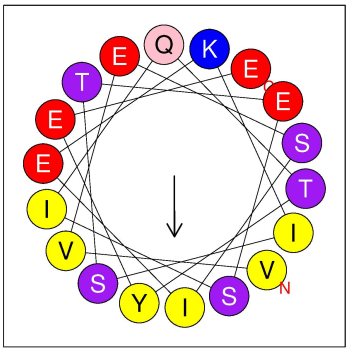


Go to screening | Manual mutation | GA mutation

---

$_{625}$**VESIEKISTESIQTYEET**$_{642}$

Click to enlarge

**Physico-chemical properties**
**Hydrophobicity <H>**
0.213
**Hydrophobic moment <μH>**
0.423
**Net charge z**
-4

**Polar residues + GLY**
**Polar residues + GLY (n / %)**
13 / 72.22
**Uncharged residues + GLY**
GLN 1, SER 3, THR 3, GLY 0
**Charged residues**
LYS 1, GLU 5,
**Hydrophobic face :** none

**Nonpolar residues**
**Nonpolar residues (n / %)**
5 / 27.78
**Aromatic residues**
TYR 1,
**Special residues**
CYS 0, PRO 0

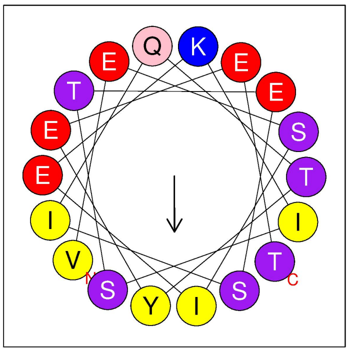


Go to screening | Manual mutation | GA mutation

---

$_{626}$**ESIEKISTESIQTYEETA**$_{643}$

Click to enlarge

**Physico-chemical properties**
**Hydrophobicity <H>**
0.162
**Hydrophobic moment <μH>**
0.392
**Net charge z**
-4

**Polar residues + GLY**
**Polar residues + GLY (n / %)**
13 / 72.22
**Uncharged residues + GLY**
GLN 1, SER 3, THR 3, GLY 0
**Charged residues**
LYS 1, GLU 5,
**Hydrophobic face :** none

**Nonpolar residues**
**Nonpolar residues (n / %)**
5 / 27.78
**Aromatic residues**
TYR 1,
**Special residues**
CYS 0, PRO 0

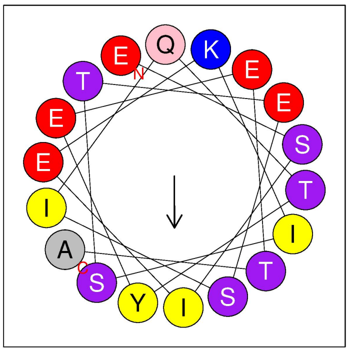


Go to screening | Manual mutation | GA mutation

---

$_{627}$**SIEKISTESIQTYEETAV**$_{644}$

Click to enlarge

**Physico-chemical properties**
**Hydrophobicity <H>**
0.266

**Polar residues + GLY**
**Polar residues + GLY (n / %)**
12 / 66.67

**Nonpolar residues**
**Nonpolar residues (n / %)**
6 / 33.33

| | | |
|---|---|---|
| **Hydrophobic moment <μH>** | **Uncharged residues + GLY** | **Aromatic residues** |
| 0.301 | GLN 1, SER 3, THR 3, GLY 0 | TYR 1, |
| **Net charge z** | **Charged residues** | **Special residues** |
| -3 | LYS 1, GLU 4, | CYS 0, PRO 0 |
| | **Hydrophobic face :** none | |
| Go to screening | Manual mutation | GA mutation |

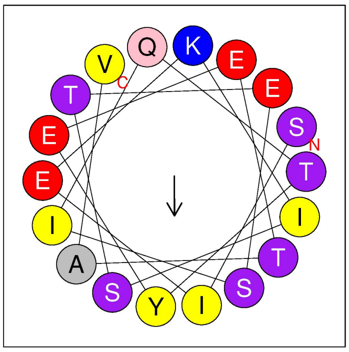

---

### $_{628}$IEKISTESIQTYEETAVI$_{645}$

Click to enlarge

| | | |
|---|---|---|
| **Physico-chemical properties** | **Polar residues + GLY** | **Nonpolar residues** |
| **Hydrophobicity <H>** | **Polar residues + GLY (n / %)** | **Nonpolar residues (n / %)** |
| 0.368 | 11 / 61.11 | 7 / 38.89 |
| **Hydrophobic moment <μH>** | **Uncharged residues + GLY** | **Aromatic residues** |
| 0.279 | GLN 1, SER 2, THR 3, GLY 0 | TYR 1, |
| **Net charge z** | **Charged residues** | **Special residues** |
| -3 | LYS 1, GLU 4, | CYS 0, PRO 0 |
| | **Hydrophobic face :** none | |
| Go to screening | Manual mutation | GA mutation |

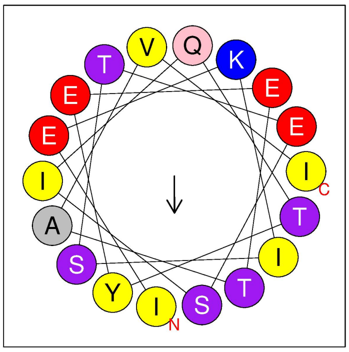

---

### $_{629}$EKISTESIQTYEETAVIV$_{646}$

Click to enlarge

| | | |
|---|---|---|
| **Physico-chemical properties** | **Polar residues + GLY** | **Nonpolar residues** |
| **Hydrophobicity <H>** | **Polar residues + GLY (n / %)** | **Nonpolar residues (n / %)** |
| 0.336 | 11 / 61.11 | 7 / 38.89 |
| **Hydrophobic moment <μH>** | **Uncharged residues + GLY** | **Aromatic residues** |
| 0.247 | GLN 1, SER 2, THR 3, GLY 0 | TYR 1, |
| **Net charge z** | **Charged residues** | **Special residues** |
| -3 | LYS 1, GLU 4, | CYS 0, PRO 0 |
| | **Hydrophobic face :** none | |
| Go to screening | Manual mutation | GA mutation |

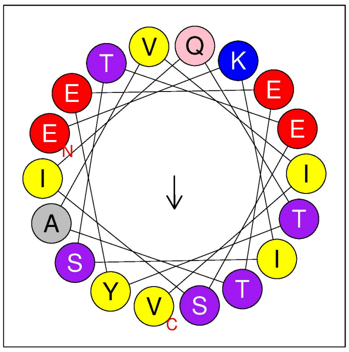

---

### $_{630}$KISTESIQTYEETAVIVE$_{647}$

Click to enlarge

| | | |
|---|---|---|
| **Physico-chemical properties** | **Polar residues + GLY** | **Nonpolar residues** |
| **Hydrophobicity <H>** | **Polar residues + GLY (n / %)** | **Nonpolar residues (n / %)** |
| 0.336 | 11 / 61.11 | 7 / 38.89 |
| **Hydrophobic moment <μH>** | **Uncharged residues + GLY** | **Aromatic residues** |
| 0.247 | GLN 1, SER 2, THR 3, GLY 0 | TYR 1, |
| **Net charge z** | **Charged residues** | **Special residues** |
| -3 | LYS 1, GLU 4, | CYS 0, PRO 0 |
| | **Hydrophobic face :** none | |
| Go to screening | Manual mutation | GA mutation |

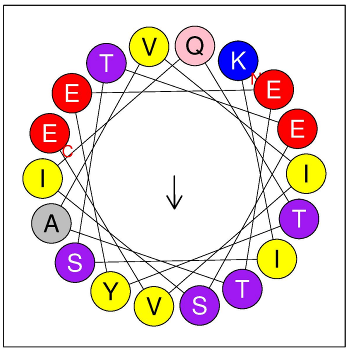

---

### $_{631}$ISTESIQTYEETAVIVET$_{648}$

Click to enlarge

| | | |
|---|---|---|
| **Physico-chemical properties** | **Polar residues + GLY** | **Nonpolar residues** |
| **Hydrophobicity <H>** | **Polar residues + GLY (n / %)** | **Nonpolar residues (n / %)** |
| 0.405 | 11 / 61.11 | 7 / 38.89 |

**Hydrophobic moment <μH>**
0.190
**Net charge z**
-4

**Uncharged residues + GLY**
GLN 1, SER 2, THR 4, GLY 0
**Charged residues**
GLU 4,
**Hydrophobic face :** none

**Aromatic residues**
TYR 1,
**Special residues**
CYS 0, PRO 0

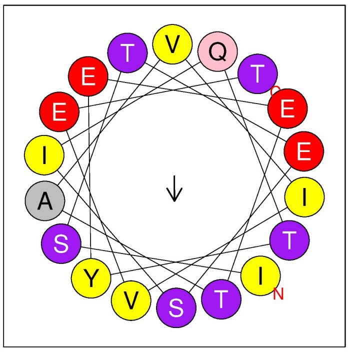


Go to screening
Manual mutation
GA mutation

---

**$_{632}$STESIQTYEETAVIVETM$_{649}$**

Click to enlarge

**Physico-chemical properties**
**Hydrophobicity <H>**
0.373
**Hydrophobic moment <μH>**
0.167
**Net charge z**
-4

**Polar residues + GLY**
**Polar residues + GLY (n / %)**
11 / 61.11
**Uncharged residues + GLY**
GLN 1, SER 2, THR 4, GLY 0
**Charged residues**
GLU 4,
**Hydrophobic face :** none

**Nonpolar residues**
**Nonpolar residues (n / %)**
7 / 38.89
**Aromatic residues**
TYR 1,
**Special residues**
CYS 0, PRO 0

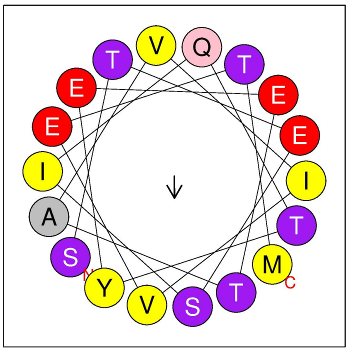


Go to screening
Manual mutation
GA mutation

---

**$_{633}$TESIQTYEETAVIVETMI$_{650}$**

Click to enlarge

**Physico-chemical properties**
**Hydrophobicity <H>**
0.476
**Hydrophobic moment <μH>**
0.244
**Net charge z**
-4

**Polar residues + GLY**
**Polar residues + GLY (n / %)**
10 / 55.56
**Uncharged residues + GLY**
GLN 1, SER 1, THR 4, GLY 0
**Charged residues**
GLU 4,
**Hydrophobic face :** V Y I A I

**Nonpolar residues**
**Nonpolar residues (n / %)**
8 / 44.44
**Aromatic residues**
TYR 1,
**Special residues**
CYS 0, PRO 0

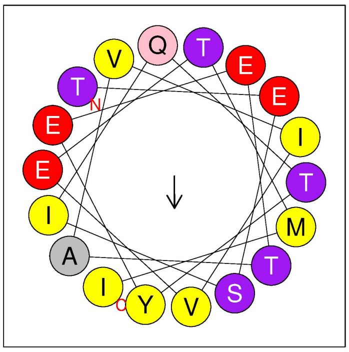


Go to screening
Manual mutation
GA mutation

---

**$_{634}$ESIQTYEETAVIVETMIG$_{651}$**

Click to enlarge

**Physico-chemical properties**
**Hydrophobicity <H>**
0.461
**Hydrophobic moment <μH>**
0.254
**Net charge z**
-4

**Polar residues + GLY**
**Polar residues + GLY (n / %)**
10 / 55.56
**Uncharged residues + GLY**
GLN 1, SER 1, THR 3, GLY 1
**Charged residues**
GLU 4,
**Hydrophobic face :** V Y I A I

**Nonpolar residues**
**Nonpolar residues (n / %)**
8 / 44.44
**Aromatic residues**
TYR 1,
**Special residues**
CYS 0, PRO 0

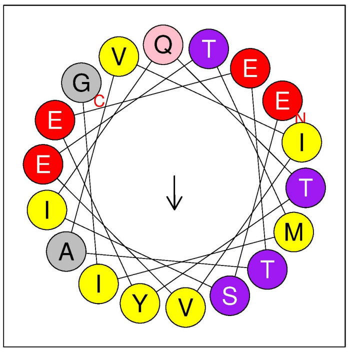


Go to screening
Manual mutation
GA mutation

---

**$_{635}$SIQTYEETAVIVETMIGK$_{652}$**

Click to enlarge

**Physico-chemical properties**
**Hydrophobicity <H>**
0.442

**Polar residues + GLY**
**Polar residues + GLY (n / %)**
10 / 55.56

**Nonpolar residues**
**Nonpolar residues (n / %)**
8 / 44.44

| | | |
|---|---|---|
| **Hydrophobic moment <μH>** | **Uncharged residues + GLY** | **Aromatic residues** |
| 0.265 | GLN 1, SER 1, THR 3, GLY 1 | TYR 1, |
| **Net charge z** | **Charged residues** | **Special residues** |
| -2 | LYS 1, GLU 3, | CYS 0, PRO 0 |
| | **Hydrophobic face :** V Y I A I | |
| Go to screening | Manual mutation | GA mutation |

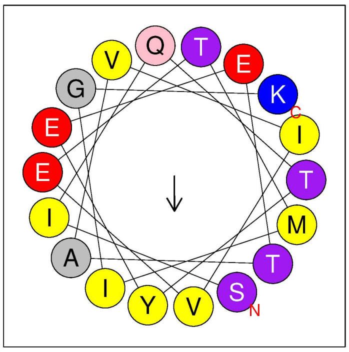


---

## $_{636}$IQTYEETAVIVETMIGKT$_{653}$

Click to enlarge

| | | |
|---|---|---|
| **Physico-chemical properties** | **Polar residues + GLY** | **Nonpolar residues** |
| **Hydrophobicity <H>** | **Polar residues + GLY (n / %)** | **Nonpolar residues (n / %)** |
| 0.458 | 10 / 55.56 | 8 / 44.44 |
| **Hydrophobic moment <μH>** | **Uncharged residues + GLY** | **Aromatic residues** |
| 0.280 | GLN 1, THR 4, GLY 1 | TYR 1, |
| **Net charge z** | **Charged residues** | **Special residues** |
| -2 | LYS 1, GLU 3, | CYS 0, PRO 0 |
| | **Hydrophobic face :** V Y I A I | |
| Go to screening | Manual mutation | GA mutation |

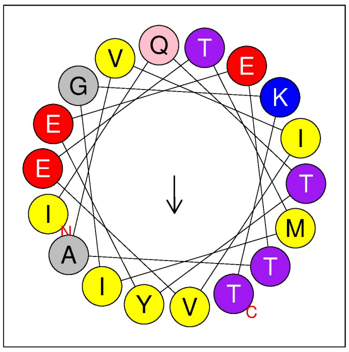


---

## $_{637}$QTYEETAVIVETMIGKTK$_{654}$

Click to enlarge

| | | |
|---|---|---|
| **Physico-chemical properties** | **Polar residues + GLY** | **Nonpolar residues** |
| **Hydrophobicity <H>** | **Polar residues + GLY (n / %)** | **Nonpolar residues (n / %)** |
| 0.303 | 11 / 61.11 | 7 / 38.89 |
| **Hydrophobic moment <μH>** | **Uncharged residues + GLY** | **Aromatic residues** |
| 0.278 | GLN 1, THR 4, GLY 1 | TYR 1, |
| **Net charge z** | **Charged residues** | **Special residues** |
| -1 | LYS 2, GLU 3, | CYS 0, PRO 0 |
| | **Hydrophobic face :** none | |
| Go to screening | Manual mutation | GA mutation |

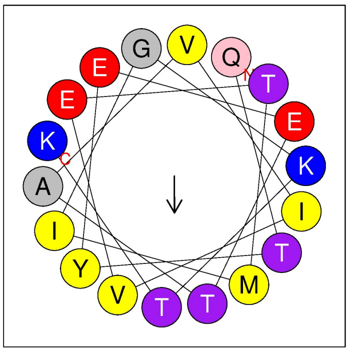


---

## $_{638}$TYEETAVIVETMIGKTKS$_{655}$

Click to enlarge

| | | |
|---|---|---|
| **Physico-chemical properties** | **Polar residues + GLY** | **Nonpolar residues** |
| **Hydrophobicity <H>** | **Polar residues + GLY (n / %)** | **Nonpolar residues (n / %)** |
| 0.313 | 11 / 61.11 | 7 / 38.89 |
| **Hydrophobic moment <μH>** | **Uncharged residues + GLY** | **Aromatic residues** |
| 0.269 | SER 1, THR 4, GLY 1 | TYR 1, |
| **Net charge z** | **Charged residues** | **Special residues** |
| -1 | LYS 2, GLU 3, | CYS 0, PRO 0 |
| | **Hydrophobic face :** none | |
| Go to screening | Manual mutation | GA mutation |

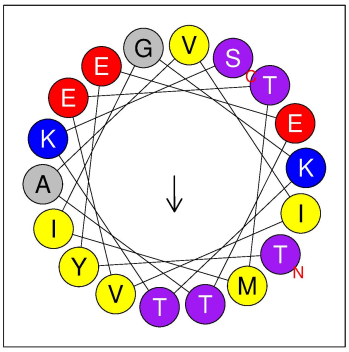


---

## $_{639}$YEETAVIVETMIGKTKSD$_{656}$

Click to enlarge

| | | |
|---|---|---|
| **Physico-chemical properties** | **Polar residues + GLY** | **Nonpolar residues** |
| **Hydrophobicity <H>** | **Polar residues + GLY (n / %)** | **Nonpolar residues (n / %)** |
| 0.256 | 11 / 61.11 | 7 / 38.89 |

**Hydrophobic moment <μH>**
0.240
**Net charge z**
-2

**Uncharged residues + GLY**
SER 1, THR 3, GLY 1
**Charged residues**
LYS 2, GLU 3, ASP 1,
**Hydrophobic face :** none

**Aromatic residues**
TYR 1,
**Special residues**
CYS 0, PRO 0

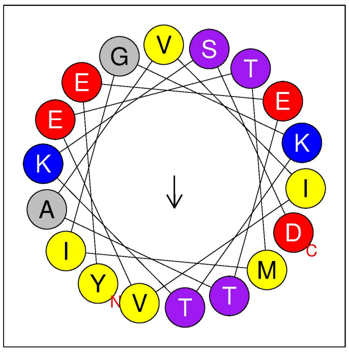


Go to screening
Manual mutation
GA mutation

---

$_{640}$**EETAVIVETMIGKTKSDK**$_{657}$

Click to enlarge

**Physico-chemical properties**
**Hydrophobicity <H>**
0.148
**Hydrophobic moment <μH>**
0.164
**Net charge z**
-1

**Polar residues + GLY**
**Polar residues + GLY (n / %)**
12 / 66.67
**Uncharged residues + GLY**
SER 1, THR 3, GLY 1
**Charged residues**
LYS 3, GLU 3, ASP 1,
**Hydrophobic face :** none

**Nonpolar residues**
**Nonpolar residues (n / %)**
6 / 33.33
**Aromatic residues**

**Special residues**
CYS 0, PRO 0

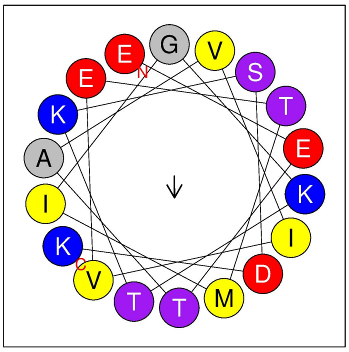


Go to screening
Manual mutation
GA mutation

---

$_{641}$**ETAVIVETMIGKTKSDKK**$_{658}$

Click to enlarge

**Physico-chemical properties**
**Hydrophobicity <H>**
0.128
**Hydrophobic moment <μH>**
0.182
**Net charge z**
1

**Polar residues + GLY**
**Polar residues + GLY (n / %)**
12 / 66.67
**Uncharged residues + GLY**
SER 1, THR 3, GLY 1
**Charged residues**
LYS 4, GLU 2, ASP 1,
**Hydrophobic face :** none

**Nonpolar residues**
**Nonpolar residues (n / %)**
6 / 33.33
**Aromatic residues**

**Special residues**
CYS 0, PRO 0

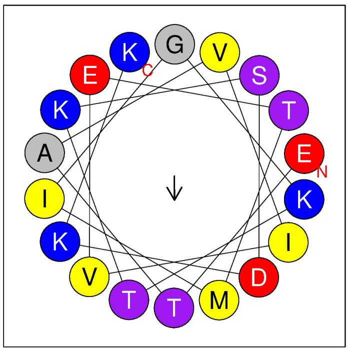


Go to screening
Manual mutation
GA mutation

---

$_{642}$**TAVIVETMIGKTKSDKKK**$_{659}$

Click to enlarge

**Physico-chemical properties**
**Hydrophobicity <H>**
0.109
**Hydrophobic moment <μH>**
0.186
**Net charge z**
3

**Polar residues + GLY**
**Polar residues + GLY (n / %)**
12 / 66.67
**Uncharged residues + GLY**
SER 1, THR 3, GLY 1
**Charged residues**
LYS 5, GLU 1, ASP 1,
**Hydrophobic face :** none

**Nonpolar residues**
**Nonpolar residues (n / %)**
6 / 33.33
**Aromatic residues**

**Special residues**
CYS 0, PRO 0

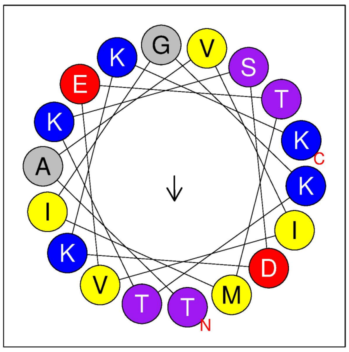


Go to screening
Manual mutation
GA mutation

---

$_{643}$**AVIVETMIGKTKSDKKKS**$_{660}$

Click to enlarge

**Physico-chemical properties**
**Hydrophobicity <H>**
0.092

**Polar residues + GLY**
**Polar residues + GLY (n / %)**
12 / 66.67

**Nonpolar residues**
**Nonpolar residues (n / %)**
6 / 33.33

| | | |
|---|---|---|
| **Hydrophobic moment <μH>** | **Uncharged residues + GLY** | **Aromatic residues** |
| 0.170 | SER 2, THR 2, GLY 1 | |
| **Net charge z** | **Charged residues** | **Special residues** |
| 3 | LYS 5, GLU 1, ASP 1, | CYS 0, PRO 0 |
| | **Hydrophobic face :** none | |
| Go to screening | Manual mutation | GA mutation |

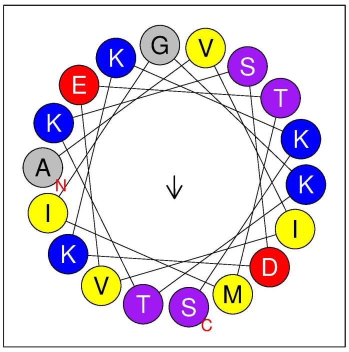

---

$_{644}$**VIVETMIGKTKSDKKKSG**$_{661}$

Click to enlarge

| | | |
|---|---|---|
| **Physico-chemical properties** | **Polar residues + GLY** | **Nonpolar residues** |
| **Hydrophobicity <H>** | **Polar residues + GLY (n / %)** | **Nonpolar residues (n / %)** |
| 0.075 | 13 / 72.22 | 5 / 27.78 |
| **Hydrophobic moment <μH>** | **Uncharged residues + GLY** | **Aromatic residues** |
| 0.172 | SER 2, THR 2, GLY 2 | |
| **Net charge z** | **Charged residues** | **Special residues** |
| 3 | LYS 5, GLU 1, ASP 1, | CYS 0, PRO 0 |
| | **Hydrophobic face :** none | |
| Go to screening | Manual mutation | GA mutation |

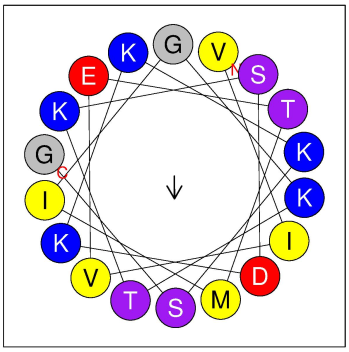

---

$_{645}$**IVETMIGKTKSDKKKSGE**$_{662}$

Click to enlarge

| | | |
|---|---|---|
| **Physico-chemical properties** | **Polar residues + GLY** | **Nonpolar residues** |
| **Hydrophobicity <H>** | **Polar residues + GLY (n / %)** | **Nonpolar residues (n / %)** |
| -0.028 | 14 / 77.78 | 4 / 22.22 |
| **Hydrophobic moment <μH>** | **Uncharged residues + GLY** | **Aromatic residues** |
| 0.271 | SER 2, THR 2, GLY 2 | |
| **Net charge z** | **Charged residues** | **Special residues** |
| 2 | LYS 5, GLU 2, ASP 1, | CYS 0, PRO 0 |
| | **Hydrophobic face :** none | |
| Go to screening | Manual mutation | GA mutation |

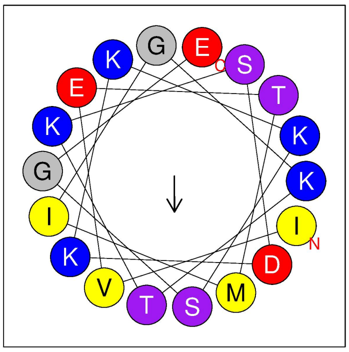

---

$_{646}$**VETMIGKTKSDKKKSGEK**$_{663}$

Click to enlarge

| | | |
|---|---|---|
| **Physico-chemical properties** | **Polar residues + GLY** | **Nonpolar residues** |
| **Hydrophobicity <H>** | **Polar residues + GLY (n / %)** | **Nonpolar residues (n / %)** |
| -0.183 | 15 / 83.33 | 3 / 16.67 |
| **Hydrophobic moment <μH>** | **Uncharged residues + GLY** | **Aromatic residues** |
| 0.257 | SER 2, THR 2, GLY 2 | |
| **Net charge z** | **Charged residues** | **Special residues** |
| 3 | LYS 6, GLU 2, ASP 1, | CYS 0, PRO 0 |
| | **Hydrophobic face :** none | |
| Go to screening | Manual mutation | GA mutation |

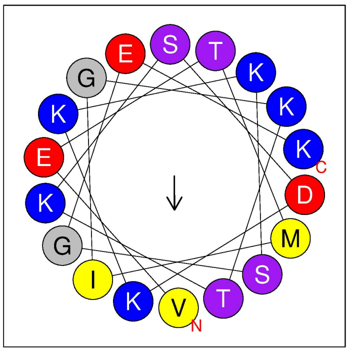

---

$_{647}$**ETMIGKTKSDKKKSGEKS**$_{664}$

Click to enlarge

| | | |
|---|---|---|
| **Physico-chemical properties** | **Polar residues + GLY** | **Nonpolar residues** |
| **Hydrophobicity <H>** | **Polar residues + GLY (n / %)** | **Nonpolar residues (n / %)** |
| -0.253 | 16 / 88.89 | 2 / 11.11 |

**Hydrophobic moment <μH>**
0.187
**Net charge z**
3

**Uncharged residues + GLY**
SER 3, THR 2, GLY 2
**Charged residues**
LYS 6, GLU 2, ASP 1,
**Hydrophobic face :** none

**Aromatic residues**

**Special residues**
CYS 0, PRO 0

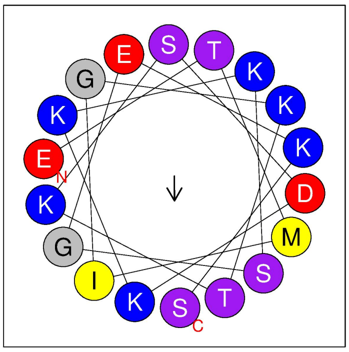

Go to screening | Manual mutation | GA mutation

---

$_{648}$**TMIGKTKSDKKKSGEKSS**$_{665}$

Click to enlarge

**Physico-chemical properties**
**Hydrophobicity <H>**
-0.220
**Hydrophobic moment <μH>**
0.185
**Net charge z**
4

**Polar residues + GLY**
**Polar residues + GLY (n / %)**
16 / 88.89
**Uncharged residues + GLY**
SER 4, THR 2, GLY 2
**Charged residues**
LYS 6, GLU 1, ASP 1,
**Hydrophobic face :** none

**Nonpolar residues**
**Nonpolar residues (n / %)**
2 / 11.11
**Aromatic residues**

**Special residues**
CYS 0, PRO 0

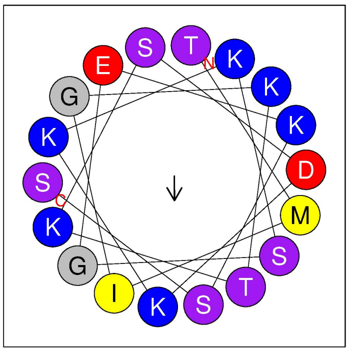

Go to screening | Manual mutation | GA mutation

---

**Hydrophobicity and Hydrophobic moment**

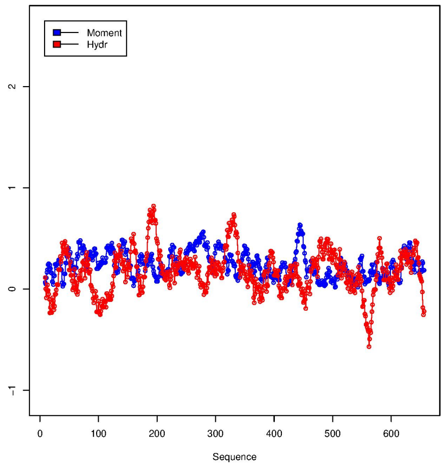


---

**Data file (in txt format):** Data.txt

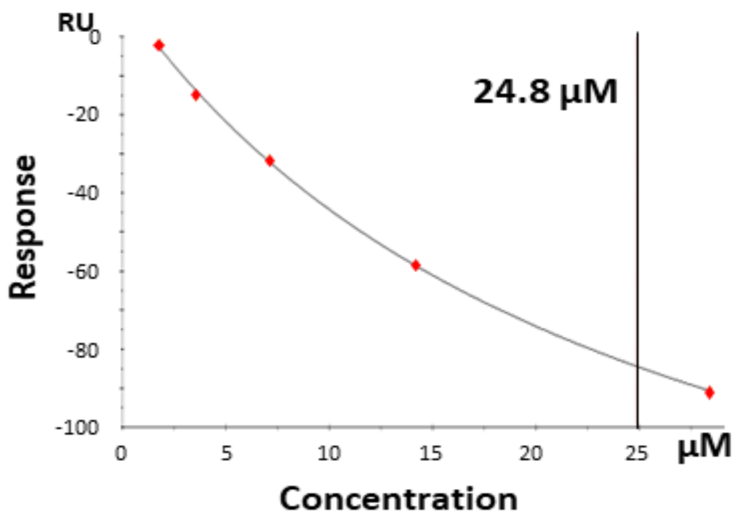


*Supplementary Figure S4: Affinity of recombinant human CRYAB for bovine lens lipids as measured by SPR.*

Recombinant human CRYAB was assessed for its affinity to the same L1 chip coated with lipids prepared from bovine lens membranes. An affinity of 22.83µM was measured.
